# Synchrotron radiation macromolecular crystallography: science and spin-offs

**DOI:** 10.1107/S205225251402795X

**Published:** 2015-02-03

**Authors:** John R. Helliwell, Edward P. Mitchell

**Affiliations:** a School of Chemistry, University of Manchester, Brunswick Street, Manchester M13 9PL, England; b ESRF, 71 avenue des Martyrs, 38000 Grenoble, France; c Faculty of Natural Sciences, Keele University, Staffordshire ST5 5BG, England

**Keywords:** automation, microcrystals, storage-ring upgrades, X-ray lasers, neutrons, industrial and commercial access, expanding wavelength range, time-resolved studies, dynamics, diffuse scattering, room-temperature studies, raw data

## Abstract

A current overview of synchrotron radiation (SR) in macromolecular crystallography (MX) instrumentation, methods and applications is presented. Automation has been and remains a central development in the last decade, as have the rise of remote access and of industrial service provision. Results include a high number of Protein Data Bank depositions, with an increasing emphasis on the successful use of microcrystals. One future emphasis involves pushing the frontiers of using higher and lower photon energies. With the advent of X-ray free-electron lasers, closely linked to SR developments, the use of ever smaller samples such as nanocrystals, nanoclusters and single molecules is anticipated, as well as the opening up of femtosecond time-resolved diffraction structural studies. At SR sources, a very high-throughput assessment for the best crystal samples and the ability to tackle just a few micron and sub-micron crystals will become widespread. With higher speeds and larger detectors, diffraction data volumes are becoming long-term storage and archiving issues; the implications for today and the future are discussed. Together with the rise of the storage ring to its current pre-eminence in MX data provision, the growing tendency of central facility sites to offer other centralized facilities complementary to crystallography, such as cryo-electron microscopy and NMR, is a welcome development.

## Abstract 2

A topical review is presented focusing on the recent developments in synchrotron radiation macromolecular crystallography. The review covers source and beamline developments, and applications including spin-offs into other areas of science and the impact upon industrial interests.

## 1. Introduction   

Synchrotron radiation (SR) has had a profound impact on the field of protein crystallography, with approximately 90% of X-ray single-crystal structure determinations being from synchrotrons (see http://biosync.sbkb.org). Compared with laboratory-based X-ray sources, the synchrotron properties of high spectral brightness and tuneability have enabled higher-resolution structure determinations, a greater use of multiple-wavelength anomalous dispersion (MAD) phasing techniques, studies of much larger molecular weight structures, the use of small crystals and time-resolved structural studies. Thus, a great deal of flexibility and adaptability of the technique to the needs of biological research now exists. An extensive summary up to 2010 of SR macromolecular crystallography (MX) and the anticipated future of X-ray lasers in structural biology is given by Duke & Johnson (2010 ▶). The topical review presented here concentrates on developments since then. A recent comprehensive overview of phasing methods in crystallography including MX and the use of multiple and single wavelength methods, including in a historical context, is given in the book by Giacovazzo (2013) ▶.

Recently, Abad-Zapatero (2014 ▶) undertook an analysis of the growth rate in Protein Data Bank (PDB; Berman *et al.*, 2000 ▶; http://www.rcsb.org/pdb/) depositions over the decades, reporting that the appearance of third-generation facilities, beginning with the ESRF (Grenoble, France) in 1994, has helped to maintain PDB data-deposition rates which otherwise might well have slowed down as more and more complex ‘molecular machines’ were studied. The last four years have seen the maturing of MX data collection and data processing at third-generation synchrotron beamlines into a high-throughput and largely automated technique. This is the culmination of a long period of development in hardware and software, and in user community culture, leading to the success of synchrotron-based MX. This success has led to further Nobel prizes in the field.

## 2. SR sources   

Third-generation SR facilities have had a major impact on the expansion of MX capabilities. They have a long history of development and, as early as 1979, plans were being put forward for a high spectral brightness insertion-device-driven European synchrotron radiation source. The so-called *ESRF Foundation Phase Report* ‘Red Book’, published in 1987, described specifications for the first third-generation source, the European Synchrotron Radiation Facility (ESRF) in Grenoble, France at 5 then 6 GeV. Proposals for the USA machine, the Advanced Photon Source (APS; Argonne, USA) at 7 GeV, and the Japanese 8 GeV SPring-8 machine (Hyogo, Japan) followed. The initial instruments for MX at the ESRF were a shared undulator high-flux (later relabelled high-brilliance) beamline well suited to virus crystallography, a shared microfocus beamline, a shared time-resolved beamline for Laue protein crystallography and a dedicated bending-magnet MAD beamline, BM14. A great expansion in beam time on an undulator came with the ESRF’s Quadriga beamline complex (Wakatsuki *et al.*, 1998 ▶) dedicated to MX. Nowadays, many national third-generation SR machines have been built with life science, and especially MX, as a key justification for the investment, with >100 beamlines worldwide to choose from, serving a very large user community across academia and industry.

A widespread development has been top-up operation. This maximizes X-ray output all the time and, perhaps more importantly, beam stability is improved. For this achievement the 2013 Compton Award was made to those who pioneered it at APS, the first facility to do so, namely David E. Moncton, John N. Galayda, Michael Borland and Louis Emery. In general, this is well received by users of SR MX and can improve the accuracy in measuring weak signals (for example anomalous signals) through the enhanced stability of the beam.

The last few years have seen efforts directed at developing storage rings towards the ultimate very low emittance (near diffraction-limited) ring design (Einfeld *et al.*, 1995 ▶), with pioneering efforts made by the upcoming MAX IV facility (MAX IV, 2010 ▶). The year 2014 saw the formal approval of the ESRF’s Upgrade Phase II, paving the way to the complete replacement of the ESRF’s storage ring, which will provide increased coherence, smaller beams and greater photon flux densities. The ESRF’s White Paper (ESRF, 2012 ▶) on the subject and forthcoming ‘Orange Book’ describe the technical design of the new machine, building on the notion of upgrading to an ultimate storage ring put forward in the ESRF’s ‘Purple Book’ (ESRF, 2007 ▶). When combined with improved beamline optics, such an upgrade could lead to a flux density increase of some five orders of magnitude on the ESRF’s MX beamlines, such as ID29 (ESRF, 2012 ▶), allowing the study of very challenging tiny samples and necessitating the adoption of a multi-crystal approach to SR MX data collection and the subsequent knitting together of partial data sets. Similar upgrades are also in the pipeline for the APS (Borland, 2014 ▶) and SPring-8 (RIKEN, 2014 ▶). Other outstanding high-brightness SR sources are the operational PETRA III in Hamburg, Germany, and NSLS II at Brookhaven in the USA, which has just generated its first X-rays.

In parallel with the evolution of X-ray sources described above, X-ray free electron lasers (XFELs) have been constructed at SLAC (the LCLS; Stanford, USA) and at SPring-8 (SACLA), with the European facility (EuroXFEL) under construction at DESY in Hamburg. These machines, based upon long linear accelerators and undulator sections, have pulse lengths of ∼10 fs and a peak (*i.e.* instantaneous) spectral brightness some ten orders of magnitude larger than storage-ring-based sources, which have a higher integrated flux delivery. These two sets of advanced source developments at synchrotrons and XFELs have overlaps and complementarities for user science [see the topical review in this issue by Weckert (2015 ▶)].

Inevitably, our selection of examples here is somewhat personal and thus only illustrative.

## 3. Technical developments for SR MX   

Like most scientific techniques, SR MX undergoes continual technical developments. Thus, there are the ‘traditional’ uses, namely *de novo* crystal structure determination, higher diffraction resolution, diffraction data collection from large unit cells and from small samples but within the common theme of faster data sets, more data sets and more automation. There are the less usual applications, such as exploring higher or lower photon energies and time-resolved studies. There also remains the largely unused MX diffraction information outside the Bragg diffraction, the diffuse scattering. This article spans both the traditional and the less usual applications of SR for MX.

### 3.1. Automation   

The automation revolution created for MX by institutes like the ESRF in partnership with the EMBL-Grenoble, by Stanford Synchrotron Radiation Laboratory, and by the more recent synchrotrons such as Diamond (Didcot, UK), SOLEIL (Saclay, France) and the SLS (Swiss Light Source, Switzerland) is a key point in the continued rise of synchrotron crystallography. These robotic developments and smart software pipelines, with their database and data-delivery frameworks, are having a major impact. Reliable sample changers and automated intelligent software are routine at every synchrotron MX beamline. In Europe, the SPINE hardware standard gives consistent sample-mounting pins and bases. A new generation of pins is being developed under the banner of another European project, BioStructX, for the transport of a larger number of samples per cryo-dewar. This is needed as sample cycle times are now down to as little as a few minutes, meaning efficient users, locally or in remote-access mode, can process hundreds of samples per shift. This represents many cryo-dewars of frozen crystals needing to be transported. The higher number density is required to allow the new generation of mail-in dedicated automated beamlines like ESRF’s MASSIF (massively automated sample selection integrated facility) to have tenable logistics.

Within Europe, the *ISPYB* and *EDNA* software permit sample, data and results tracking, and protected data delivery to users (Delagenière *et al.*, 2011 ▶). Each sample is characterized and a data-collection strategy proposed to the user for approval. The system then supervises the data collection and launches automated data processing as the data arrive.

In addition to robotics for cryo-cooled samples, room-temperature screening is possible at a number of beamlines across synchrotron facilities, for example the French FIP beamline at ESRF (la Maire *et al.*, 2011 ▶) and the X06DA station at the SLS (Bingel-Erlenmeyer *et al.*, 2011 ▶) are able to use their robotics to manipulate crystallization plates and allow crystals to be tested for diffraction quality *in situ* without the need for harvesting a sample. This permits more efficient feedback on crystallization condition refinement and, sometimes, full data collection. Indeed, Diamond is constructing a facility, VMXi, dedicated to such experiments for collection from *in situ* crystallization experiments. Automation is also extending backwards from beamlines into crystal harvesting (‘fishing’) – a considerable bottleneck and still largely done by hand. This may be changing with new laser-cutting based harvesting. The CrystalDirect system developed at EMBL-Grenoble (Cipriani *et al.*, 2012 ▶) uses a smooth roboticized procedure with laser photo-ablation to excise crystals on thin films of polyimide, which are attached by the robot to the standard SPINE pin and then cryocooled ready for data collection. Together with the automation of crystallization, and of diffraction experiments on beamlines, systems like CrystalDirect will open the route to a fully hands-free procedure.

Automation allows users to exploit SR MX facilities efficiently. In the last few years this has grown to be a popular mode of access to SR MX beamlines, and it is particularly attractive (cost effective) for industry. As an illustration, the approach taken by SERCAT (Rose *et al.*, 2014 ▶), the South East Region (of the USA) Collaborative Access Team of the APS, is to have ‘outstanding staff; a stable beamline; a good end-station goniometer; a reliable automounter; fast reliable detectors; a single intuitive interface; a secure and robust web client and provide online training resources’. Across many beamlines, remote access is now *the* way to use the facility, minimizing travel time and costs and the ecological footprint of jet travel by users. At the APS, over 95% of SERCAT’s data are collected in this way (Rose *et al.*, 2014 ▶). With modern detectors the time-limiting step has become crystal mounting and alignment.

This change to very high throughput has also ushered in a move from multi-shift visits (virtual or real) to the synchrotron to single-shift ‘pop-ins’ where users, and again especially industrial users, have shorter individual visits to ensure a continual data-stream delivery into projects.

### 3.2. Ever smaller crystal volumes   

The current and still developing frontier of synchrotron microcrystallography has a long and distinguished history. An early question was whether smaller samples studied with higher X-ray intensities were feasible. The widespread implementation of mini- and microbeams and high-precision microdiffractometers to measure data from such crystals has had a wide impact in structural biology [and has, for example, been highlighted by Nobel Prize winner Brian Kobilka in *Nature* recently (Azouz, 2014 ▶)].

Ultra-rapid sampling ‘cartography’ (Bowler *et al.*, 2010 ▶) of micro-volumes in protein crystal samples to locate the best hot-spot for diffraction is becoming routine and is a key functionality within the ESRF’s new multi-station automated MASSIF beamline. The X-ray source technologies of both XFELs (see below) and upgraded synchrotron X-ray storage rings are reaching a similar protein crystal sample size range of microns and sub-microns. For a recent overview of SR MX microcrystal diffraction, see Evans *et al.* (2011 ▶). Could electrons be used instead to measure such diffraction data on MX microcrystals and solve these structures? There are renewed developments in this area now, succeeding in electron crystal structure analysis of proteins by molecular replacement, thus taking advantage of the greater scattering efficiency of electrons by matter compared with X-rays (see *e.g.* Nannenga *et al.*, 2014 ▶).

The simple question of how to manipulate ever smaller single-crystal samples leads to quite different technical approaches. One method developed for application at XFELs uses serial delivery of tiny crystals *via* a jet injector system at room temperature and so does away with the classical goniostat of the crystallographer (Chapman *et al.*, 2011 ▶). This serial method leads to a myriad of still diffraction patterns, but with the advent of XFELs new software has been developed (*e.g.* *CRYSTFEL*; White *et al.*, 2012 ▶) to handle these data efficiently and produce complete diffraction data sets. The serial delivery method was also recently proven for the synchrotron using lysozyme as the model system, with over 40 000 crystals exposed and merged to a data set of 2.1 Å resolution (Stellato *et al.*, 2014 ▶). Taking this concept further, recent work has used the ESRF’s ID13 micro/nanofocus diffraction beamline with a sample injector system, again originally developed for use at XFELs but specifically designed for lipidic cubic phase crystals. Another method aims to use many crystals cryo-cooled in one sample support and then raster-scan to locate crystals, collect diffraction data and combine the partial diffraction data sets to form the full data set. A proof-of-concept has been very elegantly performed at the PETRA III facility, where *in vivo* grown microcrystals were exposed on the P14 microfocus beamline, with data from 80 crystals being combined into a 3.0 Å data set (Gati *et al.*, 2014 ▶). All of these developments look ahead to the advent of very low-emittance storage rings with management of the impact of radiation damage.

**Figure 1:**
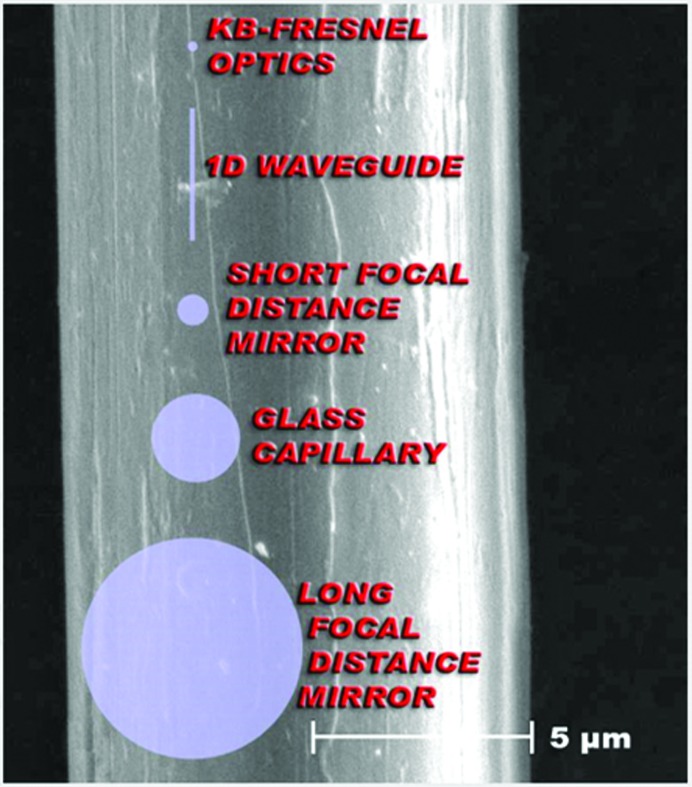
The ESRF beamline ID13 has been and remains at the cutting edge of how small an X-ray microfocus beam can be and undertakes a wide variety of microdiffraction studies including MX. Shown here are the different optical means of providing different sized very small focal spots. (Image reproduced from http://www.esrf.eu/files/live/sites/www/files/UsersAndScience/Experiments/SoftMatter/ID13/poster/esrf_um_2005.jpg with the permission of Dr Christian Riekel of the ESRF.)

Fig. 1 ▶ shows some key developmental steps in producing ever smaller X-ray focal spot sizes and their optics, which are highly relevant to the above discussion. Table 1 ▶ shows the level of X-ray intensities at the sample that are feasible at current national SR facilities for MX (the Canadian Light Source is highlighted as an example).

As samples become smaller, they yield fewer diffraction data before radiation damage renders the sample of no use. Which partial data sets can be realistically combined depends on how similar the samples are. Hierarchical cluster analysis, a method introduced by Wayne Hendrickson and co-workers (Liu *et al.*, 2011 ▶), allows this to be checked and is also now applied at ESRF, as described by Giordano *et al.* (2012 ▶). Much more accurate anomalous signal measurement and greater success in substructure determination can be obtained by merging data from multiple crystals preselected according to the results of cluster analysis. Advances in phasing methods in MX including harnessing weak anomalous signals were discussed at the January 2015 CCP4 Study Weekend (http://www.ccp4.ac.uk/events/CCP4_2015/programme.html) and the proceedings will be published in *Acta Crystallographica Section D*.

### 3.3. Non-conventional X-ray wavelengths   

Most MX beamlines that exist or are under design and construction continue to be optimized for the 2 to 0.8 Å core wavelength range, despite the much wider range of photon wavelengths of around ∼5 to 0.2 Å which has been discussed for use in MX at synchrotron facilities. The core wavelength range covers most of the anomalous absorption edges used, particularly selenium. However, SR MX users are steadily adopting an ever widening practical range of X-ray wavelengths.

**Figure 2:**
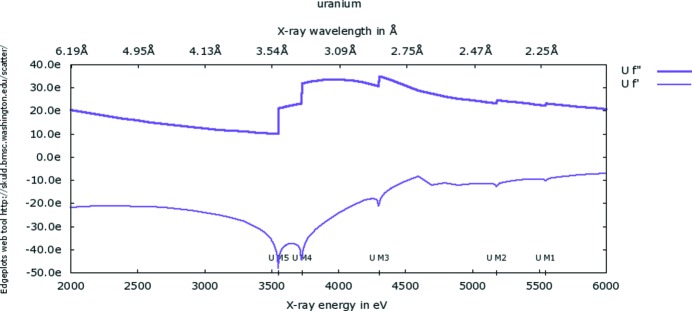
The resonant scattering coefficients *f*′ and *f*′′ for the *M* edges of uranium. Reproduced from the website set up by Dr Ethan Merritt, who is gratefully acknowledged.

Longer wavelengths up to ∼5 Å are under active development for MX at both Diamond and NSLS II. The long-wavelength (1.5–4 Å) Diamond I23 project will be just such a user facility to enhance and optimize the anomalous signals from low atomic number elements. These include sulfur in proteins and/or phosphorus in RNA/DNA crystals, which are needed where protein labelling to introduce anomalous scatterers, such as that involving selenomethionine *via* molecular biology gene expression or heavy-atom chemical derivatization, is not feasible. In addition, the wavelength range of I23 will provide access to the *M* edges of elements, of uranium for example, with larger anomalous signals (Fig. 2 ▶). The idea here is to use the *M* edge *f*′′ maximum values of up to 100 electrons when ‘white lines’ are present (Liu *et al.*, 2001 ▶). The anomalous differences from such an *f*′′ will thus be attractive for measurements of, say, a crystalline large molecular machine complex for MAD structure determination. For such high-angle diffraction data experiments, a large semi-cylindrical vacuum-compatible pixel detector has been designed specifically by DECTRIS (Baden, Switzerland) to capture the diffraction data whilst operating *in vacuo*. To obtain high-quality data, this will be coupled with X-ray computed tomography to obtain the crystal sample shape and volume for an analytical sample absorption correction.

At very short (∼0.5 Å) and ultra-short (∼0.3 Å) wavelengths, high storage-ring energies yield a copious flux output. To date, these have been used for applications such as high-pressure MX, where the restricted aperture of the diamond anvil cell is less of a limitation with shorter wavelengths (Fourme *et al.*, 2011 ▶), and high-energy MAD such as at the holmium *K* edge (see *e.g.* Jakoncic *et al.*, 2006 ▶). Another application of high photon energies involves minimizing radiation damage. Nave & Hill (2005 ▶) have cogently argued that crystals smaller than 10 µm may encounter reduced radiation damage as the photoelectrons can escape from the crystal lattice when using short-wavelength X-rays. Building on this, Finfrock *et al.* (2013 ▶) presented evidence in favour of the use of a 1 µm-wide line focus even for data collection from crystals of around 100 µm, which could also help in the management of radiation damage.

### 3.4. Time-resolved studies   

Time-resolved Laue protein crystallography at the ESRF has opened up a whole new field of sub-nanosecond crystal structure analyses. ‘Fast time-resolved’ biomolecular science examples include carbon monoxy myoglobin and phospho yellow protein (PYP); for a review, see Ren *et al.* (1999 ▶). More complex, but by nature slower, cases include following the enzyme reaction of hydroxymethylbilane synthase in the crystal (Helliwell *et al.*, 1998 ▶). There are only a limited number of such time-resolved studies in the literature, for which there are several reasons. Firstly, crystal lattice interactions can block the necessary structural changes for a given biochemical reaction to proceed. Secondly, crystal size determines the scattering strength of a sample and thereby the required exposure time, which clearly increases as a sample gets smaller. This can obviously be at odds with the intrinsic time resolution required to monitor a given molecular structural change. Different measuring protocols exist which try to surmount this challenge, such as the Hadamard measuring sequence (Yorke *et al.*, 2014 ▶) or the simpler approach of crystal-to-crystal averaging at equivalent time slices (Helliwell *et al.*, 1998 ▶). Meanwhile, XFELs now provide femtosecond duration pulses, typically 10 to ∼50 fs. Their use is attractive for the fastest time-resolved protein crystallography studies. It has been proposed that even single molecules could be studied (Neutze *et al.*, 2000 ▶), which would free us from the crystal lattice restrictions referred to above. A recent comprehensive compilation of XFEL science applied to structural biology, including various time-resolved structural studies, is given by Spence & Chapman (2014 ▶).

### 3.5. Non-traditional and other applications in SR MX   

There are several topics which, in the last few years, have continued to attract attention and development. Room-temperature crystallography is a growing biological crystallography research activity and is a reminder that cryo-derived MX structures do show structural differences. Structural changes occur mostly in the dynamics, as shown by the increased proportion of split-occupancy side-chains at cryo-temperatures. Radiation damage at room temperature used to be the norm for MX in the pre-ribosome crystallography days and damage was mitigated by modest cooling to, typically, 4°C. As mentioned above, serial femtosecond crystallography is generally undertaken at room temperature in any case and ‘the diffraction outruns the damage’. Neutron MX is damage-free and room temperature is routinely employed. However, cryotechniques have other advantages than simply radiation-damage mitigation, namely, under well chosen conditions, improved order and freeze trapping of structural intermediates, provided they are longer lived than the freezing time.

An MX diffraction pattern can have many features which we do not usually seek to explain, namely diffuse scattering, excluding the obvious solvent ring. That this might offer specific information on protein structural dynamics, if it can be teased out from the lattice dynamics, is a long-standing topic. Recently, a short summary of a conference was published (Wall, Adams *et al.*, 2014 ▶), and the book by Peter Moore (Moore, 2012 ▶) nicely summarizes the mathematics of structural and lattice dynamics. Both of these works indicate a renewed determination to use this diffraction information to provide a more complete model and interpretation. In physical crystallography there have been extensive developments, and the so-called full profile analysis is a promising approach for biological crystallography too. The retention of raw diffraction data could provide helpful and much more extensive data set case studies. The dissection of the respective dynamics components, mentioned above, would benefit from the growing trend of measuring MX data at both cryogenic and room temperatures. Interpretation of the diffuse scattering can be achieved *via* molecular dynamics simulations of protein vibrations, which now extend over time periods as long as 1 µs (Wall, van Benschoten *et al.*, 2014 ▶).

The technique of SFX has focused attention on whether micro- and nanocrystals are better quality (typically, a lower mosaicity is referred to) than ‘routine-sized’ crystals (see, for example, the volume edited by Spence & Chapman, 2014 ▶). The techniques described in detail in the book by Chayen *et al.* (2011 ▶) can be applied to the systematic evaluation of crystal perfection as a function of sample size. In chemical microcrystallography, Andrews *et al.* (1988 ▶) stated that, where crystals have a high mosaicity, they will not grow larger. The corollary of this is that if microcrystals have a low mosaicity then they can grow larger. Certainly, at ICCBM15 (15th International Conference on the Crystallization of Biological Macromolecules, 17–20 September 2014, Hamburg, Germany) there was a very healthy interest in growing large enough crystals for neutron MX where research into the structure and function of a molecule warrants it. It is therefore very important that we do not give up on knowing how to grow larger crystals, nor on acquiring further knowledge of growing them.

## 4. Spin offs from SR MX   

### 4.1. SR MX leads the way for commercial industry access   

Industrial use of the synchrotron research infrastructure is a core mission of most such facilities. At the SRS Daresbury, commercial access to MX was the largest share of the Daresbury Analytical Research Technical Service (DARTS) (Maclean *et al.*, 2006 ▶). Nowadays, most synchrotrons have an industry or business development office, managing and developing links with industry and creating economic value and impact from industrial access [see, for example, Cutler (2014 ▶), Shotton *et al.* (2014 ▶) and Mitchell *et al.* (2011 ▶)]. Drug discovery is still a significant income generator for all facilities, yielding millions of euros per year in industrially derived income flowing into the facilities.

Industry requirements have driven aspects of SR MX automation and, in particular, the development of metadata models and database systems to track data collections and processed results and to make all the information available *via* a secure web link for remote access and download of results. Malbet-Monaco *et al.* (2013 ▶) explored the impact of this ‘reverse spin-off’ benefit back to the facilities. Industrial needs have also driven the development of ‘mail-in’ crystallography, with the services described in *Nature* in 2003 (Schmidt, 2003 ▶) for the NSLS and the ESRF being prominent examples.

Spin-off data-collection service companies have been created, such as Expose GmbH from the SLS which provides access to SR MX facilities. Other businesses, all using synchrotron access, deliver rapid structure solution and structural biology services, such as Saromics (Sweden) which delivers kinase structural data, Bio-Xtal (France), Shamrock Structures (US) and VivaBiotech (China), amongst many others. The wholesale uptake and acceptance of structural biology, and thus of SR MX, for drug discovery by the pharma­ceutical industry, and the maturation of automation, have led to such enterprises having tenable business cases. High-throughput SR MX has assisted in the viability of the fragment-based drug discovery industry (Badger, 2012 ▶), which relies on a steady stream of biophysical and structural data, in particular from X-ray crystallography [see Chilingaryan *et al.* (2012 ▶) for a recent review].

SR facilities are now seeking to develop in a similar manner to other industry sectors, with national governments and the EU seeing industrial activity as one of the metrics of a facility’s success (whether synchrotrons, neutron sources, lasers or other research infrastructure).

The exploitation of SR MX facilities for economic value is achieved not only *via* industrial use and the occasional patent or university spin-off based on structural data, but also in the transfer of technology and knowledge to instrumentation suppliers who then sell the MX instrumentation worldwide. Examples of this include diffractometers, sample-changer robots and sample pins.

### 4.2. Public understanding of science, engineering and technology   

SR facilities and their research user programmes attract considerable public and political interest (see *e.g.* http://www.stfc.ac.uk/3388.aspx). Government funding agencies closely monitor the ‘high-impact’ publications that research produces. MX is a principal contributor to demonstrating success by these metrics. A clearly helpful factor over the years has been the award of Nobel Prizes for structural biology for which the research required SR MX. Thus, there was the Nobel Prize to John Walker (shared) for F1ATPase (SRS); to Rod McKinnon for the potassium ion channel (CHESS, USA); to Roger Kornberg for RNA polymerase (SSRL); to Venki Ramakrishnan, Tom Steitz and Ada Yonath for ribosomal structure studies (featuring many SR facilities and especially involving the NSLS, APS and ESRF); and to Brian Kobilka (shared) for GPCRs (APS and ESRF). These and other crystallography-derived Nobel Prizes are described in the book by Olovsson *et al.* (2015 ▶), including the key research articles.

### 4.3. An evolving offer to SR MX users   

The view of central SR facilities and users towards MX is evolving. For structural biology users, the role of the synchrotron now extends beyond ‘just’ data provision *via* X-ray crystallography. Protein crystallographers have adopted bioSAXS (biological small-angle X-ray scattering) in recent years. An interesting initiative for a combined MX and bioSAXS beamline at the ALS in Berkeley is described by Classen *et al.* (2013 ▶). The rise of bioSAXS has included automation of the beamline hardware and software pipeline to allow efficient data validation and modelling.

Combined X-ray and neutron structural biology studies [see *e.g.* the book by Svergun *et al.* (2013 ▶)] are being facilitated at science campuses like that of ESRF and the ILL, with the neutron work making use of deuteration and contrast matching for the study of multi-protein or DNA/RNA/protein complexes. Central facility sites are also actively creating joint services for structural biology, combining X-rays and neutrons with other techniques such as NMR and electron microscopy (EM). At the Rutherford Appleton Laboratory (RAL), which hosts Diamond and ISIS, new state-of-the-art cryo-EM facilities will be treated like a beamline, with users able to request this technique in addition to SR MX. Indeed, most major research infrastructure sites in Europe have or are developing multidisciplinary centres, like the Partnership for Structural Biology (Grenoble), the first of its type, the Centre for Structural Systems Biology (Hamburg), and the Membrane Protein Laboratory and Oxford Protein Production Facility (RAL). In the USA, the APS is building its Advanced Protein Crystallization Facility, which will allow the production, characterization and crystallization of proteins. All these, often multi-facility/multi-laboratory, partnerships stretch facility impact beyond the supply of X-rays and/or neutrons to helping users to prepare proteins, perform quality control and crystallize them, refine conditions, and use complementary structural analysis techniques to provide an overall structural picture of challenging macromolecular systems. Crystallization and the use of SR are closely intertwined (Chayen *et al.*, 2010 ▶).

Critical information complementary to SR MX is provided by neutron MX, which is becoming a routine technique for the community. This is largely thanks to the provision of deuterated macromolecules as part of neutron facility programmes, and enhanced neutron beamline performance to reduce data-collection times and enhance diffraction resolution limits. A major review and summary of the field of neutron MX has been published by Blakeley (2009 ▶). This includes showing how the limits of high molecular weight and smaller crystal sample, as well as the speed of measurement, have been significantly improved with the neutron Laue method. The increased background noise that inevitably comes from using the broader spectral bandpass of the Laue method has had only a marginal, if any, effect on the diffraction resolution limit achieved. The total elapsed time for taking a data set measurement has also improved significantly. A future development at the ILL is the CYCLOPS (cylindrical CCD Laue octagonal photo scintillator) single-crystal diffractometer. This has a set of area detectors and will further speed up single-crystal diffraction intensity measurements with neutrons. Recently, the ILL beamline D19, with its refurbished detector, has allowed very high-resolution neutron structures to be determined (Cuypers *et al.*, 2013 ▶). There are also high-megawatt spallation neutron source MX instruments at the USA’s SNS and Japan’s JPARC. The steady growth of the field of neutron MX is described in the book by Niimura & Podjarny (2011 ▶).

## 5. Availability of raw diffraction images at SR facilities   

Retaining raw diffraction data has become an increasingly debated topic in recent years. The Australian synchrotron is leading on data archival with its Store.Synchrotron data storage service. As well as diffraction image data archiving, it also supports users in their publications with linking to raw data sets *via* DOI registrations and, finally, the release of data sets for public analysis – something that, in the neutron community, the ILL is doing as well. There are also fine examples like Diamond that has so far retained all of its measured data. The ESRF has published a summary of its views on the era of Big Data at SR facilities in general and the challenges involved today, as exemplified by ESRF itself (ESRF, 2013 ▶). The imgCIF dictionaries continue to be developed in a way that will facilitate interoperability with NeXus/HDF5 workflows at synchrotron radiation facilities, and imgCIF/CBF is now supported as an image format by all the major vendors.

The challenges of and possibilities for raw diffraction MX data are discussed in several recent articles in *Acta Crystallographica Section D* [see Terwilliger (2014 ▶), Kroon-Batenburg & Helliwell (2014 ▶), Guss & McMahon (2014 ▶) and Bricogne & Terwilliger (2014 ▶)].

## 6. Summary and outlook   

The excellent SR infrastructure now established for MX spans high-brilliance SR sources of front-line capabilities at national and international level. It is amazing that time-resolved protein crystallography is undertaken at the nanosecond time scale using Laue diffraction at, for example, the ESRF and the APS, and now at the femtosecond time scale at the LCLS. The choice of photon wavelength is still widening; the usage of longer wavelengths up to ∼5 Å is set to become a regular feature of our data-collection repertoire. Special experiments such as high-pressure protein crystallography have been routinely using wavelengths as short as 0.3 Å (Fourme *et al.*, 2011 ▶), and even wavelengths down to 0.2 Å have been investigated for MX (Jakoncic *et al.*, 2006 ▶).

The speed of routine MX data collection, facilitated by such brilliant X-ray beams and the new generation of pixel detectors, is now minutes per data set, benefitting the entire academic and industrial user community for high-throughput fragment-based drug discovery or simply for selecting the best diffracting sample out of many. One of us (JRH) delivered an IUCr Montreal Keynote Lecture (available as supporting information to this article) within which the question, ‘How did we arrive at such excellence as modern beamlines now offer MX?’ was posed. That lecture charted our progress from the situation in 1979 with the SRS, which had a horizontal source size of ∼14 mm with which one had to plan an instrument (SRS 7.2) to focus down to an X-ray beam of 0.3 mm, up to today, where we routinely seek focused X-ray beams of 5 µm, and where we are also now seeing the first X-ray diffraction MX experiments at the sub-micron crystal sample size level. Whilst everything in 1979 was done manually, remote access and the routine use of robots are now the mode of data collection for SR MX users. Diffraction data volumes today are already challenging, and the projected volumes expected from the ESRF Upgrade and the similar facility evolutions being programmed worldwide take us from Big Data to ‘Massive Data’.

SR MX is developing improved and new methodologies, including combined approaches with neutrons, EM and/or NMR. It remains to be seen how long before (and not if) these new techniques join the automated SR MX we know today as a component of the biology tool box that academia and industry use routinely.

## Supplementary Material

Summary of abbreviations. DOI: 10.1107/S205225251402795X/fs5088sup1.pdf

Montreal talk. DOI: 10.1107/S205225251402795X/fs5088sup2.pdf

Montreal talk. DOI: 10.1107/S205225251402795X/fs5088sup3.pptx

## Figures and Tables

**Table 1:** Illustrative operational parameters for a beamline at the Canadian Light Source One example of the cutting edge of current national SR MX facilities is the Canadian Light Source (CLS), which has a beamline for conventional MX crystals with a typical focal spot of 140 40m. 08ID-1 is an automated beamline for MX experiments at the CLS (Fodje *et al.*, 2014 ▶; Grochulski *et al.*, 2014 ▶). This will typically be supplemented by a microfocus complementary performance beamline, the performance details of which are also described in this table. The authors are grateful to Pawel Grochulski of the Canadian Light Source for permission to reproduce these details here.

	CLS 08ID-1	CLS 08ID-1 proposed
Spectral range	6.018.0keV/(2.10.7)	5.022.0keV/(2.50.6)
Energy bandwidth (*E*/*E*) Si(111) at 12keV	1.5 10^4^	1.5 10^4^
Measured focal size at 12keV (full width at half-maximum) (m m)	150 (H) 30 (V)	50 (H) 5 (V)
Flux on the sample at 12keV (250mA) (photonss^1^) (from the sixth harmonic of the insertion device)	5 10^12^	>10^13^
	1 10^12^ (50m)	>10^13^ (50m)
	7 10^11^ (20m)	>10^11^ (5m)
	2 10^10^ (5m)	
Typical beam size (m)	50	20
Beam crossfire at the sample at 12keV (FWHM) (mrad mrad)	0.9 (H) 0.2 (V)	1.8 (H) 0.5 (V) (less with pinholes)
