# Supplementary material for: Synchrotron radiation macromolecular crystallography: science and spin-offs
Source: IUCrJ. 2015 Feb 3;2(Pt 2):283–91. doi: 10.1107/S205225251402795X (PMC4392420; doi:10.1107/S205225251402795X)
Supplement: Supplementary file 1 [file m-02-00283-sup1.pdf]

IUCrJ

Volume 2 (2015)

Supporting information for article:

Synchrotron radiation macromolecular crystallography: our science and spin offs

John R. Helliwell and Edward P. Mitchell

Summary of abbreviations used

APS: Advanced Photon Source

CLS: Canadian Light Source

DLS: Diamond Light Source

ESRF: European Synchrotron Radiation Facility, Grenoble, France

ILL: Institut Laue Langevin, Grenoble, France

MASSIF: Massively Automated Sample Selection Integrated Facility

MAX IV: Laboratory A Swedish national laboratory at Lund University, which supports three distinct research areas: accelerator physics, research based on the use of synchrotron radiation, and nuclear physics

PDB: Protein Data Bank

PETRA III: The new high-brilliance third-generation synchrotron radiation source at DESY

RAL: The Rutherford Appleton Laboratory, UK

RIKEN: An Independent Administrative Institution whose formal name in Japanese is Rikagaku Kenkyūsho (理化学研究所[?]).

SERCAT: The South East Region (of the USA) Collaborative Access Team of the APS

SOLEIL: An acronym for Optimized Source of LURE* Intermediary Energy Light. It is a research centre located on the Plateau de Saclay in Saint Aubin, Essonne, France

SPring-8: Super Photon Ring – 8 GeV

SRS: Synchrotron Radiation Source Daresbury Laboratory, UK (1980-2008)

SSRL: Stanford Synchrotron Radiation Laboratory, USA
