# Supplementary material for: Synchrotron radiation macromolecular crystallography: science and spin-offs
Source: IUCrJ. 2015 Feb 3;2(Pt 2):283–91. doi: 10.1107/S205225251402795X (PMC4392420; doi:10.1107/S205225251402795X)
Supplement: Supplementary file 2 [file m-02-00283-sup2.pdf]

IUCrJ

Volume 2 (2015)

Supporting information for article:

Synchrotron radiation macromolecular crystallography: our science and spin offs

John R. Helliwell and Edward P. Mitchell

CONGRÈS ET ASSEMBLÉE GÉNÉRALE
DE L'UNION INTERNATIONALE DE CRISTALLOGRAPHIE

IUCr Montreal Congress Keynote Lecture August 6th 2014

Synchrotron radiation macromolecular crystallography: our science and some spin offs

John R Helliwell

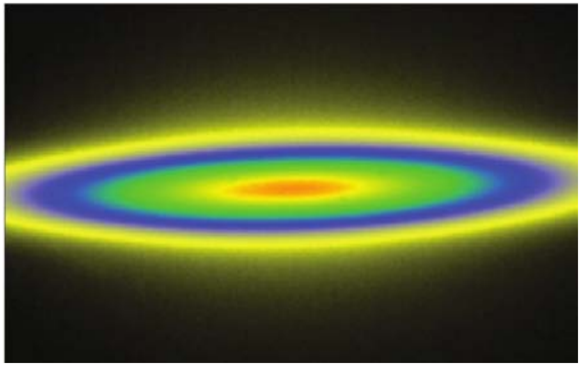

CONGRESS AND GENERAL ASSEMBLY
OF THE INTERNATIONAL UNION OF CRYSTALLOGRAPHY

Contents

- *Canadian Light Source MX*
- *Historical aspects: obviously a somewhat personal selection*
- *Our science applications*
- *Current & future beamline developments*
- *New X-ray sources*
  - *the national 3rd generation sources;*
  - *the ultimate storage rings;*
  - *the X-ray lasers*
- *Some spin offs*
  - *SR commercial usage; SR chemical crystallography ; neutron MX;*
  - *an example of the Public impact of SR and structural science*

Canadian Light Source MX

A frontier national infra structure

Canadian Light Source
Centre canadien de rayonnement synchrotron

The Canadian MX Facility has ID and BM beamlines & supports over 60 labs.

	CMCF-ID Current	CMCF-ID Proposed
Spectral range (keV)/(Å)	6.0 - 18.0 /(2.1 - 0.7)	5.0 - 22.0 /(2.5 - 0.6)
ΔE/E Si(111) @12 keV	1.5×10^{-4}	1.5×10^{-4}
Focal size @ 12 keV (FWHM) (μm x μm)	150 (H) x 30 (V)	50 (H) x 5 (V)
Flux on the sample @ 12 keV (photons/s)	5×10^{12}<br>1×10^{12} (50 μm)<br>2×10^{10} (5 μm)	$>10^{13}$<br>$>10^{13}$ (50 μm)<br>$>10^{11}$ (5 μm)
Typical beam size (μm)	50	20

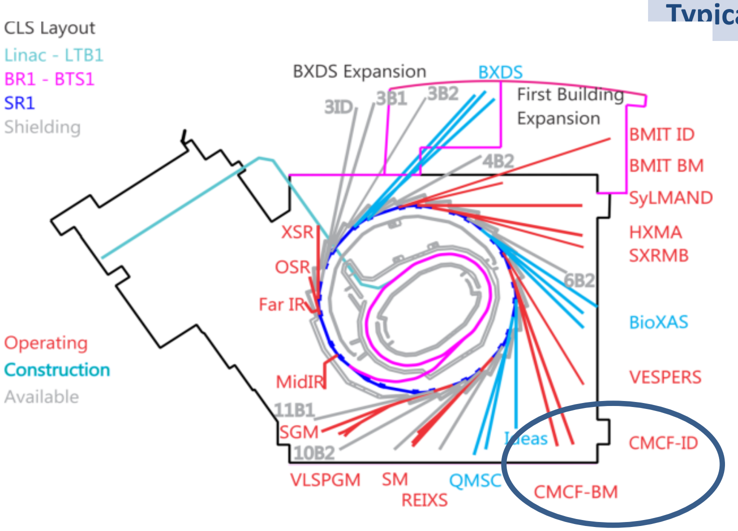

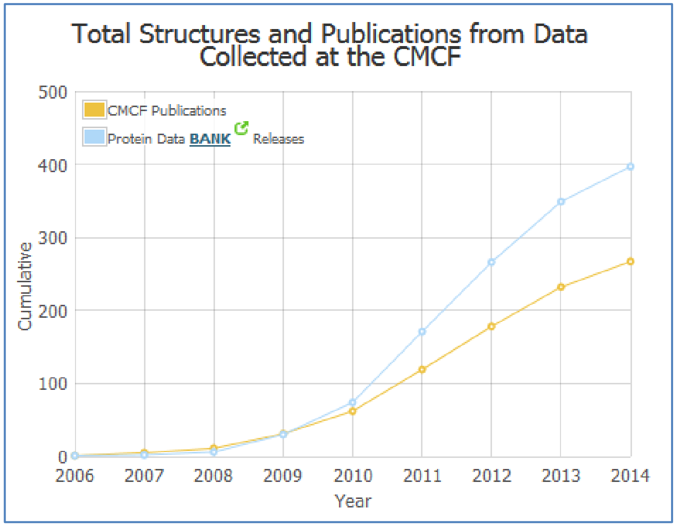

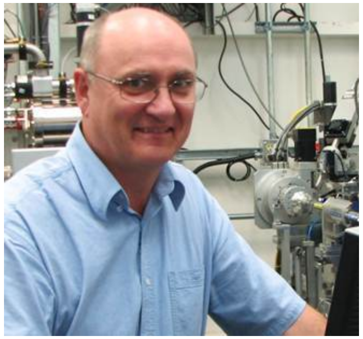

CLS MX Leader
Pawel Grochulski

JRH visit to CLS 2002

http://cmcf.lightsource.ca

How did we arrive at this excellence?:

Historical aspects (& obviously a somewhat personal selection)

Challenges in the mid 1970s

- Weak diffraction and long exposure times in the home lab for MX
- Solving the crystallographic phase problem was haphazard
- I heard ***Mössbauer*** in his lecture at RAL suggest the use of nuclear anomalous dispersion
- The IUCr book Anomalous scattering was just published in 1975
- It was a fertile time for a physicist like me entering protein crystallography (although at my interview one person said “don’t bother, all the methods are fine as they are”)
- First MX expts at SSRL mid 1970s were led by ***Keith Hodgson***; I found the preprint* on this very exciting!

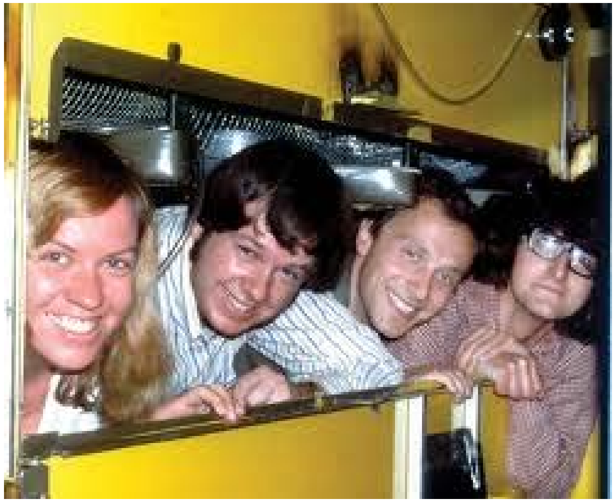

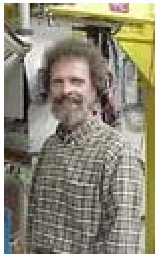

* Phillips, J. C., Wlodawer, A., Yevitz, M. M. and Hodgson, K. O. (1976) PNAS, USA 73, 128-132; their ref 1:-

1. Rosenbaum, G., Holmes, K. C. & Witz, J. (1971) *Nature* 230, 434–437.

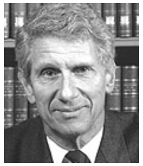

SRS PX 7.2 on the first dedicated SR X-ray source:
also ensuring tuneable λ (1.2 to 2.6 Å) and SRS PX9.6 (0.5 to 1.38Å)

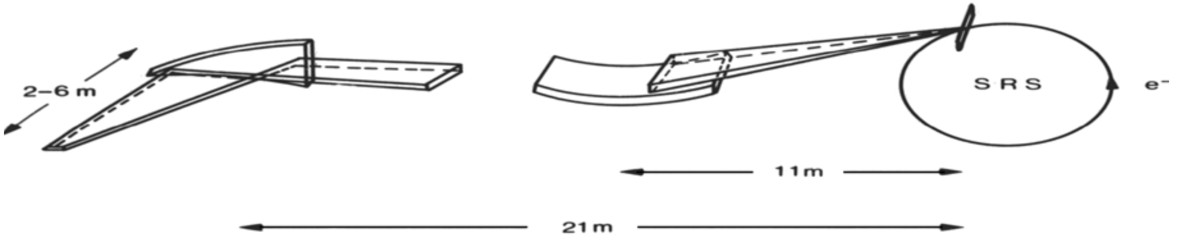

Helliwell, Greenhough, Carr, Rule, Moore, Thompson & Worgan J Phys E 1982 SRS 7.2

Helliwell et al NIM (1986) <u>A246</u>, 617-623

Inspired by Keith Hodgson et al 1976 and Gerd Rosenbaum and Ken Holmes 1971

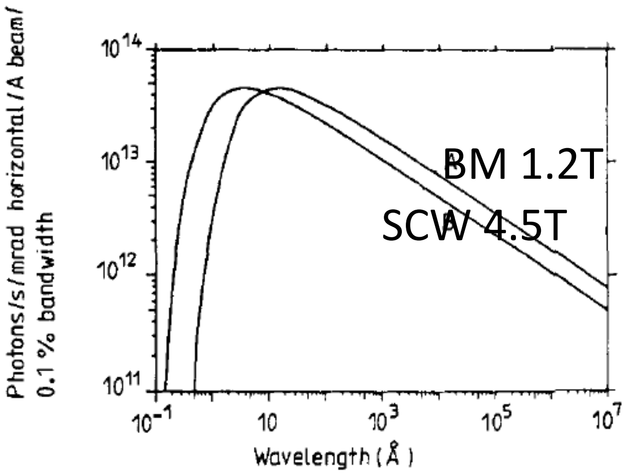

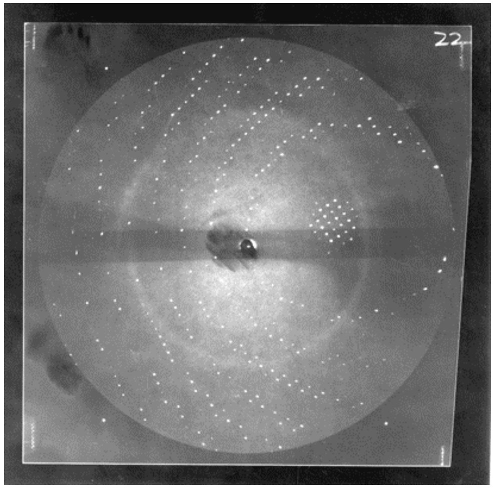

Eg λ=2.6 Å

I had explored gradually using wavelengths:- 0.6Å, 0.9Å, 1.488Å, 1.739Å, 1.86Å , 2.0Å, 2.6 Å.

Helliwell (1984) Reports on Progress in Physics 47, 1403-1497

Even though the initial SRS was not that well suited to crystallography, especially with its horizontal source size of 14mm, initially I could get 20 times the home Lab intensity, as well as being fully tuneable. The addition of the vertically focussing mirror brought us up to x100 times.

So we got our first SRS PX users, UK and international, and exciting results>>>

The Mn K edge was accessible on SRS 7.2 at 1.896 Å

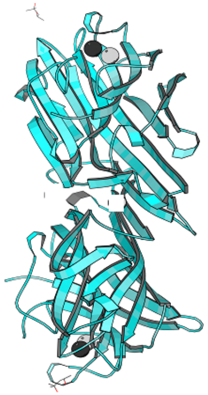

Pea lectin

Acta. Cryst. (1985) B41, pp. 336-341.

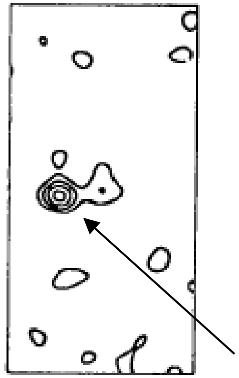

Manganese ion (strong anomalous peak)

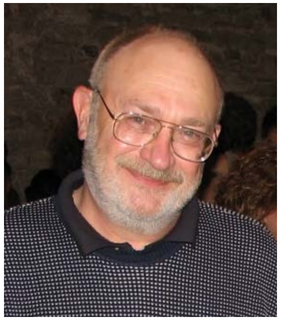

Howard Einspahr

Purine nucleoside phosphorylase; the challenge of 80% solvent content in the crystal overcome by high intensity on SRS 7.2

Drugs by Design

SCIENTIFIC AMERICAN *December 1993*

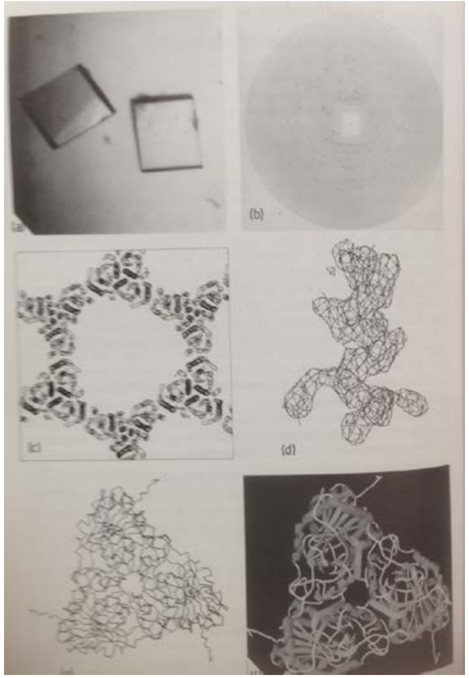

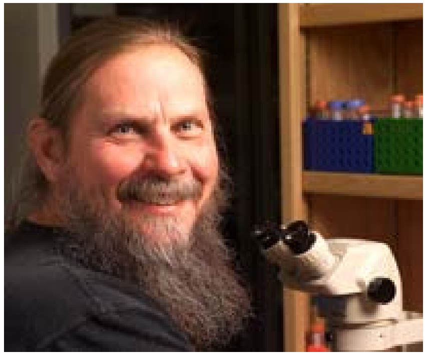

Steve Ealick

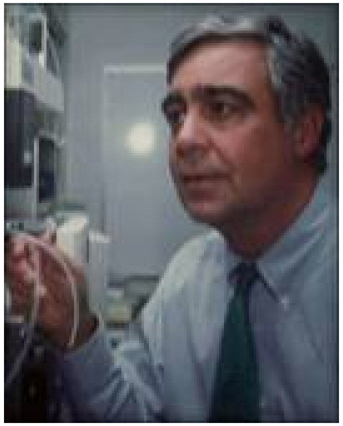

Charlie Bugg; UAB Prof & CEO Biocryst Pharmaceuticals

Alabama

Virus structures; a key step involved the methods…

J. Appl. Cryst. (1983). **16**, 629–636

Oscillation Photography of Radiation-Sensitive Crystals using a Synchrotron Source

By Michael G. Rossmann and John W. Erickson
Department of Biological Sciences, Purdue University, West Lafayette, Indiana 47907, USA

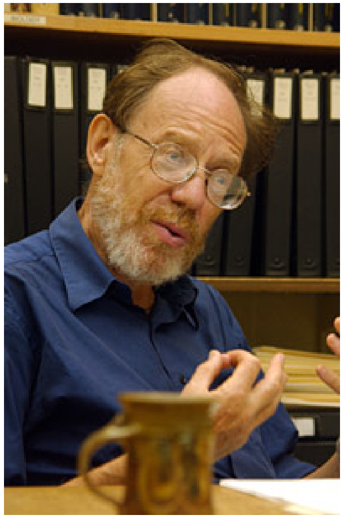

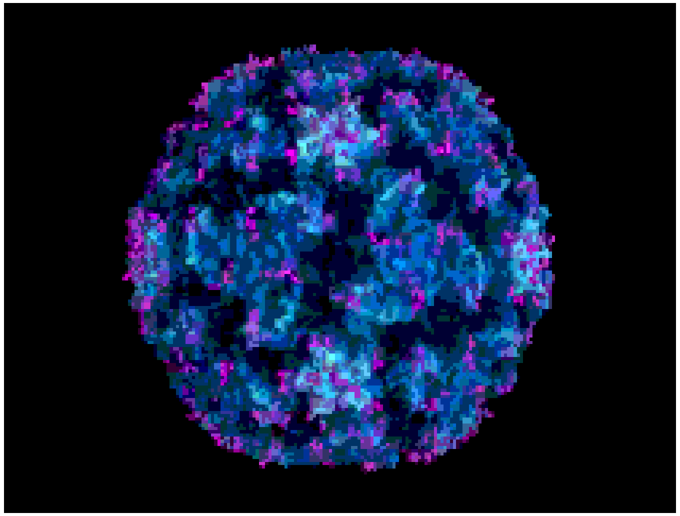

SRS Station 7.2 and Hamburg 'DESY':-

We wish to thank Drs Hans Bartunik and Klaus Bartels at DESY in Hamburg, Germany and Dr John Helliwell at the SRS in Daresbury, England for much help and encouragement in the difficult task of X-ray diffraction data collection of the R14 rhinovirus crystals.

Prof M G Rossmann, USA;

1985 human rhinovirus-14, HRV-14; the rhinovirus structure was finally based on diffraction data measured at ***MacCHESS.***

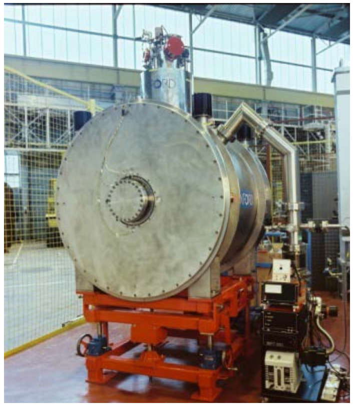

Virus crystallography at SRS 9.6

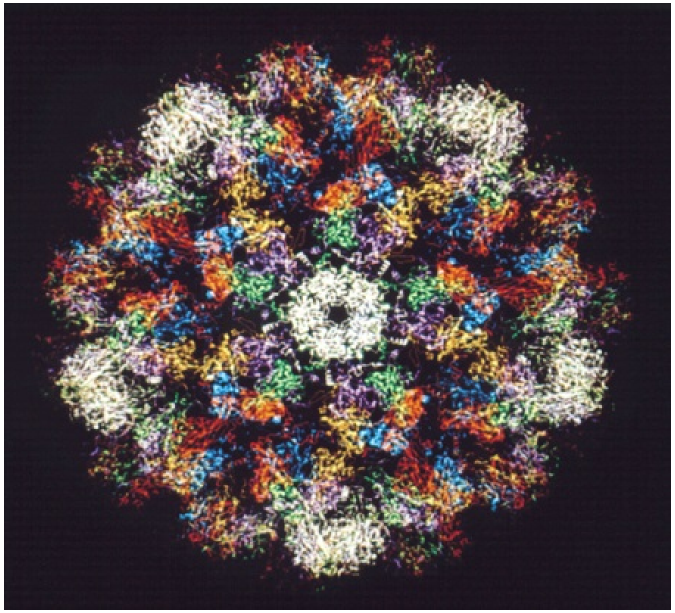

SV 40 virion

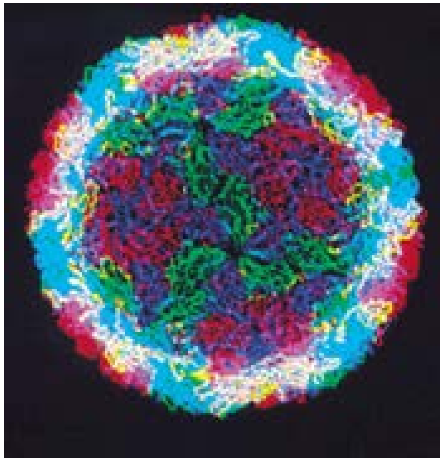

FMDV

BBC NEWS

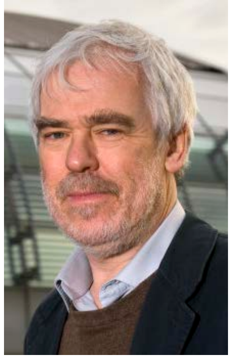

David Stuart

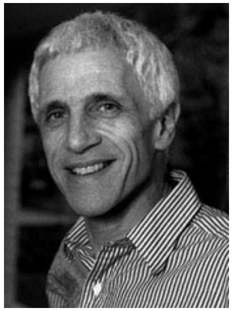

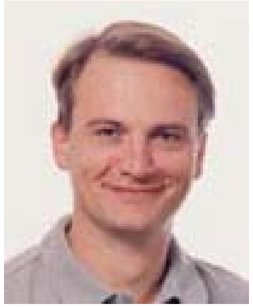

Steve Harrison & Bob Liddington et al Harvard using data measured at SRS 9.6 and CHESS

Vetenskapsrådet
Sweden

SRS 9.5; focussed & rapidly tuneable X-ray beam & an IP

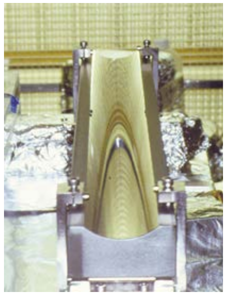

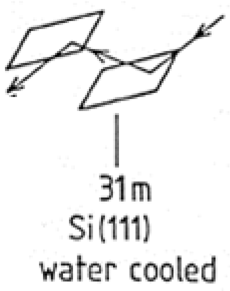

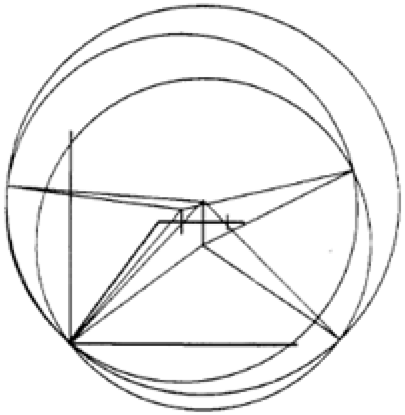

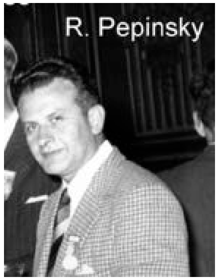

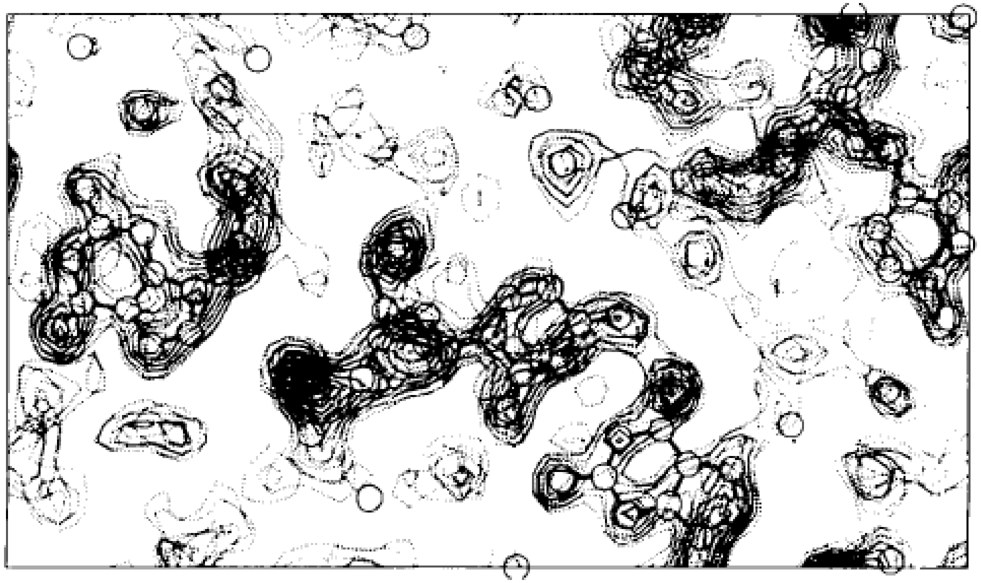

Case 5.7

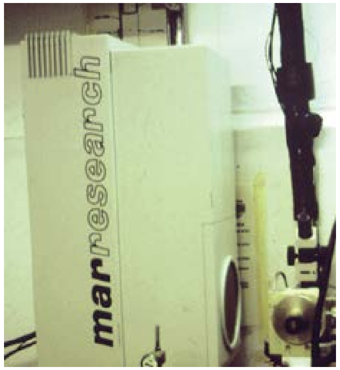

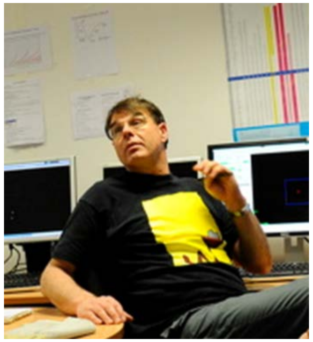

Andy Thompson

Two wavelength phasing of a brominated oligonucleotide
Peterson, Harrop, McSweeney, Leonard, Thompson, Hunter and Helliwell
1996 JSR 3, 24-34

Helliwell 1979 based on **Okaya and Pepinsky 1956**

But we were short of intensity on SRS 9.5; this led naturally on to ESRF

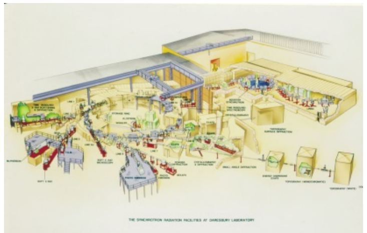

*Let's take **an overview** for a moment*

- 1981 Daresbury SRS; the first dedicated '2nd generation' SR source; we attracted some of the very best users in the World
- Mid-1980s PEP as a new ring concept emerges from SSRL
- 1987 ESRF Foundation Phase Report 'Red Book'; this showed colossal increases in X-ray intensities from a new source, the X-ray undulator. Would the samples stand it?

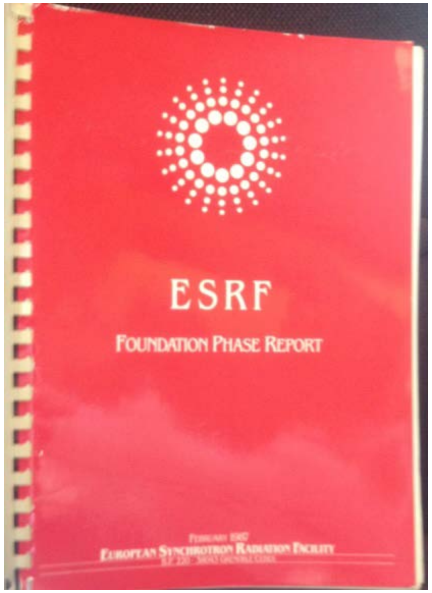

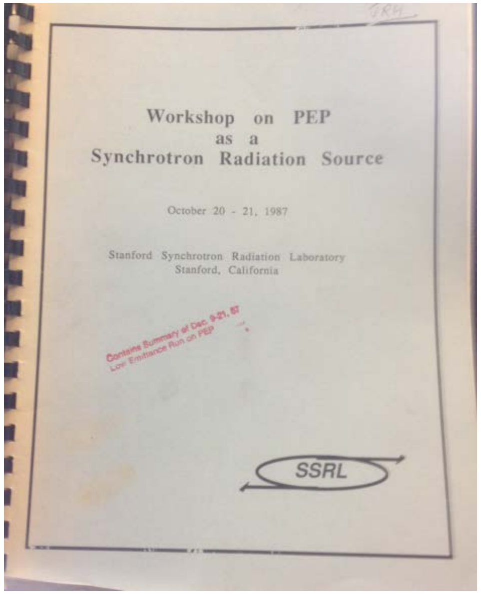

ESRF greatly improved emittance over SRS

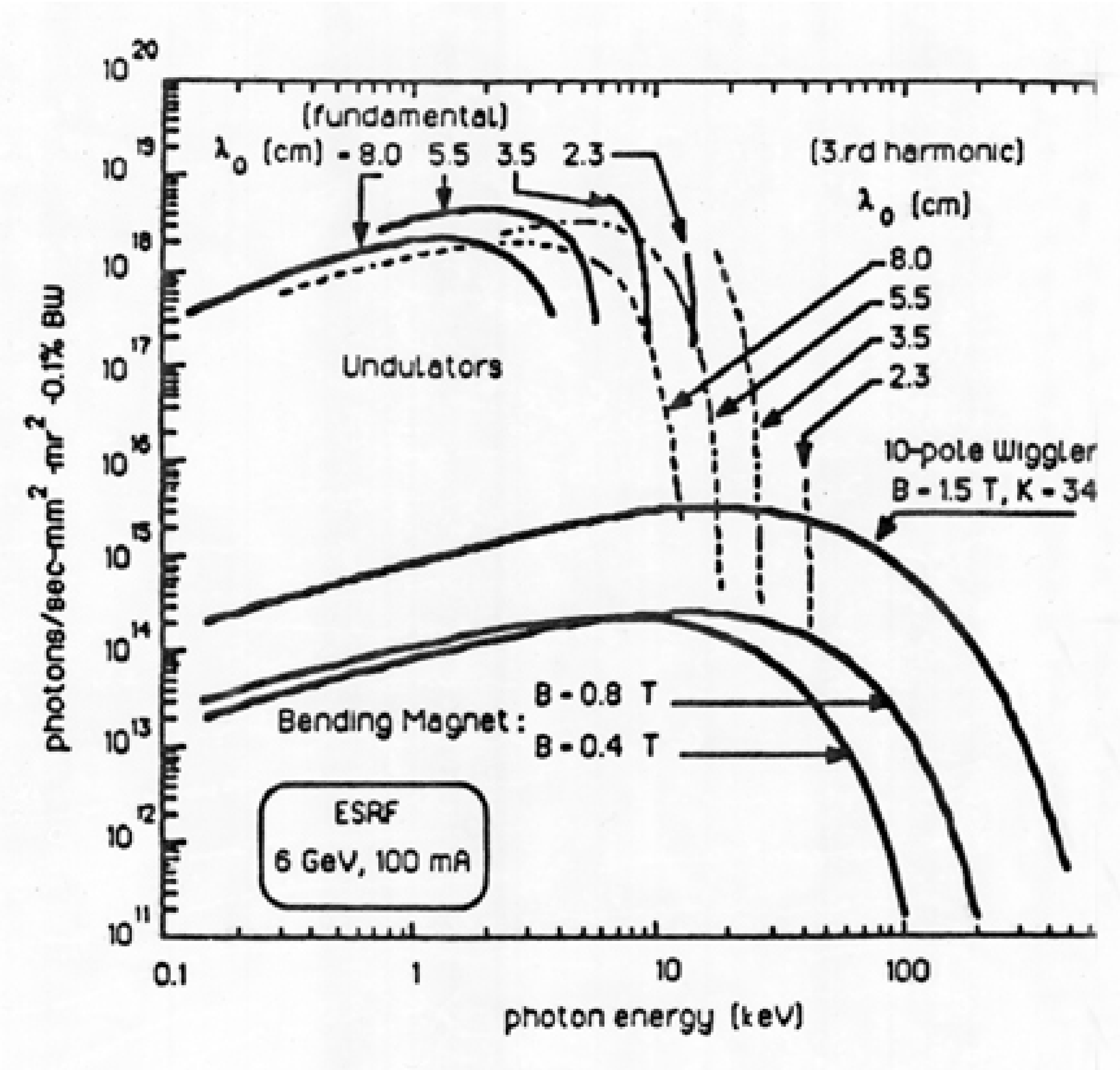

These curves were at the time of the ESRF Foundation and have improved considerably

Biological microcrystallography with 'New Rings'

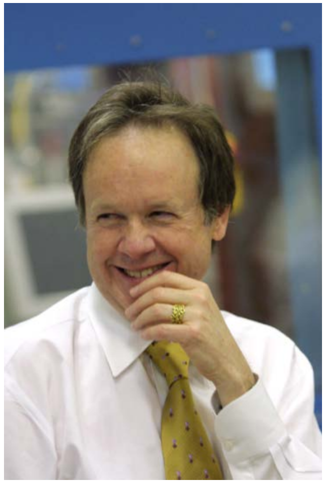

Keith Hodgson

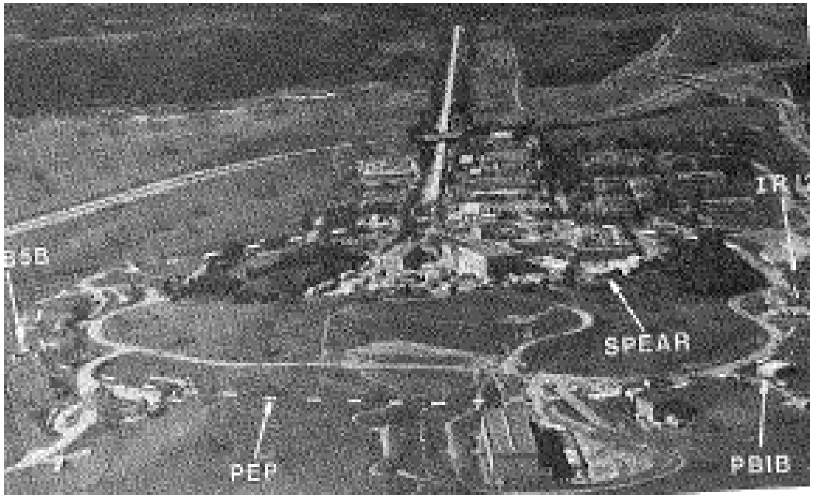

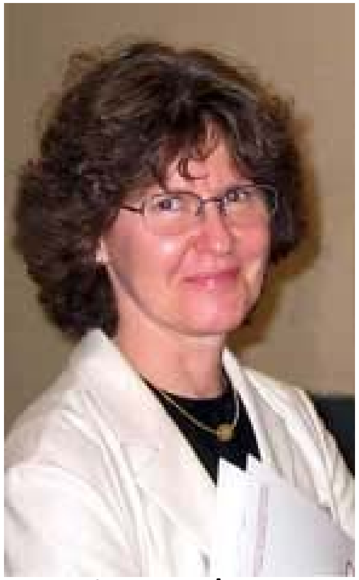

Britt Hedman

Stanford's New Rings Workshops in the early to mid 1980s

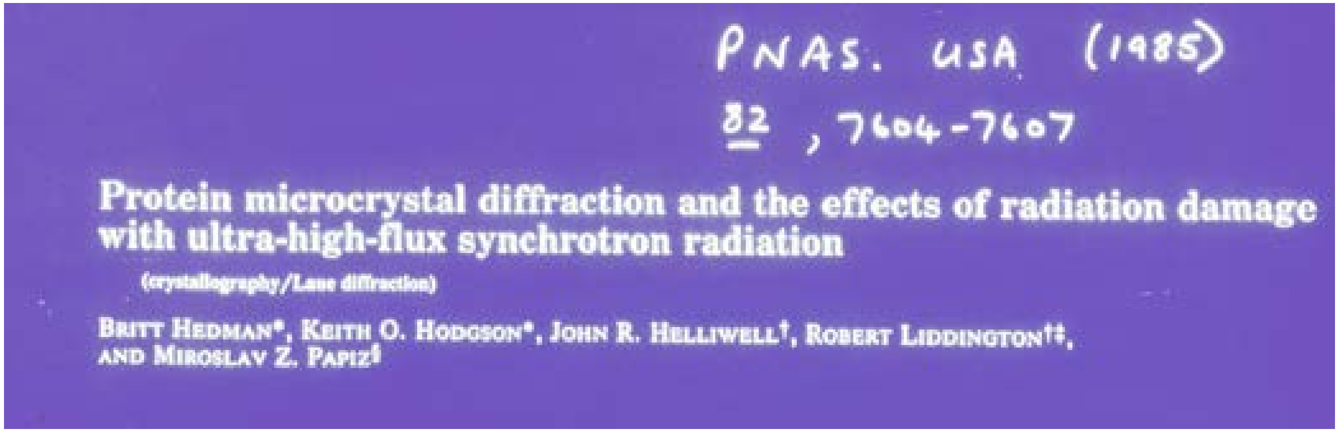
PNAS. USA (1985)
82, 7604-7607

Protein microcrystal diffraction and the effects of radiation damage with ultra-high-flux synchrotron radiation

(crystallography/Laue diffraction)

Britt Hedman*, Keith O. Hodgson*, John R. Helliwell†, Robert Liddington†‡, and Miroslav Z. Papiz§

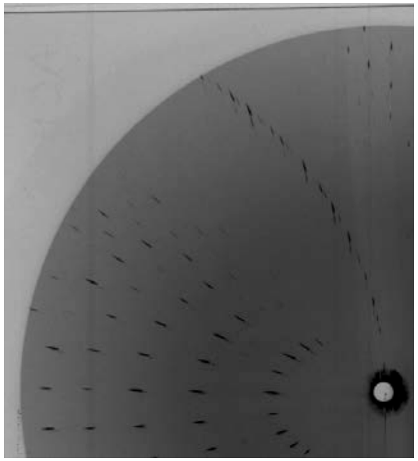

SRS wiggler white X-rays test proposed monochromatic undulator ESRF X-ray intensities

ESRF planning by ESRP required simulation of the effect on protein crystals; beam heating and radiation damage and initiate practical *risk assessment* experiments

J.R. Helliwell and R. Fourme 'The ESRF as a facility for protein crystallography: A report and design study'. ESRP Report IRI-4/83(1983), pp. 1-36.

A beam heating risk management strategy was offered (*Cryo has superceded this!)*:-

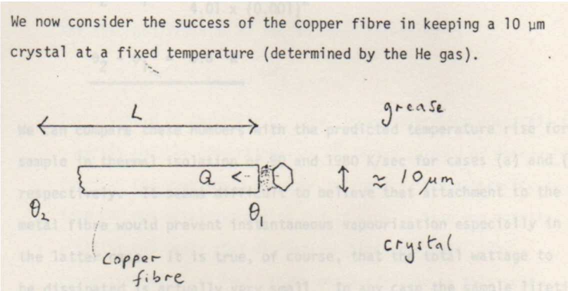
We now consider the success of the copper fibre in keeping a 10 μm crystal at a fixed temperature (determined by the He gas).

20Ks^{-1} adiabatic!

J.R. Helliwell / Protein crystal perfection and nature of radiation damage

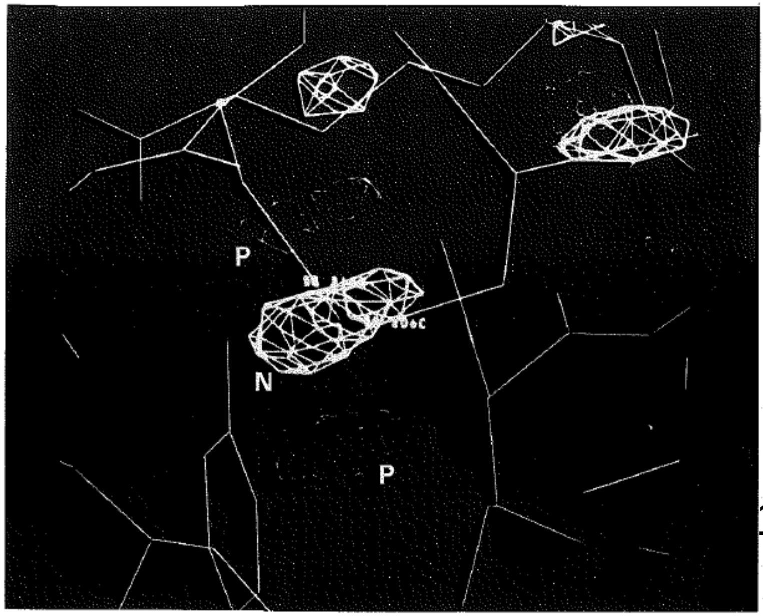

P=positive
N=negative
electron density

10MRad = 0.1MGy

(Fdamaged – F undamaged) expiα$_{calc}$

ie a 20 + 2 Mrad data set at Stanford – a 0+ 7.5Mrad of X-ray dose data set at Daresbury

Splitting of disulphide bridges in proteins due to X-rays; based on a suggestion of Greg Petsko

Our science applications

The statistics: SR MX dominates the X-ray PDB depositions

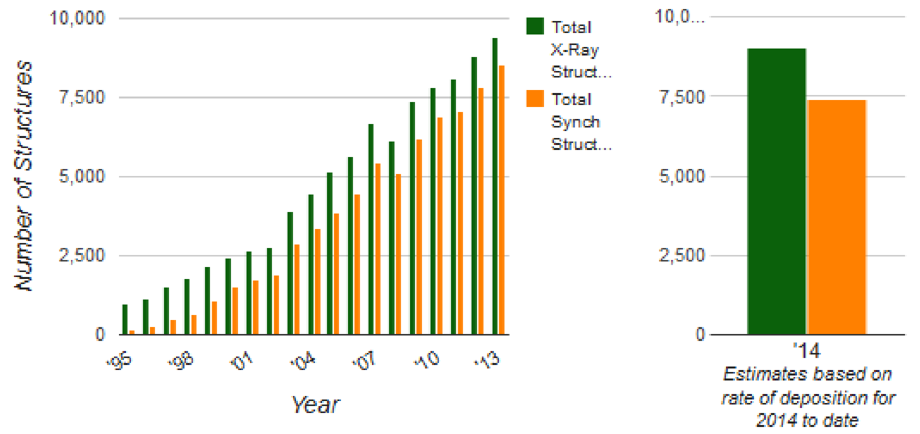

Regional distribution of PDB structures determined at synchrotron facilities through 2013

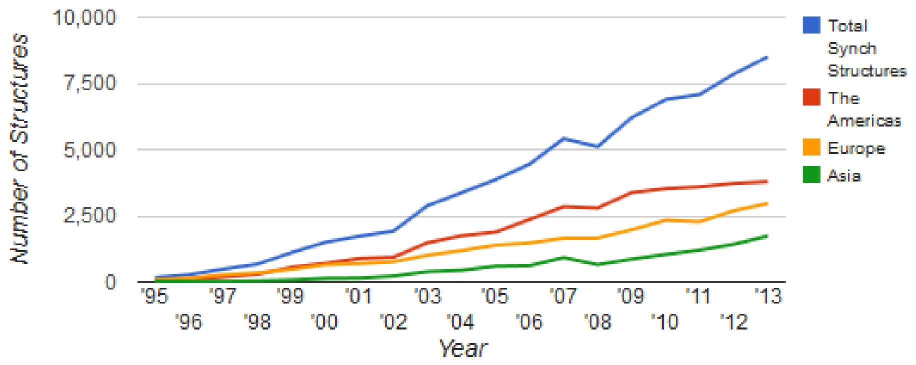

http://biosync.sbkb.org/index.jsp#

Date of last update: 2014-07-01

MAD/SAD (inc density modification) methods and molecular replacement, software (CCP4, Phenix, SHELX, Il Milione ….), Cryo, Automation, gene expression are all very relevant to the rate of MX structure determinations ie some SR specific aspects and some not

Seleno-methionine hydroxymethylbilane synthase fluorescence scan measured at SRS 9.5

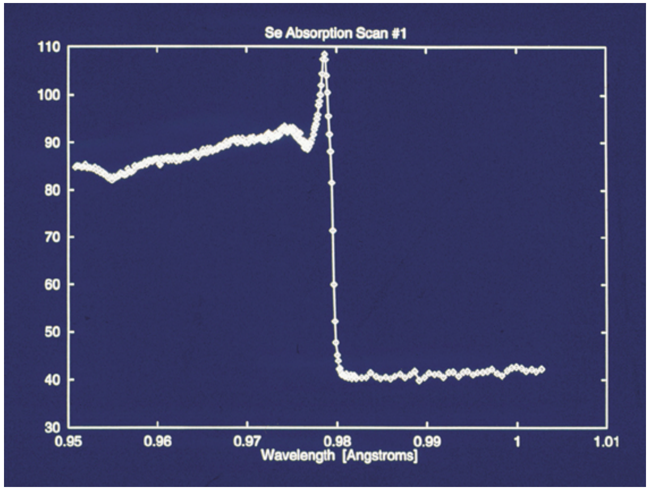

Anom diff Patterson maps

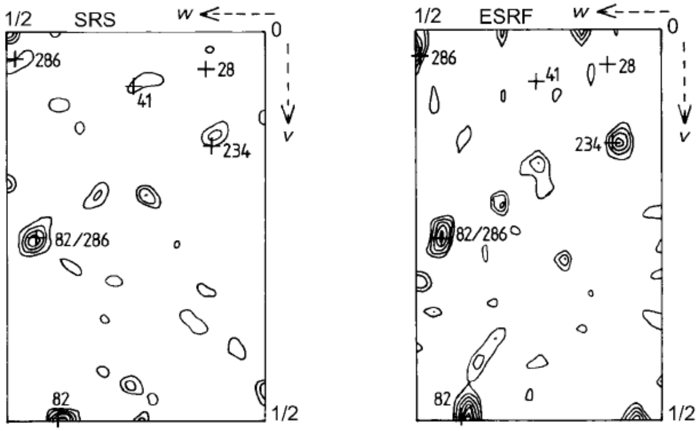

In Haedener et al 1999 Acta Cryst D the SRS 9.5 and ESRF BM14 comparison showed the viability of the new stations and of the ESRF electronic detector

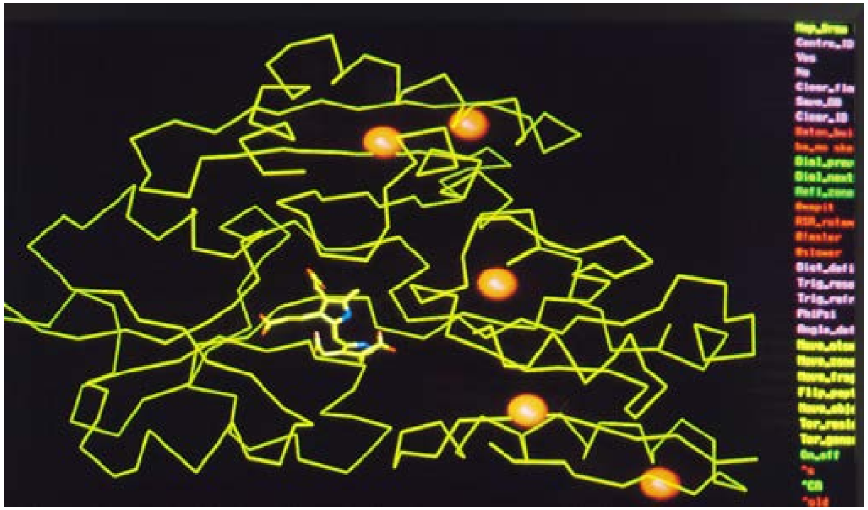

With Alfons Haedener, Basle Univ

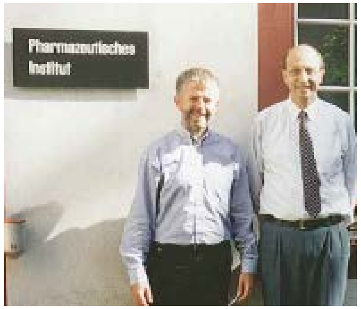

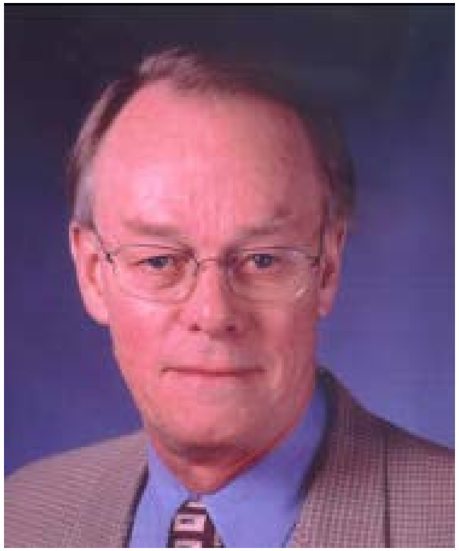

Inspired by Wayne Hendrickson

Manchester
Protein
Structure
Facility

Manchester DLS user remote access

- 50% of SR Time is Remote Access
  - *Short frequent shifts (5 hours)*
  - *Reduced reliance on in house X-Rays*

- Dramatic increase in throughput
  - *Hybrid Pixel detectors are FAST!*
  - *> 100 samples per shift (8 hours)*
  - *In-house we now focus on crystallogenesis*
  - *>1TB of data per month*

- What are the bottlenecks?
  - *Mounting + centering the samples!*
  - *Maintaining a supply of crystals*

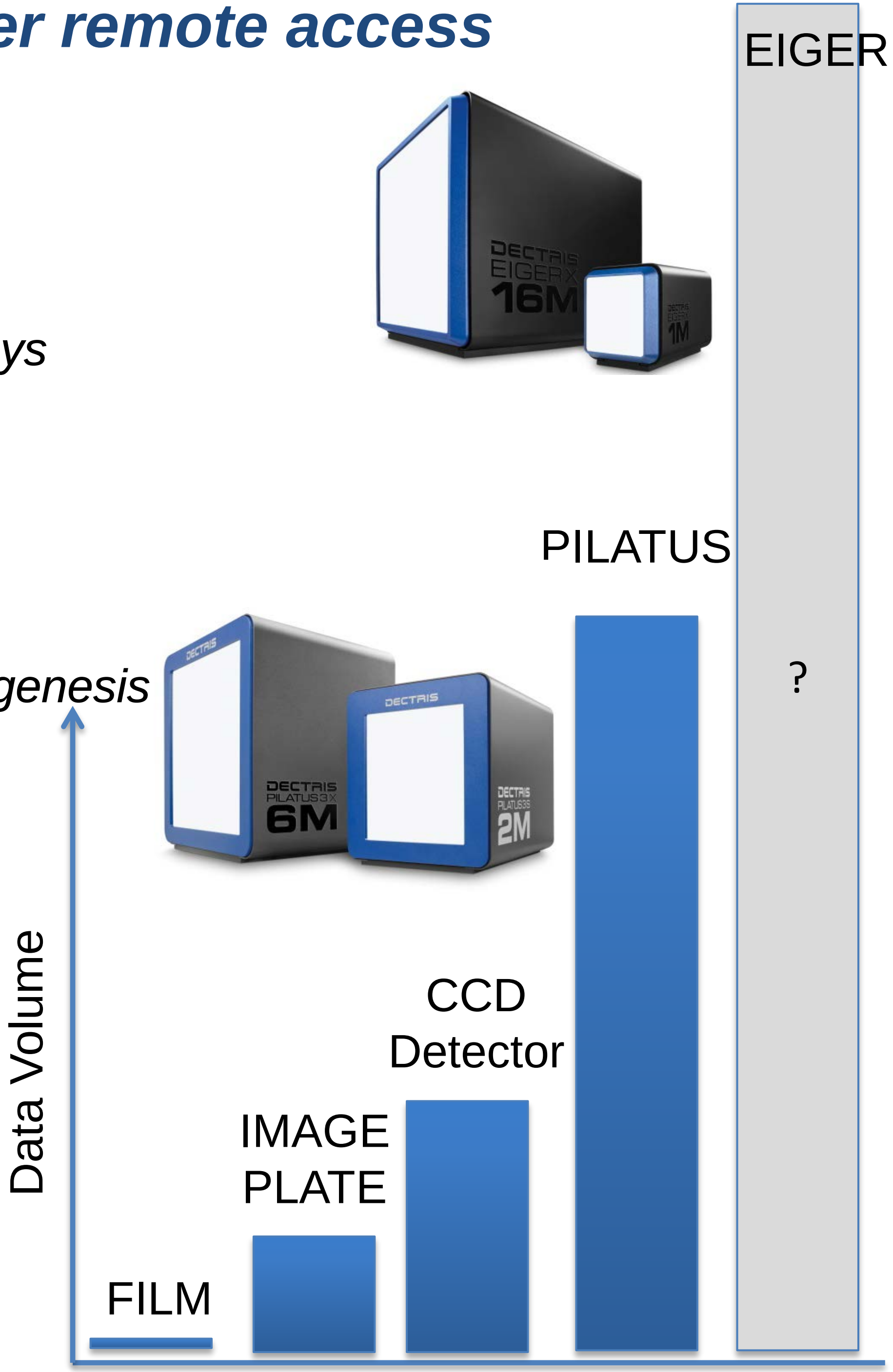

MANCHESTER 1824
The University of Manchester
Manchester Institute of Biotechnology

Kindly prepared by Dr. Colin Levy, MIB

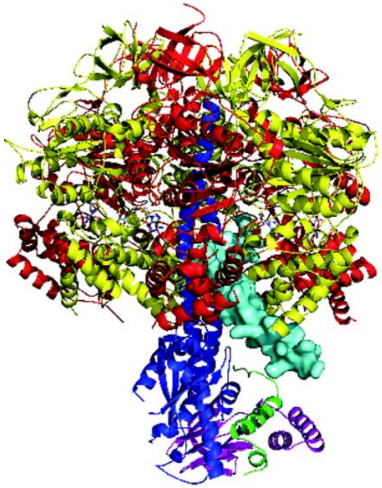

Such crystal structures of molecular machines transform the understanding of biology

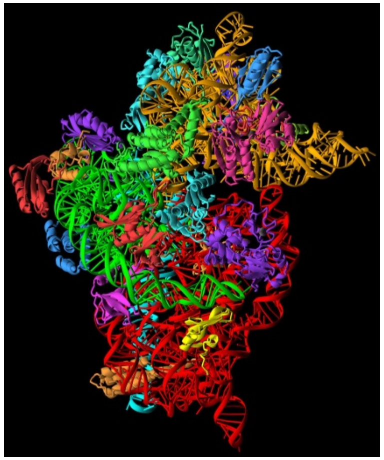

"Not only are SYNC-derived structures larger on average than HOME-derived ones, but they are also harder to determine because the B factors are larger. This represents the crux of the power of synchrotron radiation for macromolecular crystallography. "

J. Synchrotron Rad. (2004). **11**, 319-327

Protein Data Bank depositions from synchrotron sources

J Jiang and R M Sweet

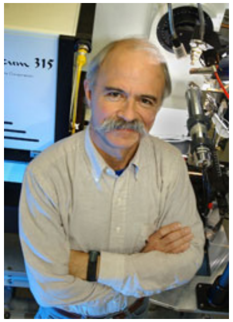

Bob Sweet

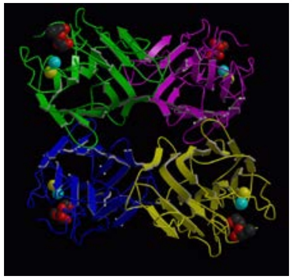

Concanavalin A studied ***with small molecule accuracy*** at 0.94Å resolution

Resolving protonation states

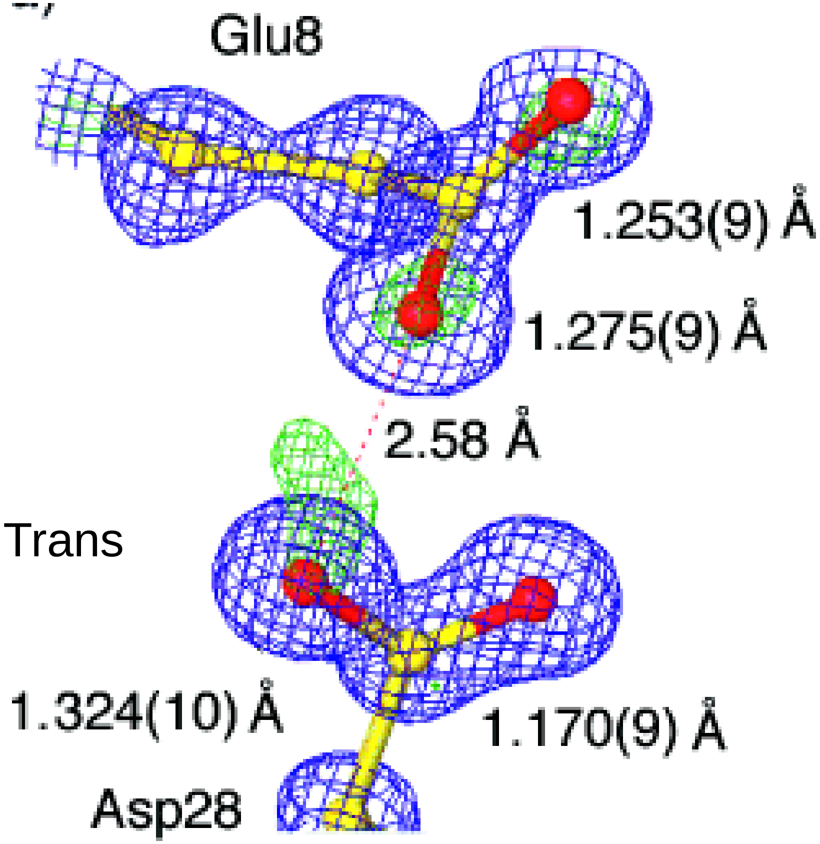

Deacon et al 1997
J Chem Soc Faraday Trans

Dauter, Jaskolski & Wlodawer J. Synchrotron Rad. (2010) 17, 433–444
"Without synchrotron radiation it would not be possible to obtain atomic resolution (defined as 1.2Å) or especially ultrahigh-resolution diffraction data from protein crystals."

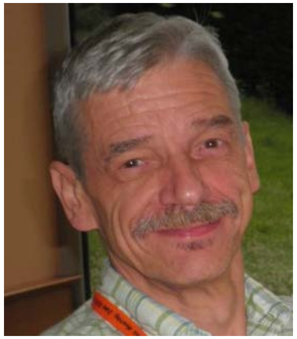

Zbysek Dauter

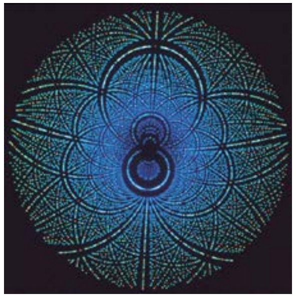

The SR Laue method was an unexpected development

Durward Cruickshank and I joined with Keith on this!

A key result from SRS 9.6: learning that, by far, the Bragg reflections were in singlet Laue spots and which contradicted the early pioneers' writings such as W L Bragg

Colour coding:- →
Green are singlet reflections Laue spots;
Other colours are for doublets, triplets etc

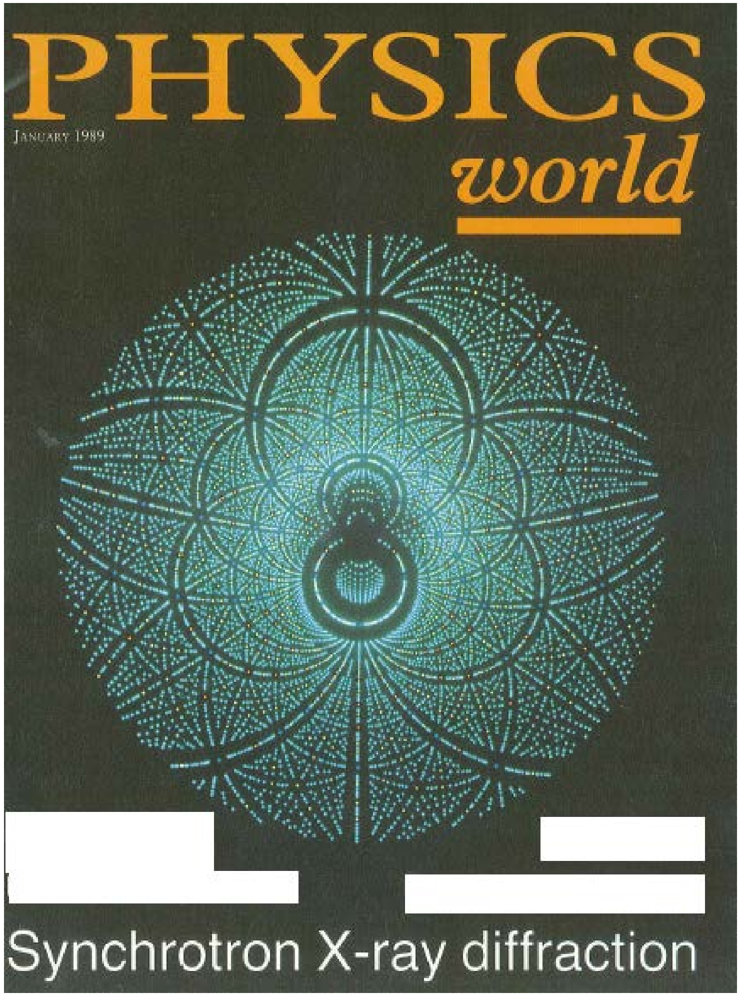

Cruickshank, Helliwell & Moffat Acta. Cryst. A43, 656-674 (1987)

Moffat:
"With the ESRF or APS a single bunch allows nsec Laue protein crystallography"

C H M

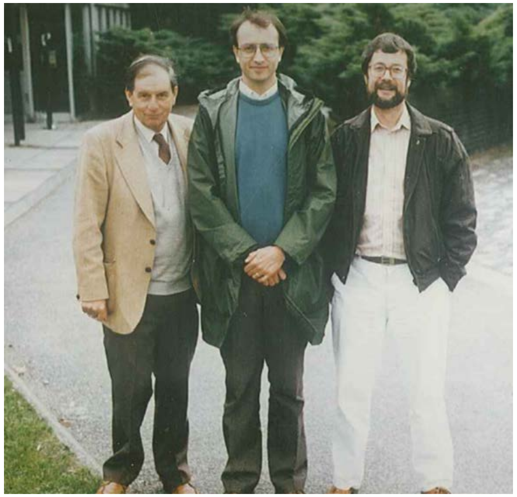

Outside York University Physics Dept

Time-resolved: spectacularly Keith Moffat used SR Laue to do nanosecond protein crystallography with CO myoglobin then PYP

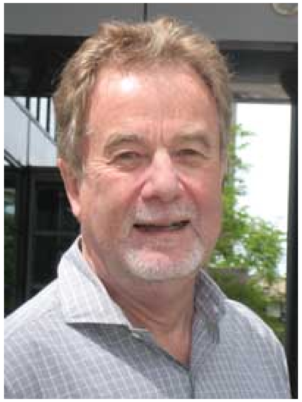

Keith Moffat

Time!

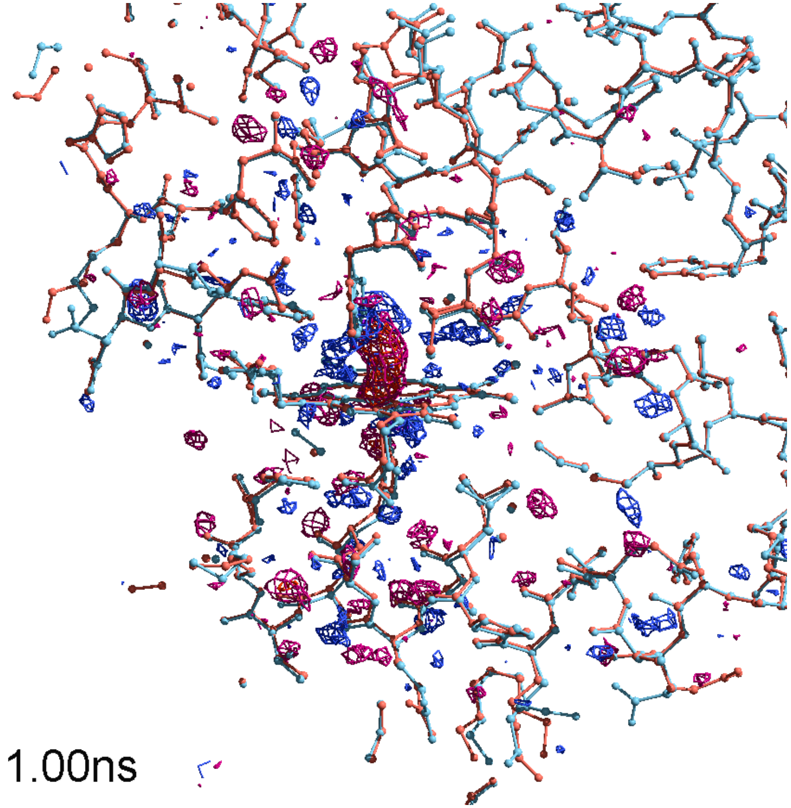

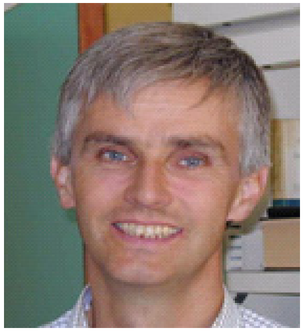

Michael Wulff, ESRF

Šrajer, Ren, Teng, Schmidt, Ursby, Bourgeois, Pradervand, Schildkamp, Wulff and Moffat
Protein Conformational Relaxation and Ligand Migration in Myoglobin:
A Nanosecond to Millisecond Molecular Movie from Time-Resolved Laue X-ray Diffraction.
Biochemistry (2001), **40** (46):13802–15

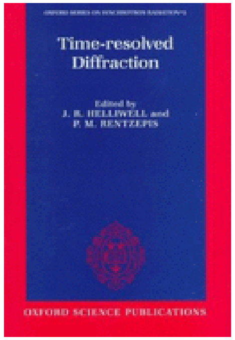

Time-resolved structures of ***hydroxymethylbilane synthase***

Helliwell et al J Chem Soc Faraday Trans 1998, 94(17), 2615–2622

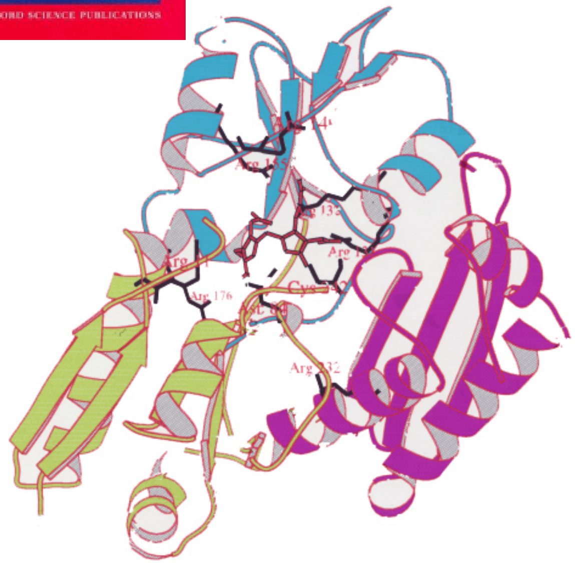

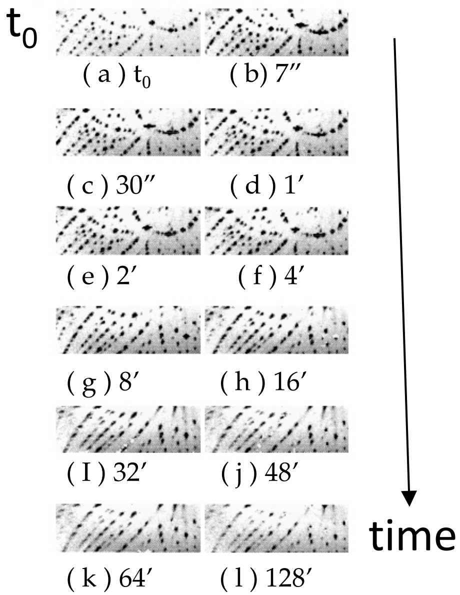

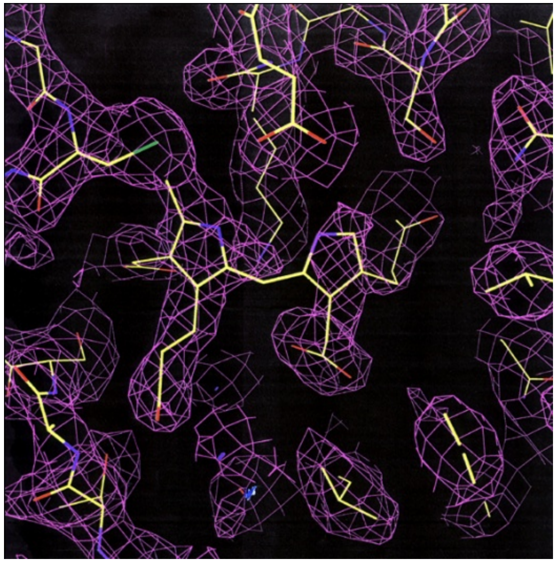

Good Laue maps using their EAD.

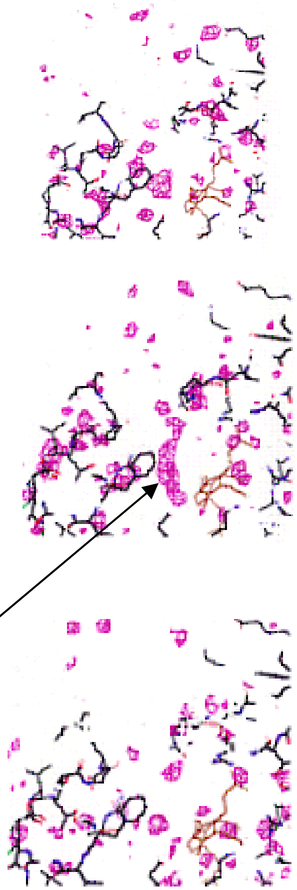

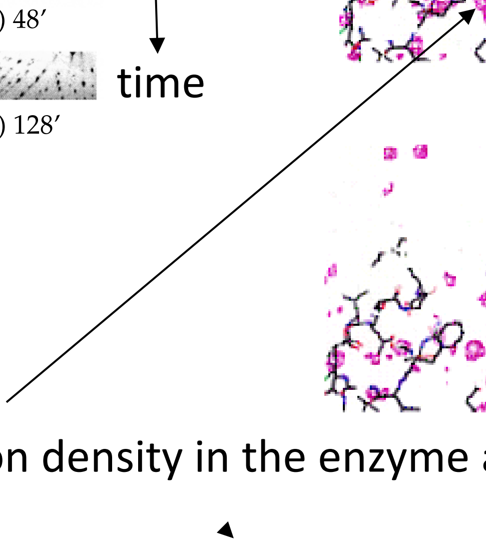

Transient electron density in the enzyme active site

The Laue method ***and the Daresbury Laue data processing*** software were enthusiastically taken up at the Grenoble neutron reactor source>>>

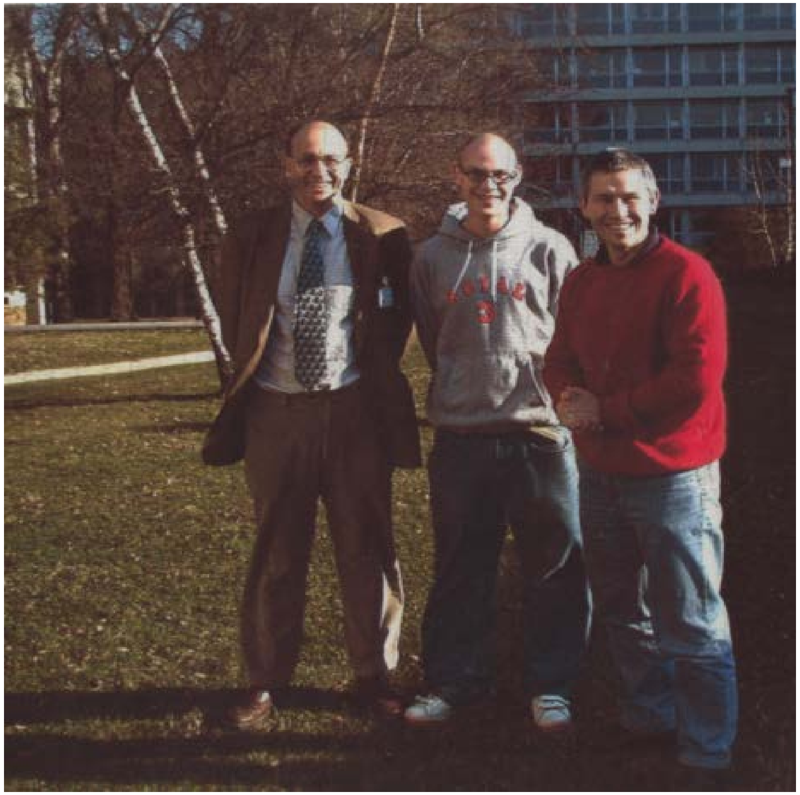

Matthew Blakeley
Dean Myles

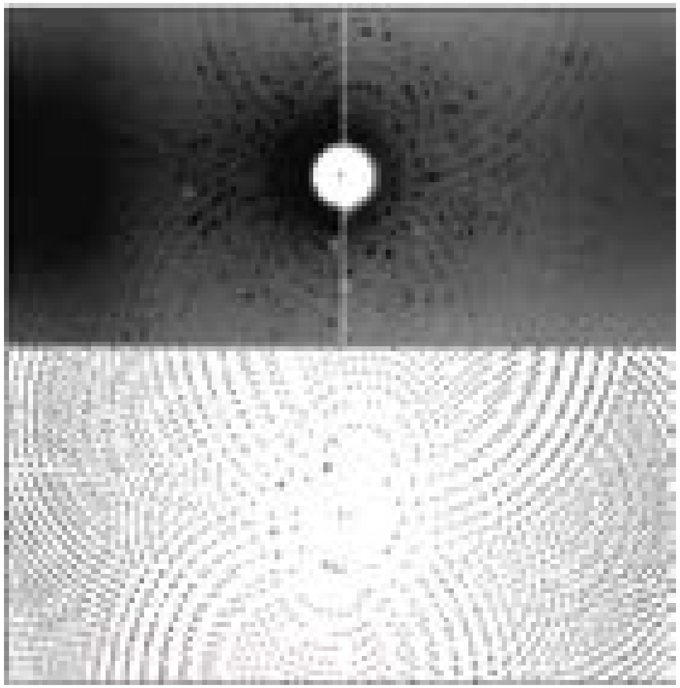

50kDa glucoside concanavalin A
$I2_13$ a=168 Å
Gilboa, Myles, Habash, Raftery & Helliwell
(2001) J Appl Cryst 34, 454-457

Spin off: the EMBL Laue neutron Diffractometer in Grenoble & more efficient use of many more of the emitted neutrons

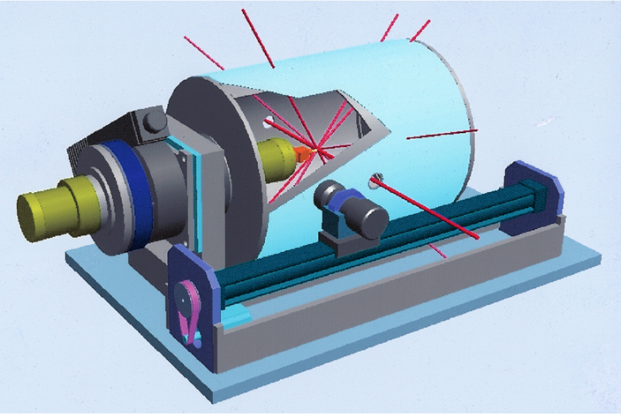

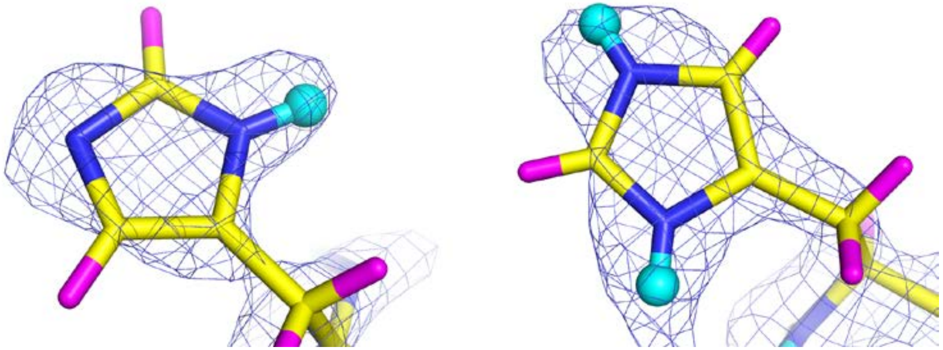

Resolving histidine protonation states with neutrons

> Gd_2O_3-doped neutron sensitive image plate

- Typical neutron beam wavelength range: ~2.7 – 3.6 Å

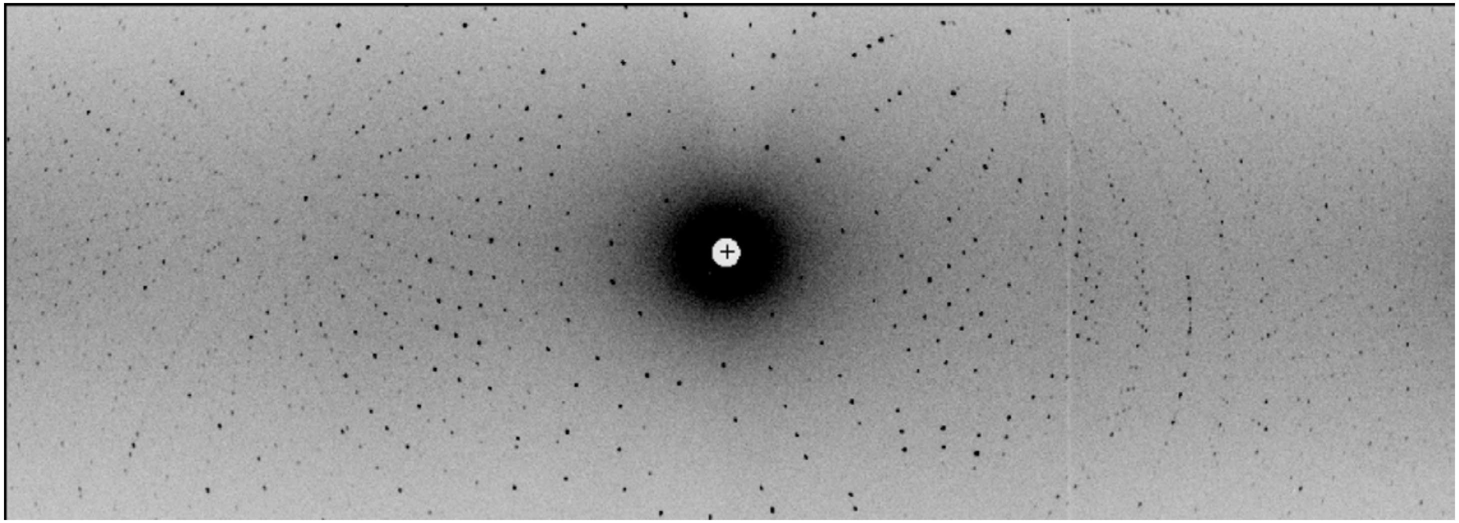

Neutron Laue data are processed with the 'Daresbury Laue software'; this software was validated with case studies from SR Laue data measured on SRS 9.6 and at ESRF ID09.

Current & future beamline developments

Microbeam scanning to select the best portion of a crystal

A diffraction grid scan 9 x 9 micron beam in a typical raster of 20 x 20 micron

Alpha-crustacyanin crystal

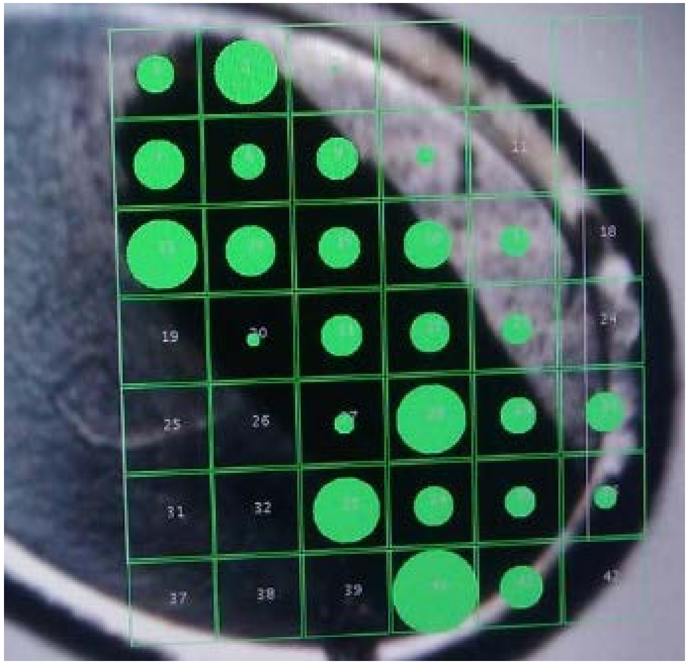

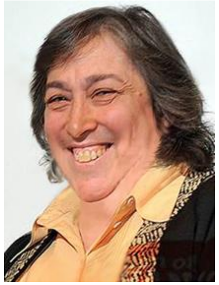

Example JRH use of **DLS microfocus beamline**

Naomi Chayen

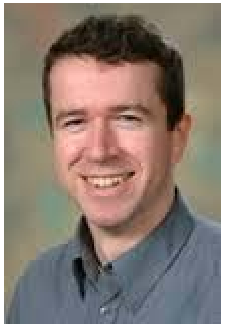

Underway at DLS is the design, construction and operation of the submicron ***VMXm beamline*** aimed at delivering a ***beam size of between 0.5 and 5 microns***.

Gwyndaf Evans

For a recent overview of SR microcrystal diffraction see
Macromolecular microcrystallography
Evans, Axford, Waterman & Owen (2011) Cryst Rev 17, 105-142

ESRF ID 13 optical schemes and X-ray foci available

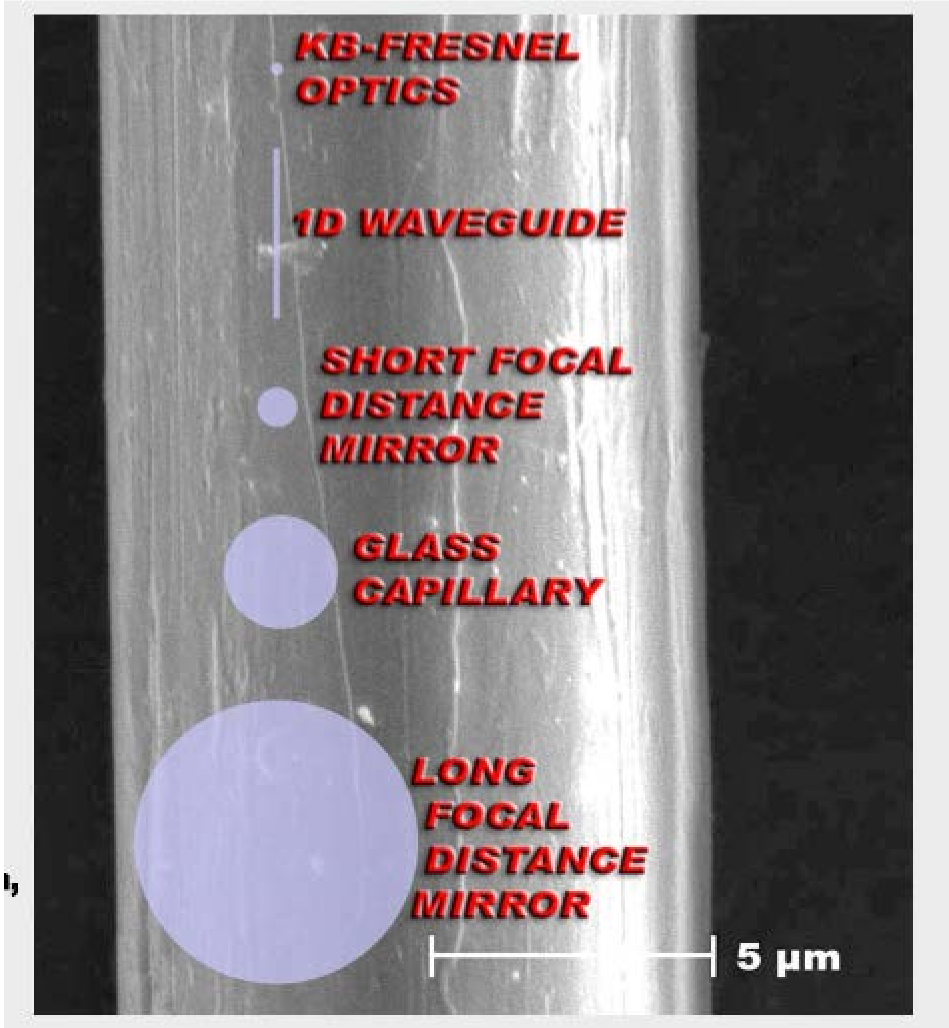

http://www.esrf.eu/files/live/sites/www/files/UsersAndScience/Experiments/SoftMatter/ID13/poster/esrf_um_2005.jpg

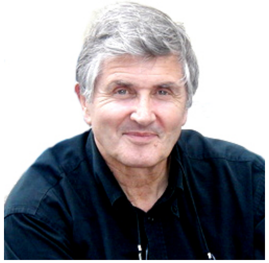

Roger Fourme

Enthusiasm for ultra short wavelength

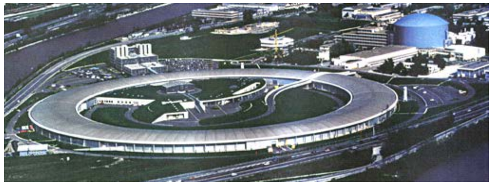

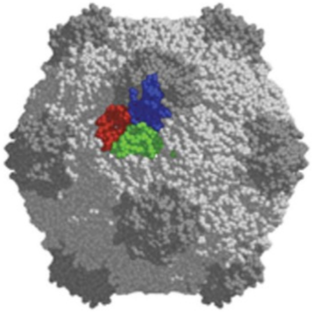

Cowpea Mosaic Virus
0.35 GPa

J. Synchrotron Rad. (2011). **18**, 31-36
A new paradigm for macromolecular crystallography beamlines derived from high-pressure methodology and results
R. Fourme, E. Girard, A.-C. Dhaussy, K. Medjoubi, T. Prangé, I. Ascone, M. Mezouar and R. Kahn

J. Appl. Cryst. (2012). **45**, 652-661
Reduction of radiation damage and other benefits of short wavelengths for macromolecular crystallography data collection
R. Fourme, V. Honkimäki, E. Girard, K. Medjoubi, A.-C. Dhaussy and R. Kahn

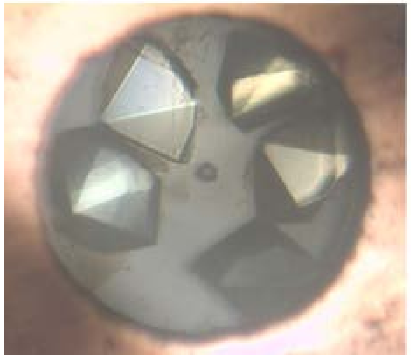

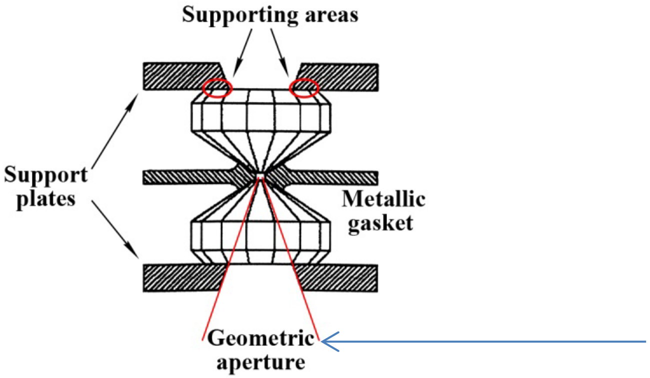

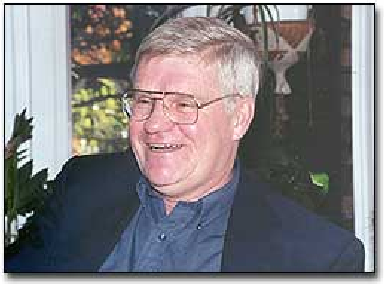

Jack Johnson

Protein Crystallography at Ultra-Short Wavelengths

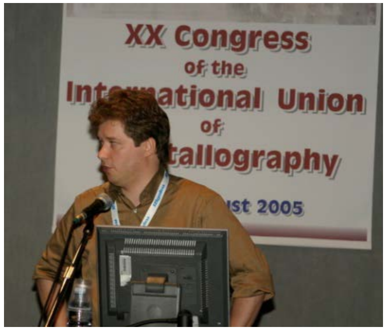

Marc Schiltz

Xe K edge & elastase

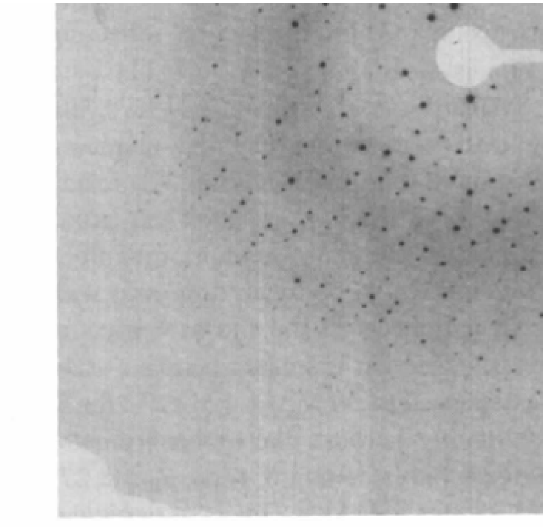

Ho K edge & HEWL

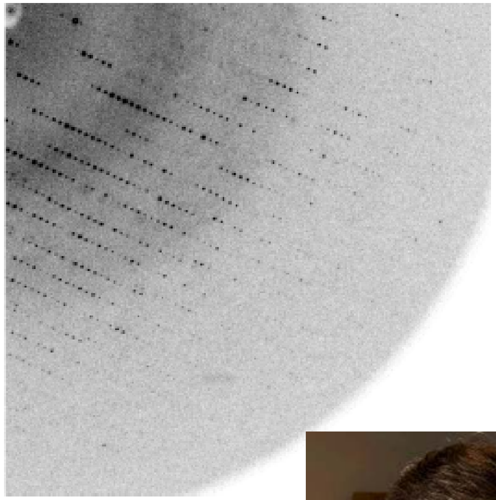

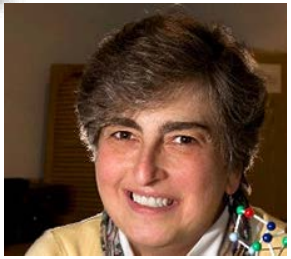

Vivian Stojanoff

J. Synchrotron Rad. (1997). **4**, 287-297 [
M. Schiltz, **Å. Kvick**, **O. S. Svensson**, **W. Shepard**,
E. de La Fortelle, **T. Prangé**, **R. Kahn**, **G. Bricogne** **and** **R. Fourme**

J. Appl. Cryst. (2006). **39**, 831-841
J. Jakoncic, **M. Di Michiel**, **Z. Zhong**, **V. Honkimaki**, **Y. Jouanneau** **and** **V. Stojanoff**

Enthusiasm for MX at longer wavelengths for eg K edges of sulphur and phosphorous!!

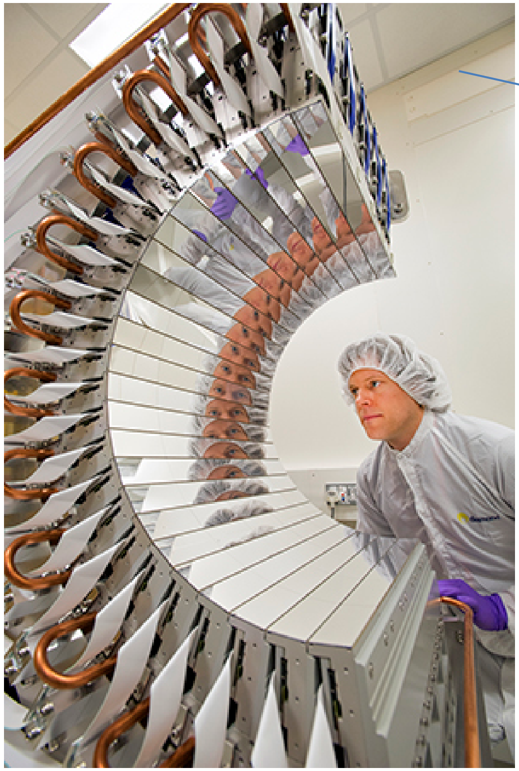

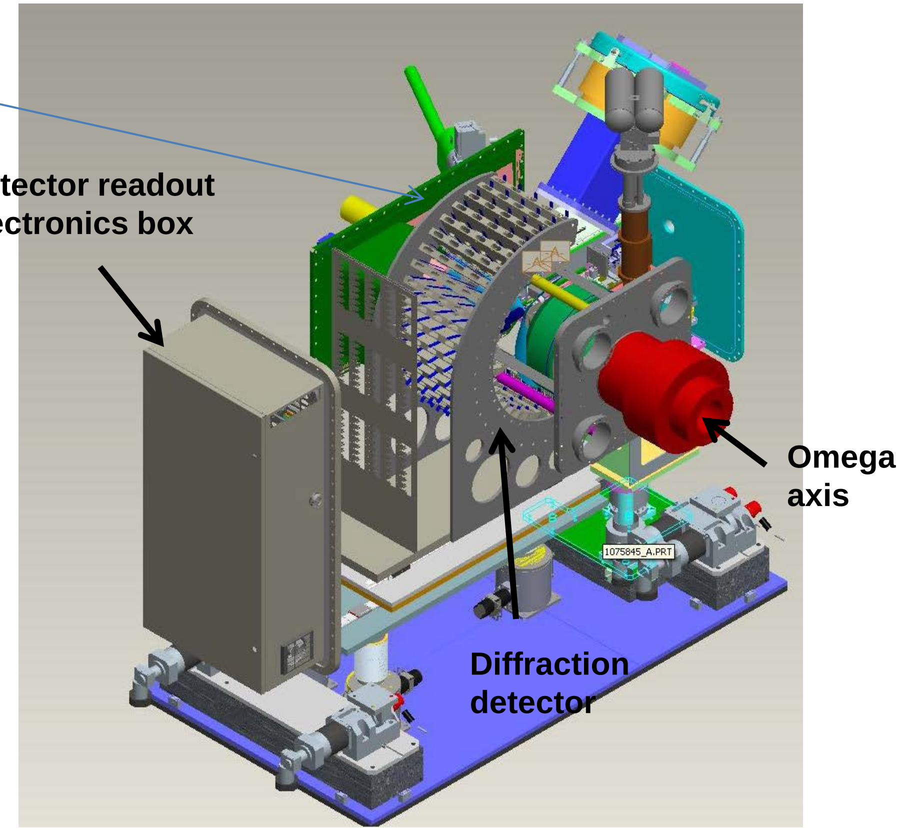

DECTRIS
Next Generation X-Ray Detectors

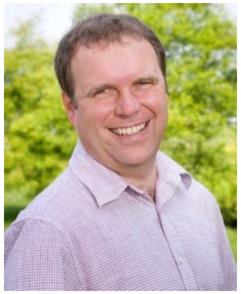

A masterpiece of instrumentation!!

DLS I23: in-vacuum end station led by Armin Wagner (Lecture in IUCr MS-40)

New X-ray sources

the national 3rd generation sources; the ultimate storage rings; the X-ray lasers

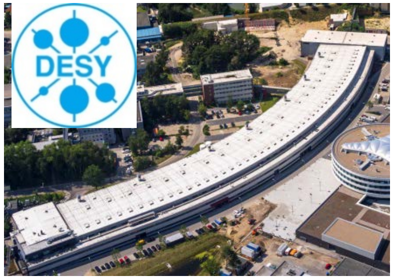

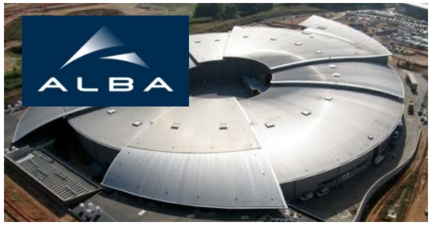

- New SR sources: PETRA III the most brilliant ie the ‘ultimate storage ring’ ; also soon NSLS II, MAX IV
- Diamond Light Source is a cutting edge source for UK with a vibrant research and development programme
- ALBA likewise is a cutting edge SR source for Spain
- Soleil for France…..

- ESRF has a new upgrade programme for nanofocus beams; also APS upgrade at advanced planning

- The X-ray laser: towards nanoclusters of molecules

BBC Sign in | News | Sport | Weather | iPlayer | TV

NEWS SCIENCE & ENVIRONMENT

Home | World | UK | England | N. Ireland | Scotland | Wales | Business | Politics | Health | Education | Sci

4 July 2014 Last updated at 11:33

Share

Upgrade for Europe's big X-ray light source

Expected characteristic of an ESRF Phase II MX beamline

	Current	New Lattice (current optics)	New lattice (perfect optics)	New Lattice (50:1)
**Beamsize @ sample (μm^2)**	**~60 x 30**	30 x 25	20 x 4	**1.2 x 0.2**
**Flux @ sample (ph/sec)**	**~1 x 10^{13}**	~1 x 10^{14}	~1 x 10^{14}	**~1 x 10^{14}**

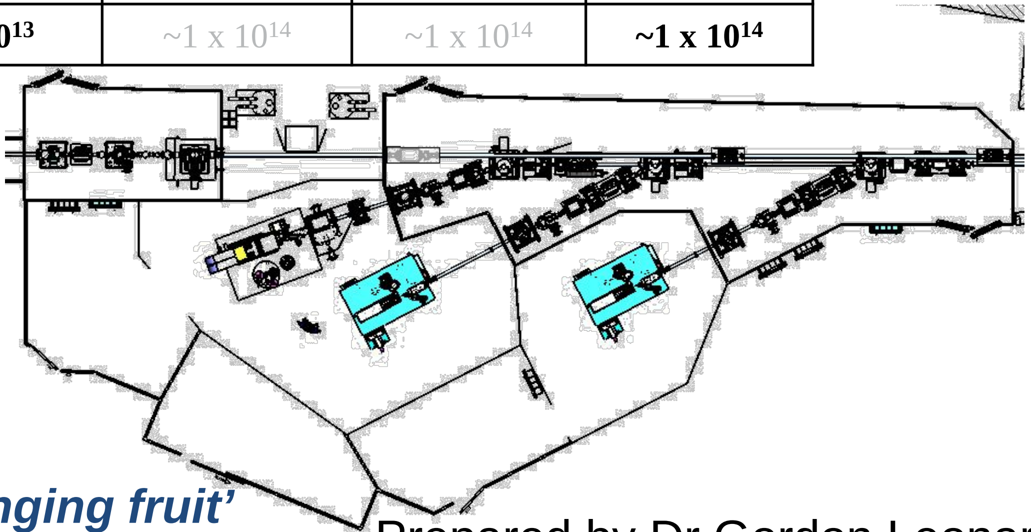

MASSIF

Massively Automated

Sample Selection

Integrated Facility

to assist with reaching the 'high hanging fruit'

Prepared by Dr Gordon Leonard

Spin offs

SR commercial MX usage; SR chemical crystallography ; neutron MX

Spin off: ***Daresbury Analytical and Research Technical Services*** (DARTS); an important aspect of the Economic Impact of the SRS

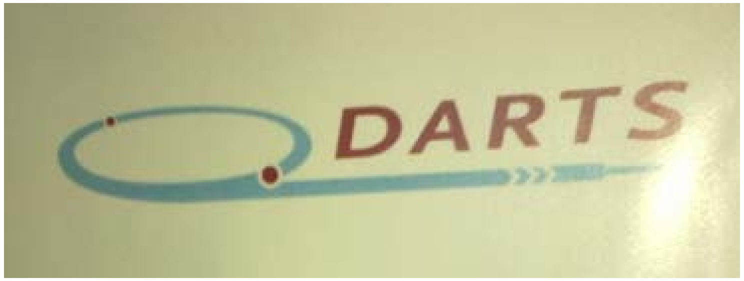

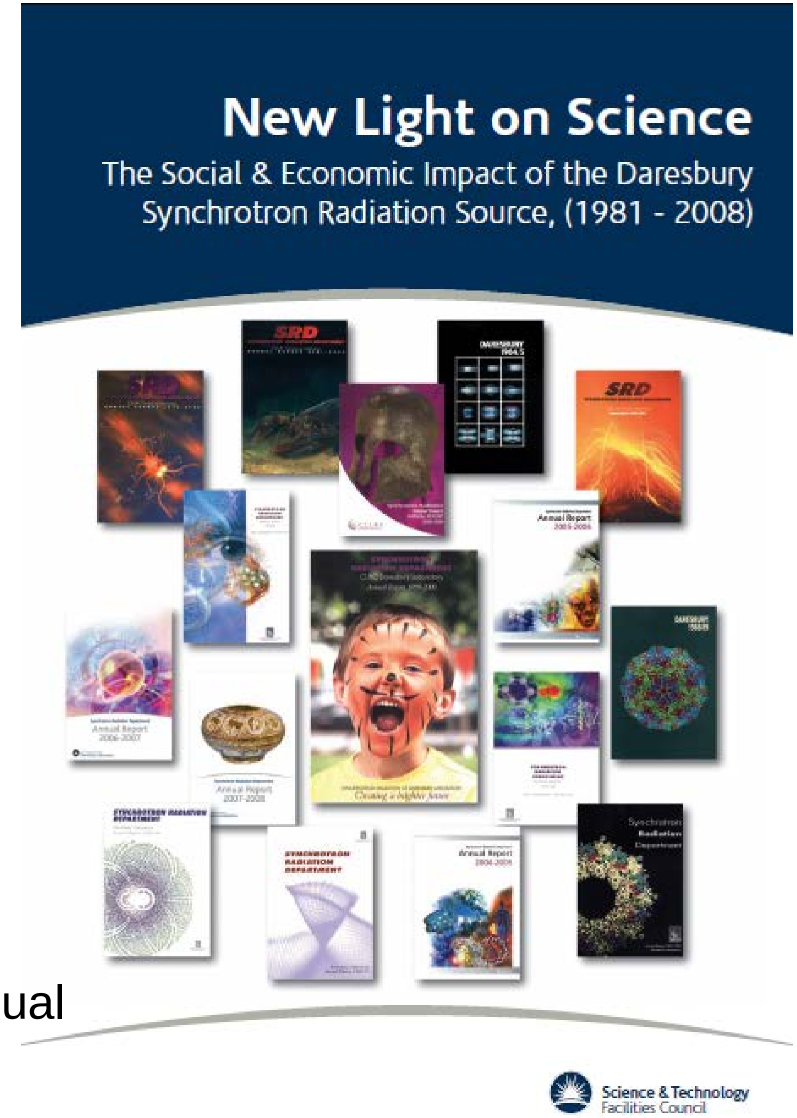

E.J. Maclean, P.J. Rizkallah and J.R. Helliwell (2006) Protein Crystallography and Synchrotron radiation European Pharmaceutical Review Issue 2, p71-76

Approx £300k income to SRS per annum;

Australia and Canada each pushed on the usual ceiling of a '10% maximum' commercial use.

Canadian Light Source World ranked industrial use: 12 to 15% average and upto 25% allowed per beamline

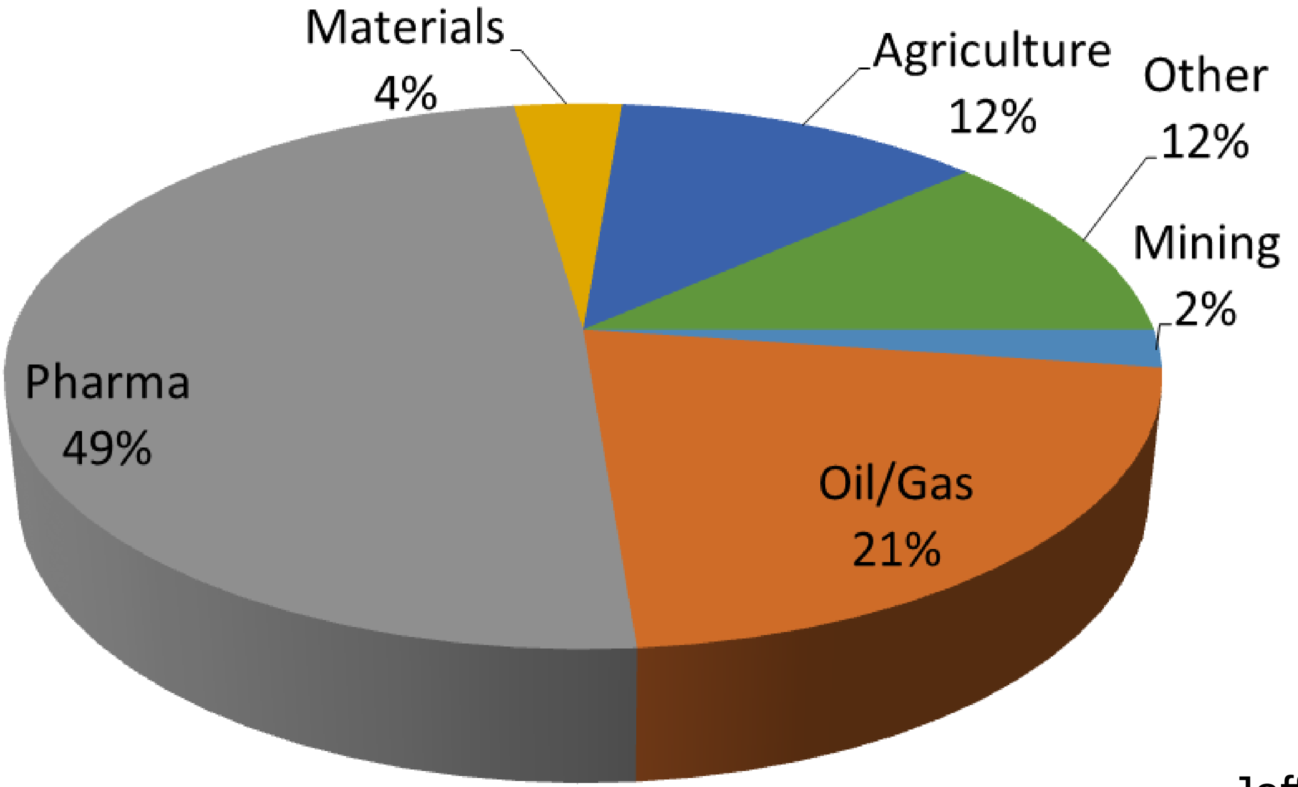

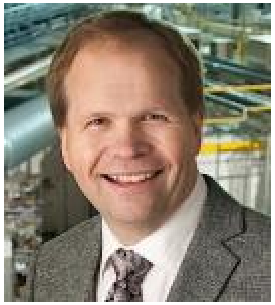

Jeffrey Cutler, CLS

“Analytical services comprises 65% industrial useage of CLS and Collaborative longer term research comprises 35%.
Overall CLS has served more than 50 industrial clients with Can$2.4M revenue to CLS since 2005/6.” ***Jeffrey Cutler*** *SR News (2014) 27, 3, p3-6.*

SR chemical crystallography early details at SRS PX 9.6

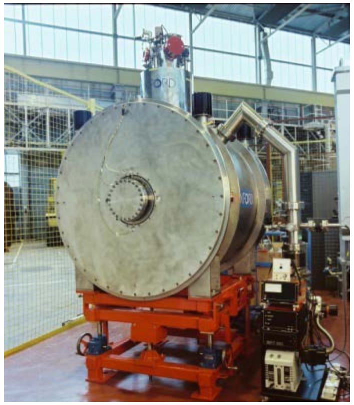

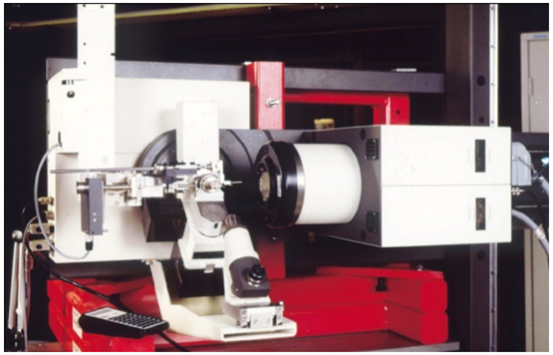

As well as film we broke new ground with the ***Enraf Nonius*** *FAST TV diffractometer; this was the design of* ***Uli Arndt*** *& is shown installed on SRS 9.6*

Helliwell et al NIM (1986) A246, 617-623; ***tuneable λ (0.6 to 1.5Å)*****.**

S.J. Andrews, M.Z. Papiz, R. McMeeking, A.J. Blake, B.M. Lowe, K.R. Franklin, J.R. Helliwell and M.M. Harding 'Piperazine Silicate (EU-19) - The structure of a very small crystal determined with synchrotron radiation' Acta. Cryst. B44, 73-77 (1988)

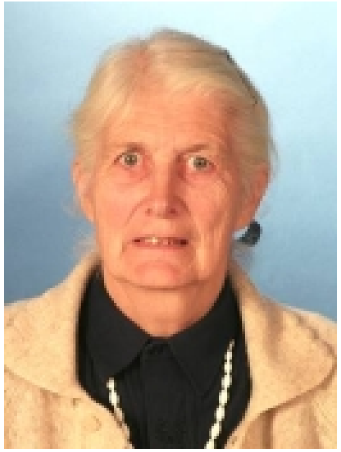

Marjorie Harding

Marjorie Harding's work on SRS 9.6 led onto SRS 9.8, SRS16.2 and DLS I19

Spin off: MAD chemical crystallography using SRS9.8:

an 11 wavelength expt of a gallium substituted ZnULM5

λ_3 scaled to λ_5 = Zn

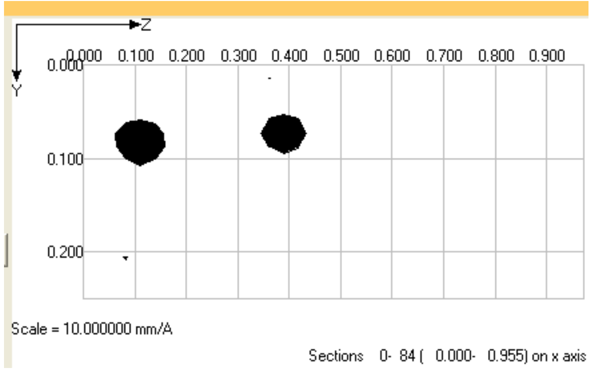

Delta anom λ_1 = Zn

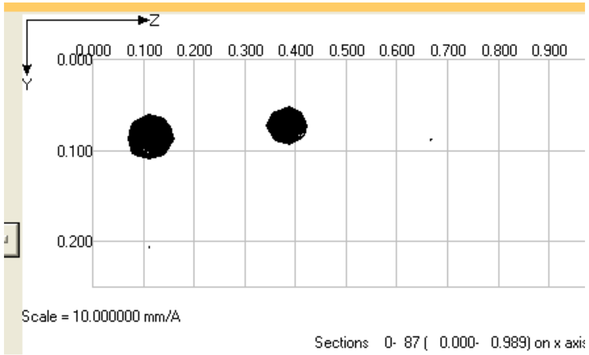

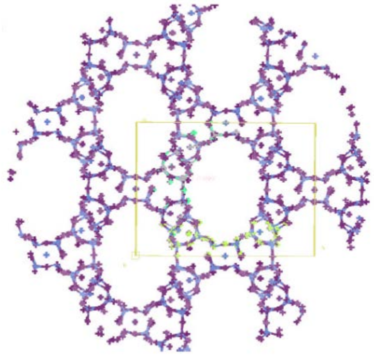

λ_9 scaled to λ_7 = Ga + smaller Zn

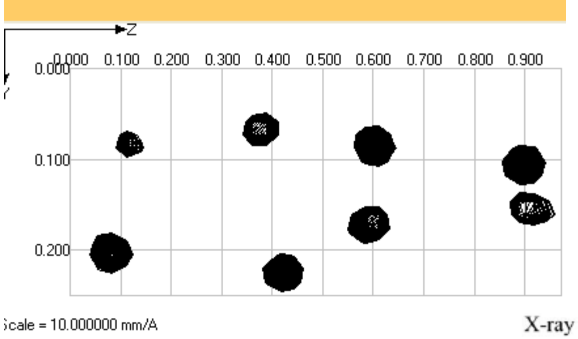

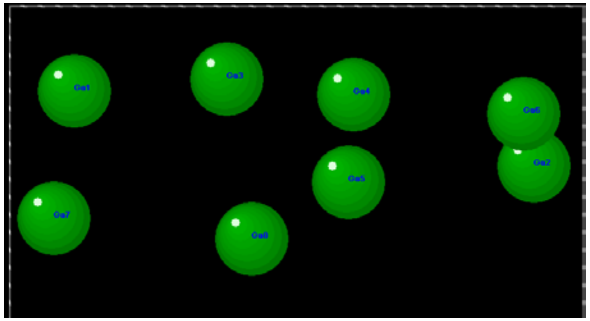

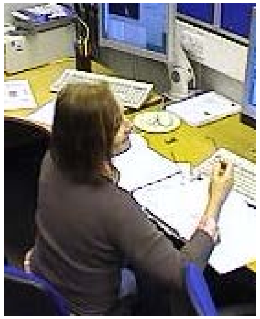

Madeleine Helliwell

M Helliwell, J. R. Helliwell, V. Kaucic, N. Zabukovec Logar, S. J. Teat, J. E. Warren and E. J. Dodson (2010) Acta Cryst B66, 345

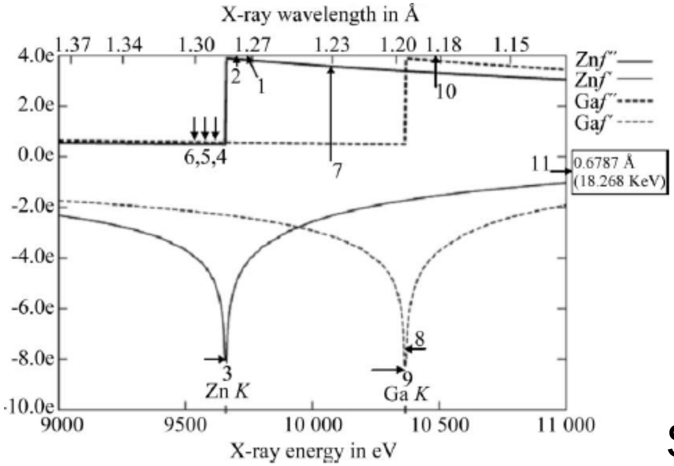

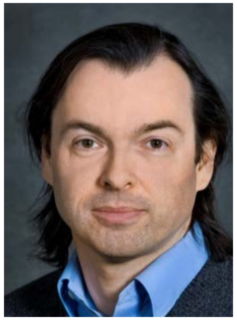

Simon Teat

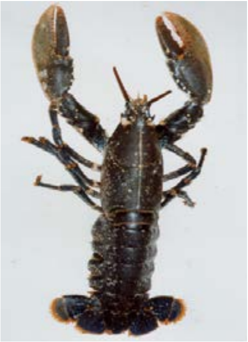

Public understanding of SR and structural Science: marine colouration in lobster shell!

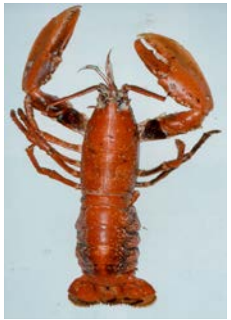

Huge public interest and also from school children

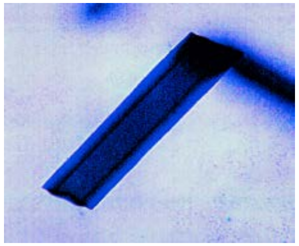

β-Crustacyanin crystal

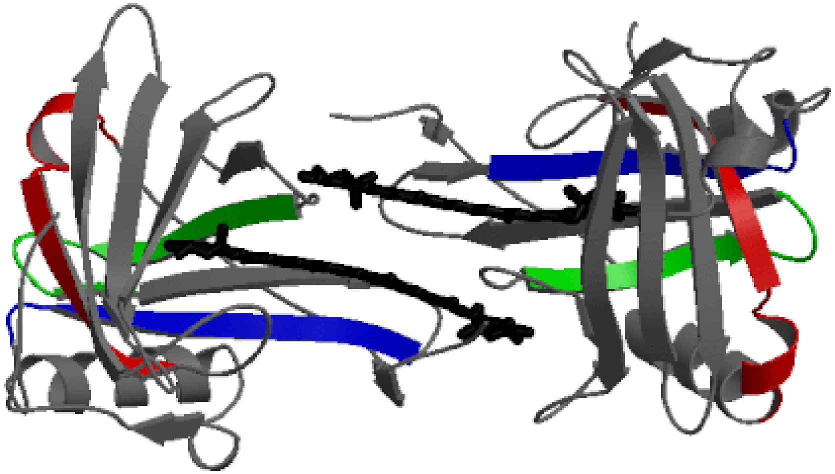

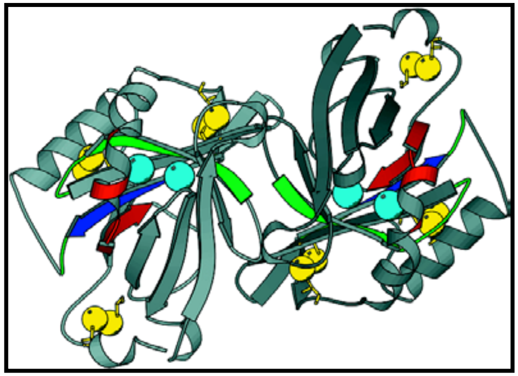

Apocrustacyanin A1

λ 2Å !!

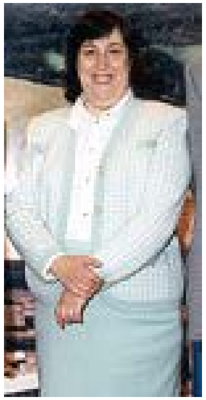

Naomi Chayen

Cianci et al (2002) PNAS USA 99, 9795

'The molecular basis of the coloration mechanism in lobster shell: β-crustacyanin at 3.2 A resolution'

Cianci et al (2001) Acta Cryst D57, 1219

*"Structure of apocrustacyanin A1 using **softer X-rays**"*

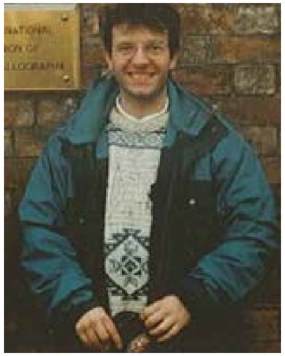

Mike Cianci

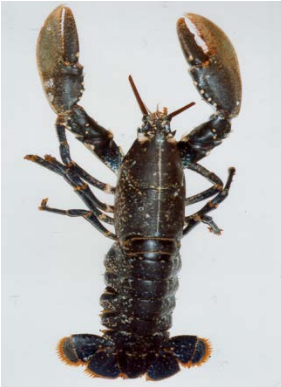

Lobster crustacyanin hits the media! starting from work at SRS in the early 2000s

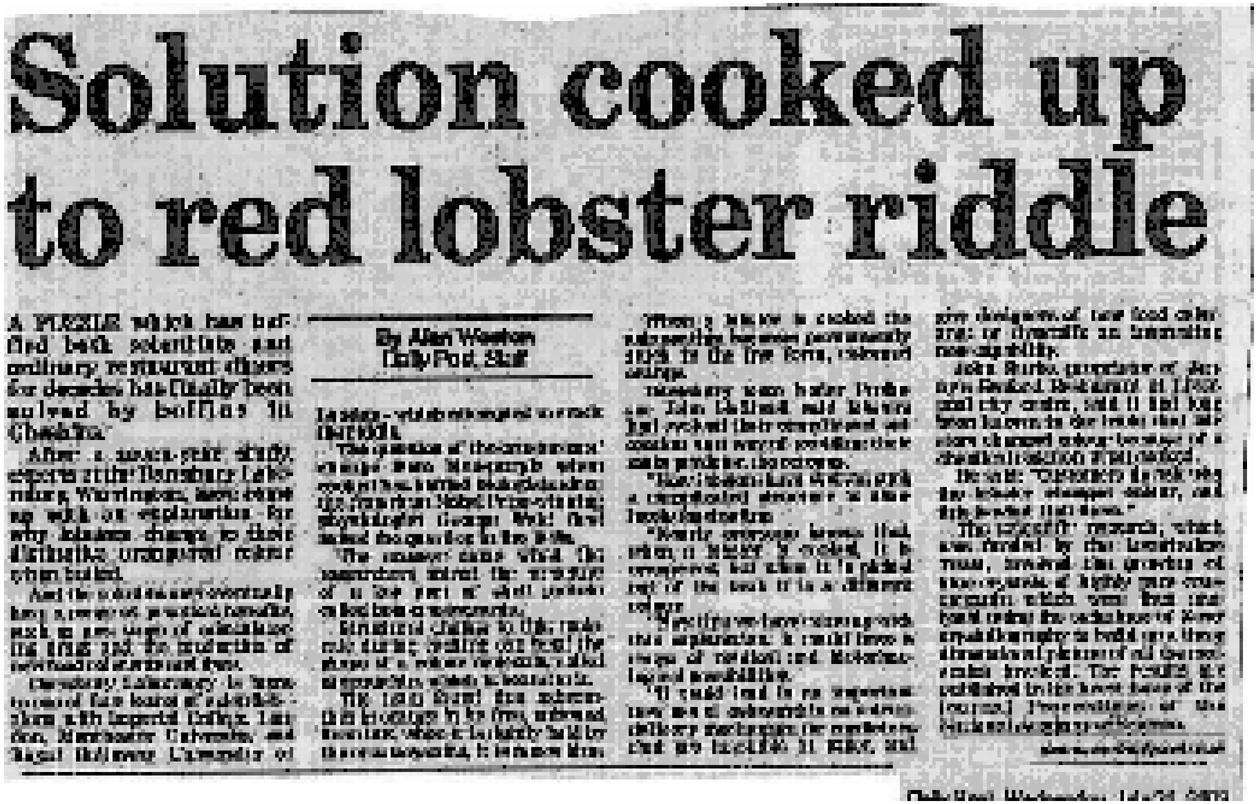

Solution cooked up to red lobster riddle

By Alan Weston
Daily Post Staff

[illegible]

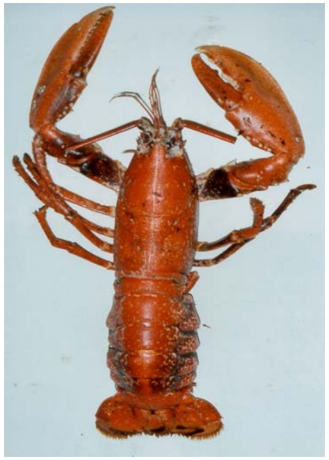

From the Liverpool Daily Post but articles also in The Times,
The Guardian, The Independent also Radio, TV, Scientific American,
New Scientist, Physics Today etc.

I have tried to show you that the field is very dynamic: whether considering new X-ray sources, beamline developments as well as our science and spin offs

Acknowledgements

- I heartily thank all the SR and neutron facilities for their collaboration
- I heartily thank my PhD students and PostDocs over the last 35 years
- Daresbury Laboratory & the Universities of York, Oxford, Keele & Manchester
- For specific assistance with slides I thank: Dr Pawel Grochulski (CLS), Dr Armin Wagner (DLS), Dr Gordon Leonard (ESRF), Dr Matthew Blakeley (Inst Laue Langevin) and Dr Colin Levy (Manchester Institute of Biotechnology).

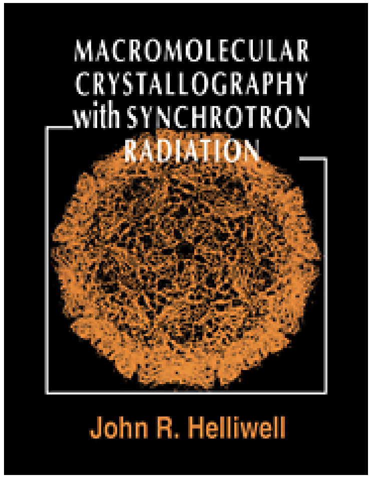
MACROMOLECULAR
CRYSTALLOGRAPHY
with SYNCHROTRON
RADIATION
John R. Helliwell

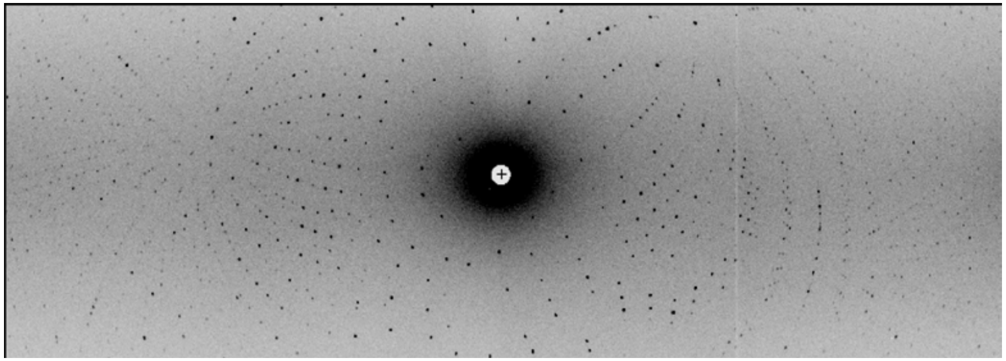

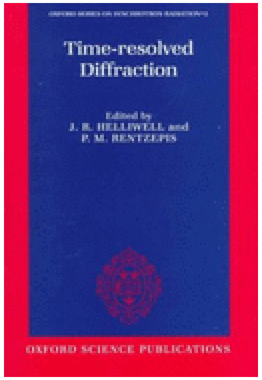
Time-resolved
Diffraction
Edited by
J. R. HELLIWELL and
P. M. RENTZEPIS
OXFORD SCIENCE PUBLICATIONS

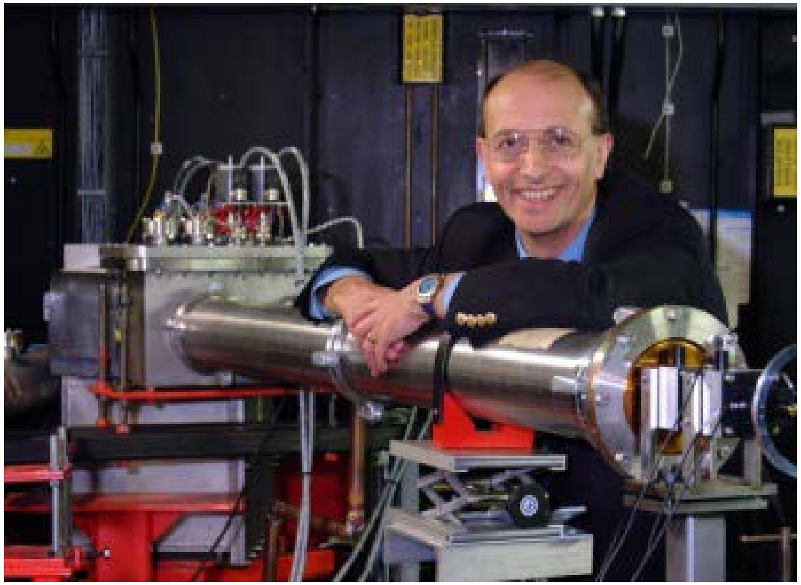

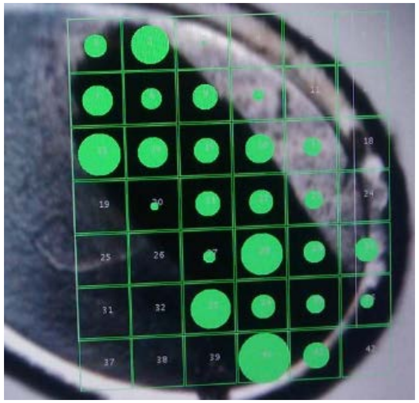

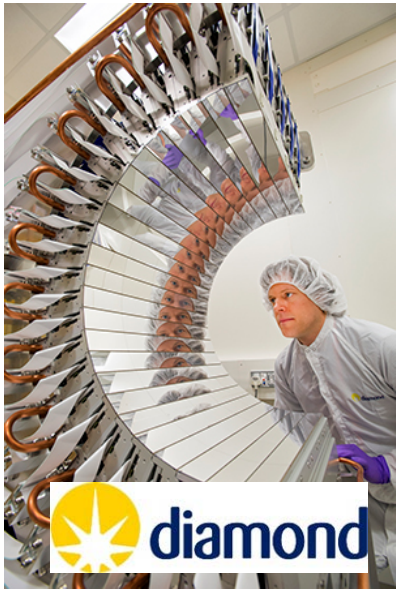
diamond

Thank you

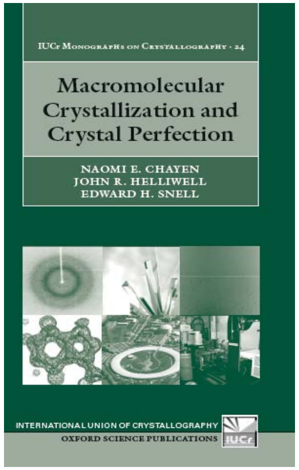
IUCr Monographs on Crystallography · 24
Macromolecular
Crystallization and
Crystal Perfection
NAOMI E. CHAYEN
JOHN R. HELLIWELL
EDWARD H. SNELL
INTERNATIONAL UNION OF CRYSTALLOGRAPHY
OXFORD SCIENCE PUBLICATIONS
