# Supplementary material for: Synchrotron radiation macromolecular crystallography: science and spin-offs
Source: IUCrJ. 2015 Feb 3;2(Pt 2):283–91. doi: 10.1107/S205225251402795X (PMC4392420; doi:10.1107/S205225251402795X)
Supplement: Supplementary file 3 [file m-02-00283-sup3.pptx]

## Slide 1
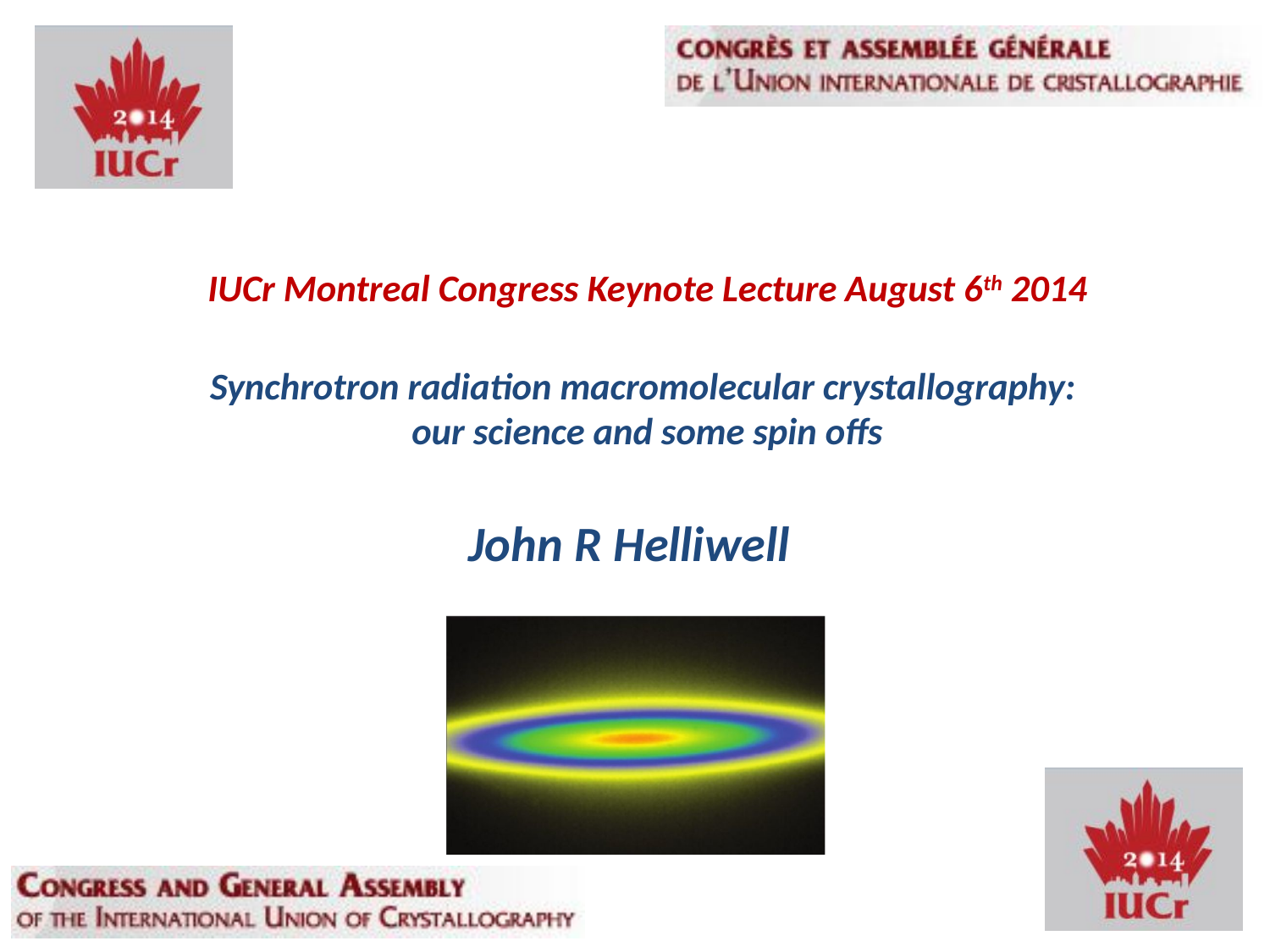

# IUCr Montreal Congress Keynote Lecture August 6th 2014 Synchrotron radiation macromolecular crystallography: our science and some spin offs
John R Helliwell

## Slide 2
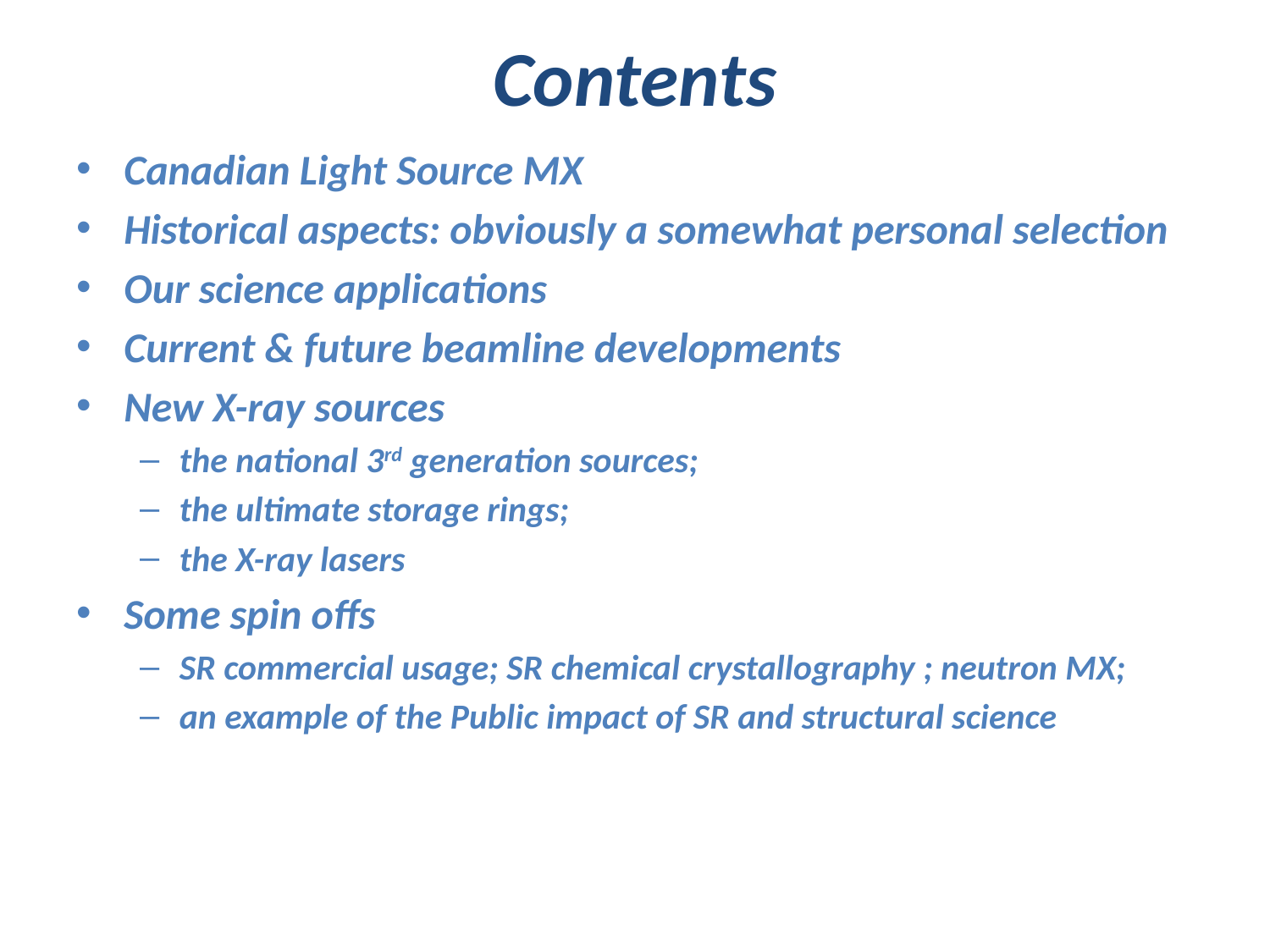

# Contents
Canadian Light Source MX
Historical aspects: obviously a somewhat personal selection
Our science applications
Current & future beamline developments
New X-ray sources
the national 3rd generation sources;
the ultimate storage rings;
the X-ray lasers
Some spin offs
SR commercial usage; SR chemical crystallography ; neutron MX;
an example of the Public impact of SR and structural science

## Slide 3
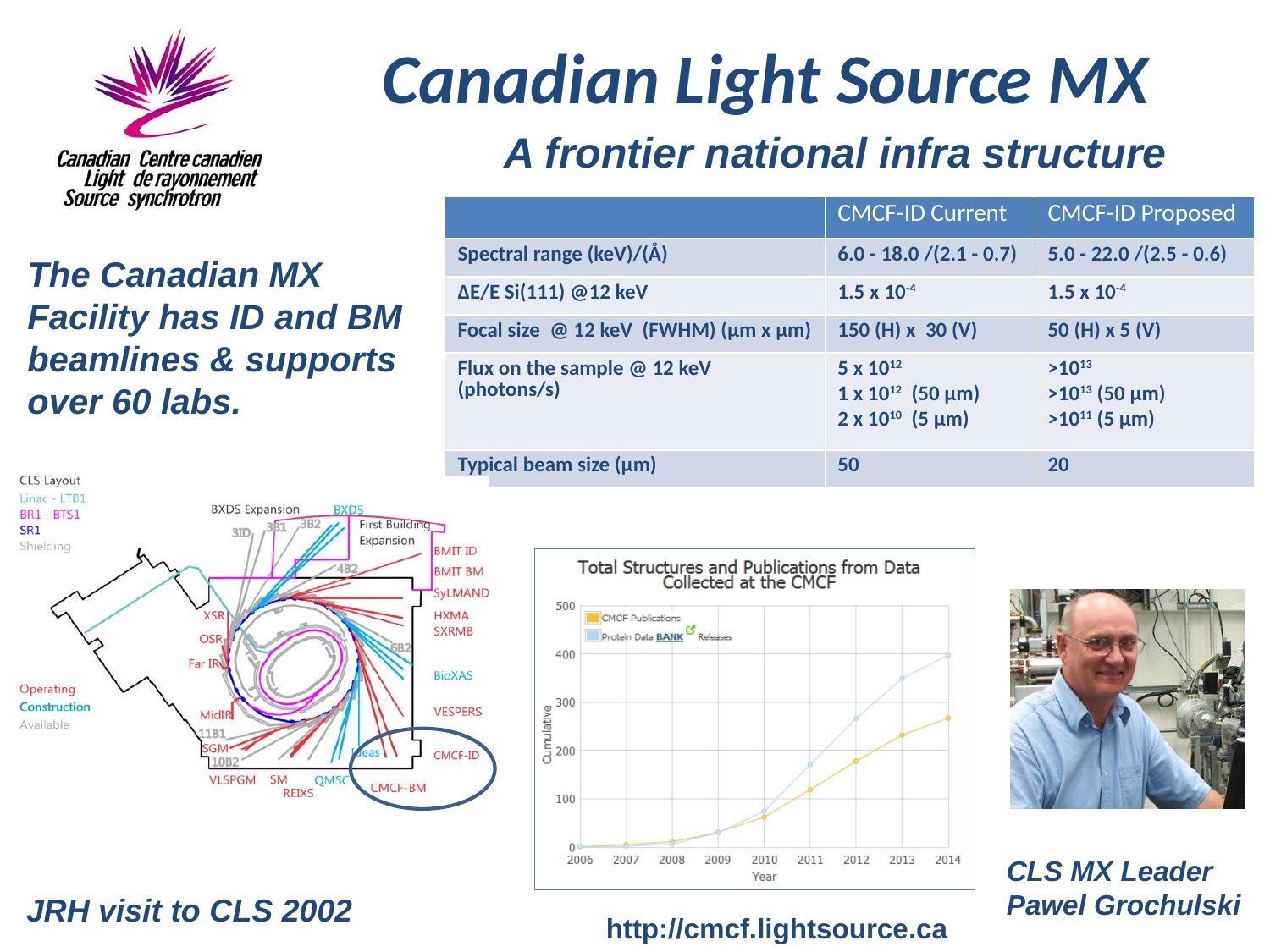

# Canadian Light Source MX
A frontier national infra structure
| | CMCF-ID Current | CMCF-ID Proposed |
| --- | --- | --- |
| Spectral range (keV)/(Å) | 6.0 - 18.0 /(2.1 - 0.7) | 5.0 - 22.0 /(2.5 - 0.6) |
| ΔE/E Si(111) @12 keV | 1.5 x 10^-4^ | 1.5 x 10^-4^ |
| Focal size @ 12 keV (FWHM) (μm x μm) | 150 (H) x 30 (V) | 50 (H) x 5 (V) |
| Flux on the sample @ 12 keV (photons/s) | 5 x 10^12^ 1 x 10^12^ (50 µm) 2 x 10^10^ (5 µm) | >1013 >1013 (50 µm) >1011 (5 µm) |
| Typical beam size (µm) | 50 | 20 |
The Canadian MX Facility has ID and BM beamlines & supports over 60 labs.
CLS MX Leader
Pawel Grochulski
JRH visit to CLS 2002
http://cmcf.lightsource.ca

## Slide 4
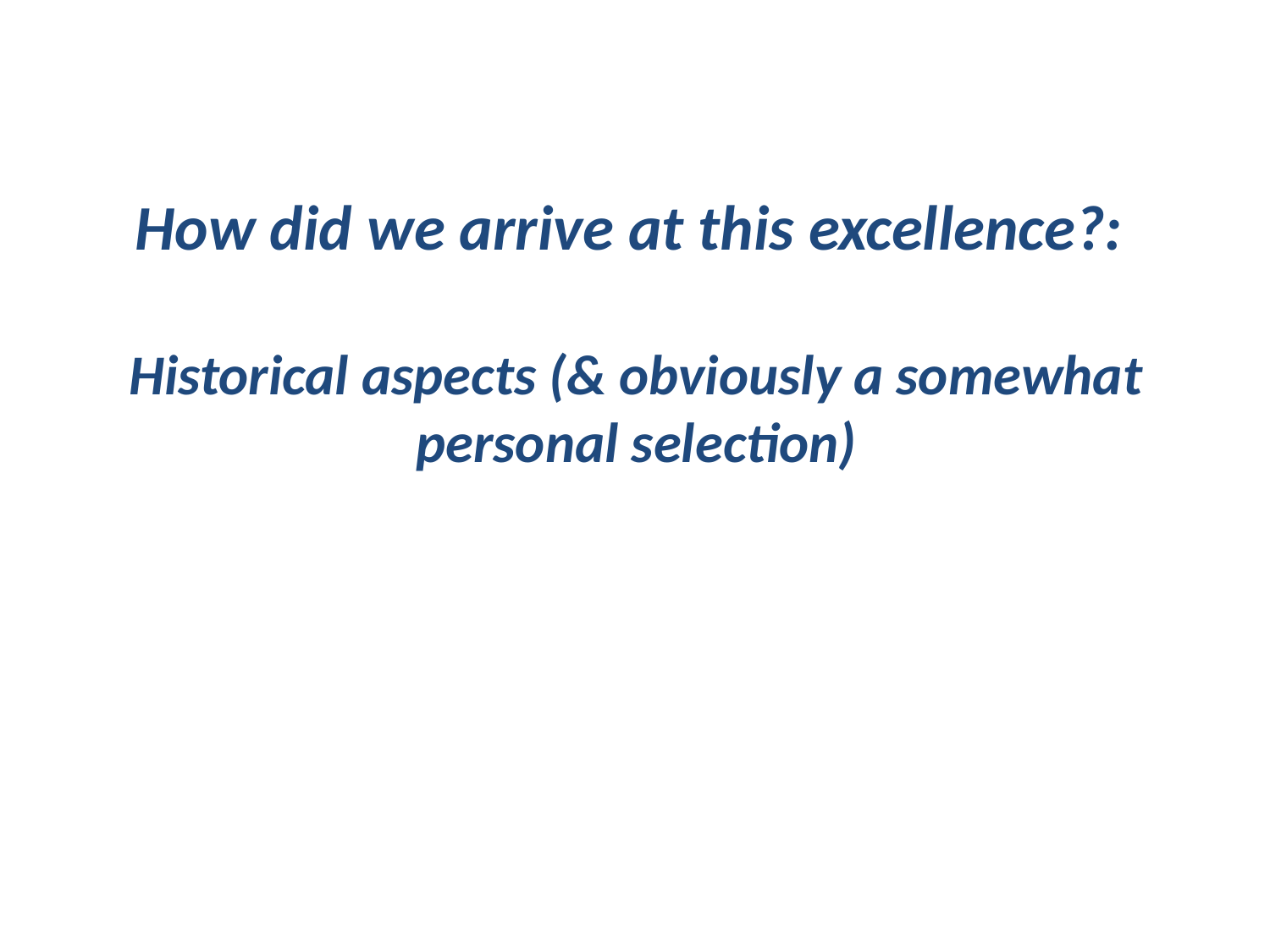

# How did we arrive at this excellence?: Historical aspects (& obviously a somewhat personal selection)

## Slide 5
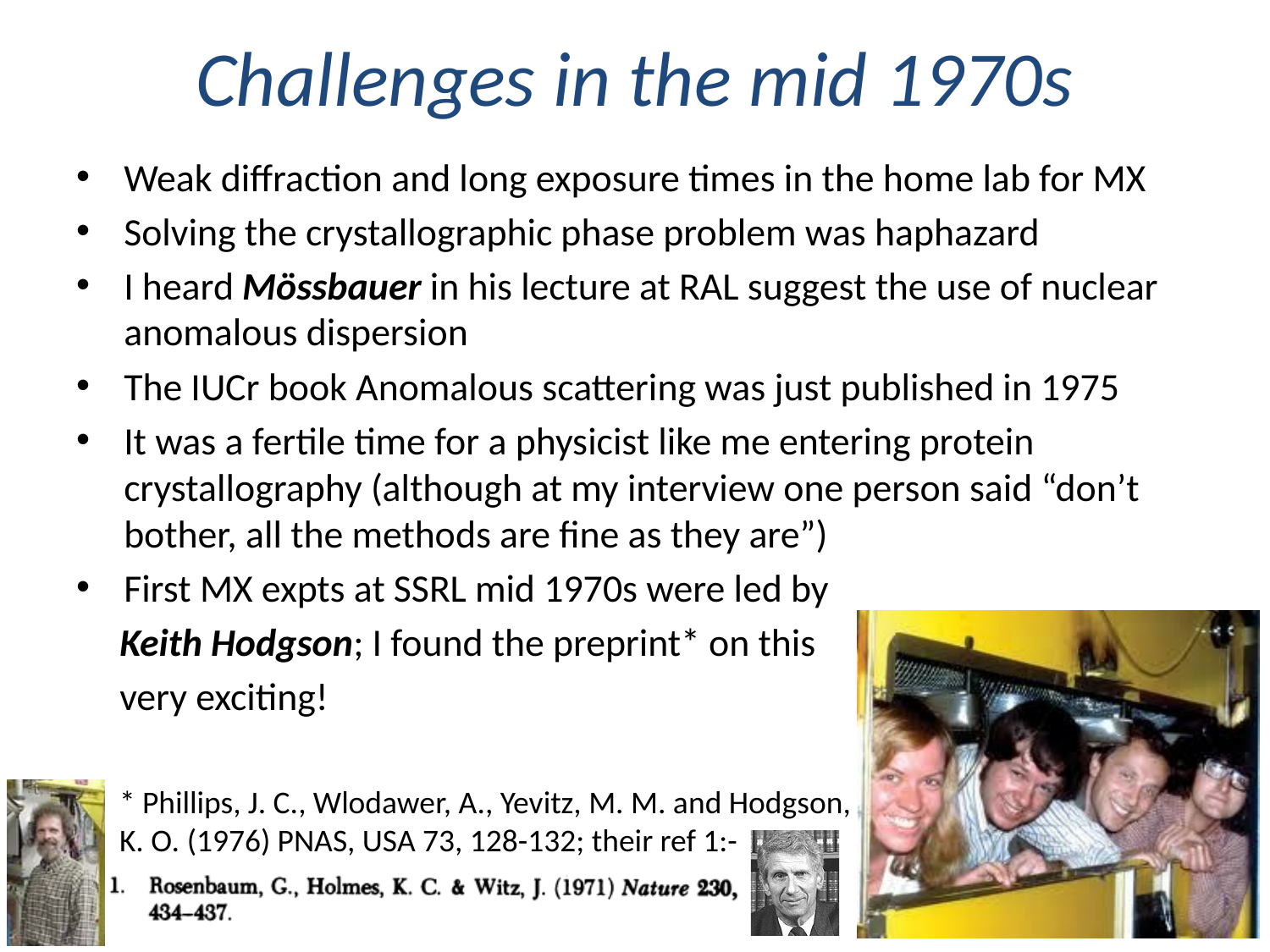

# Challenges in the mid 1970s
Weak diffraction and long exposure times in the home lab for MX
Solving the crystallographic phase problem was haphazard
I heard Mössbauer in his lecture at RAL suggest the use of nuclear anomalous dispersion
The IUCr book Anomalous scattering was just published in 1975
It was a fertile time for a physicist like me entering protein crystallography (although at my interview one person said “don’t bother, all the methods are fine as they are”)
First MX expts at SSRL mid 1970s were led by
 Keith Hodgson; I found the preprint* on this
 very exciting!
* Phillips, J. C., Wlodawer, A., Yevitz, M. M. and Hodgson,
K. O. (1976) PNAS, USA 73, 128-132; their ref 1:-

## Slide 6
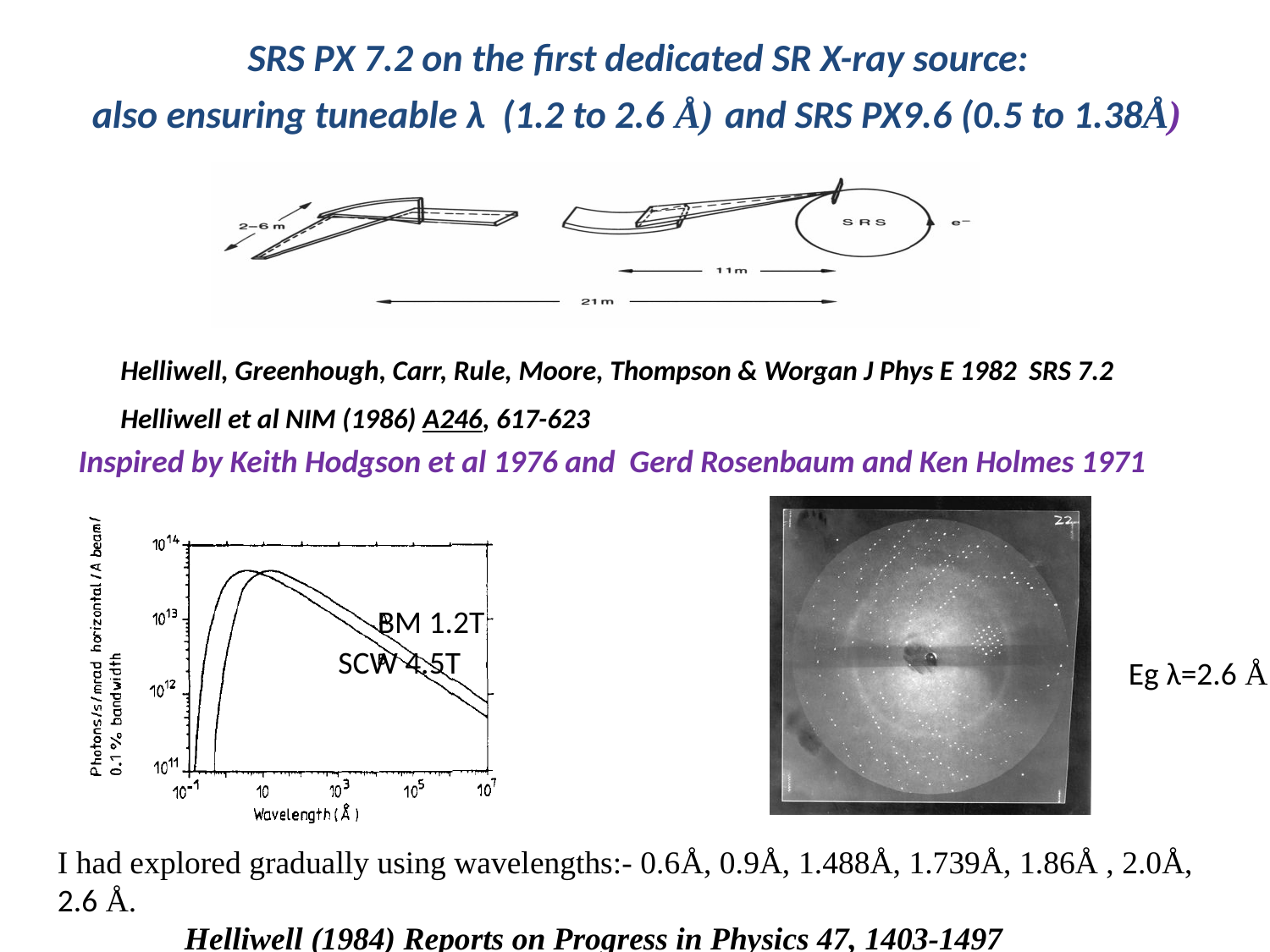

# SRS PX 7.2 on the first dedicated SR X-ray source: also ensuring tuneable λ (1.2 to 2.6 Å) and SRS PX9.6 (0.5 to 1.38Å)
Helliwell, Greenhough, Carr, Rule, Moore, Thompson & Worgan J Phys E 1982 SRS 7.2
Helliwell et al NIM (1986) A246, 617-623
Inspired by Keith Hodgson et al 1976 and Gerd Rosenbaum and Ken Holmes 1971
BM 1.2T
SCW 4.5T
Eg λ=2.6 Å
I had explored gradually using wavelengths:- 0.6Å, 0.9Å, 1.488Å, 1.739Å, 1.86Å , 2.0Å, 2.6 Å.
	Helliwell (1984) Reports on Progress in Physics 47, 1403-1497
I had explored gradually longer wavelengths:- 1.488Å, 1.739Å, 1.86Å , 2.0Å ...
[Helliwell (1984) Reports on Progress in Physics 47, 1403-1497. ]

## Slide 7
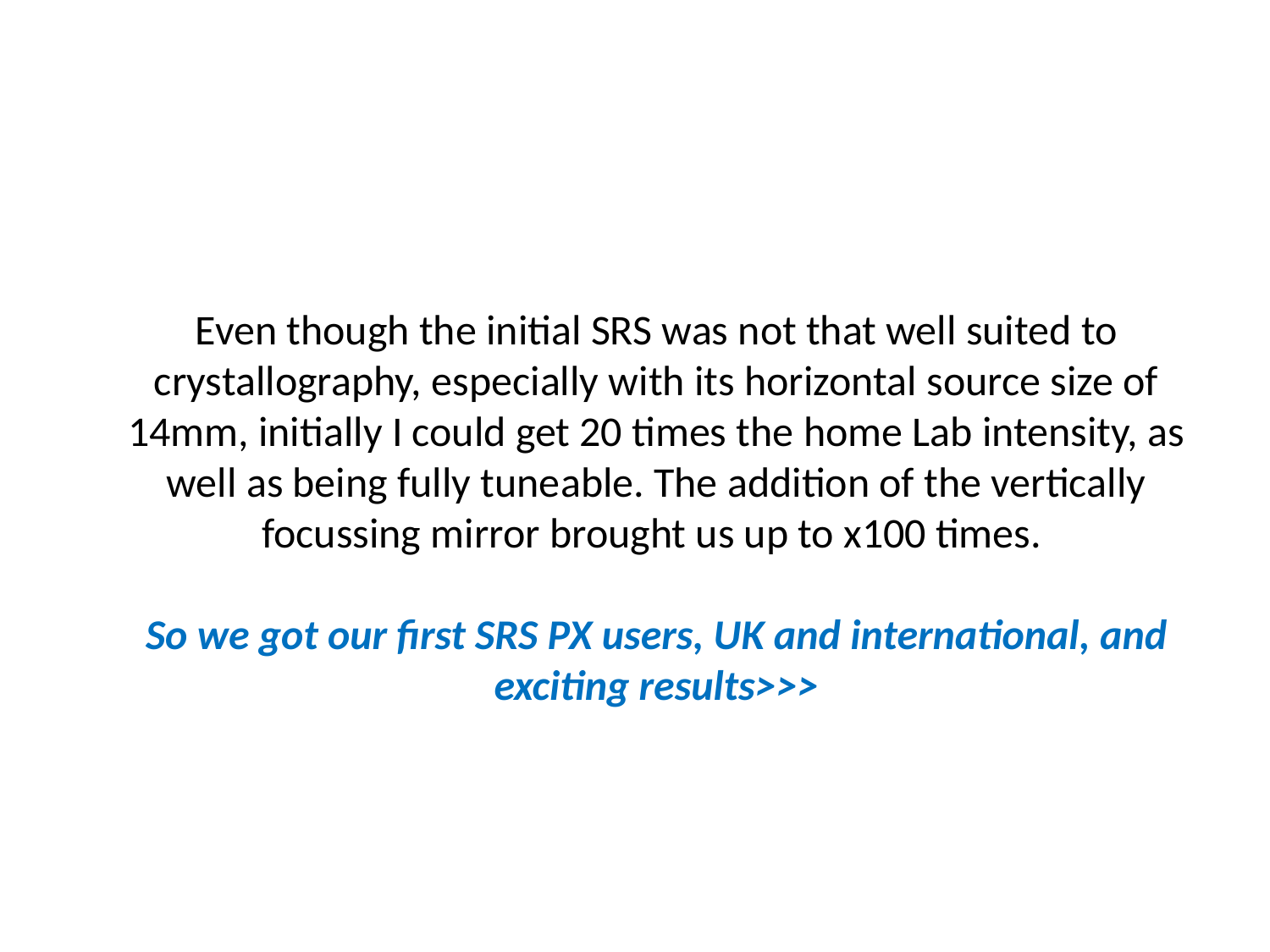

# Even though the initial SRS was not that well suited to crystallography, especially with its horizontal source size of 14mm, initially I could get 20 times the home Lab intensity, as well as being fully tuneable. The addition of the vertically focussing mirror brought us up to x100 times. So we got our first SRS PX users, UK and international, and exciting results>>>

## Slide 8
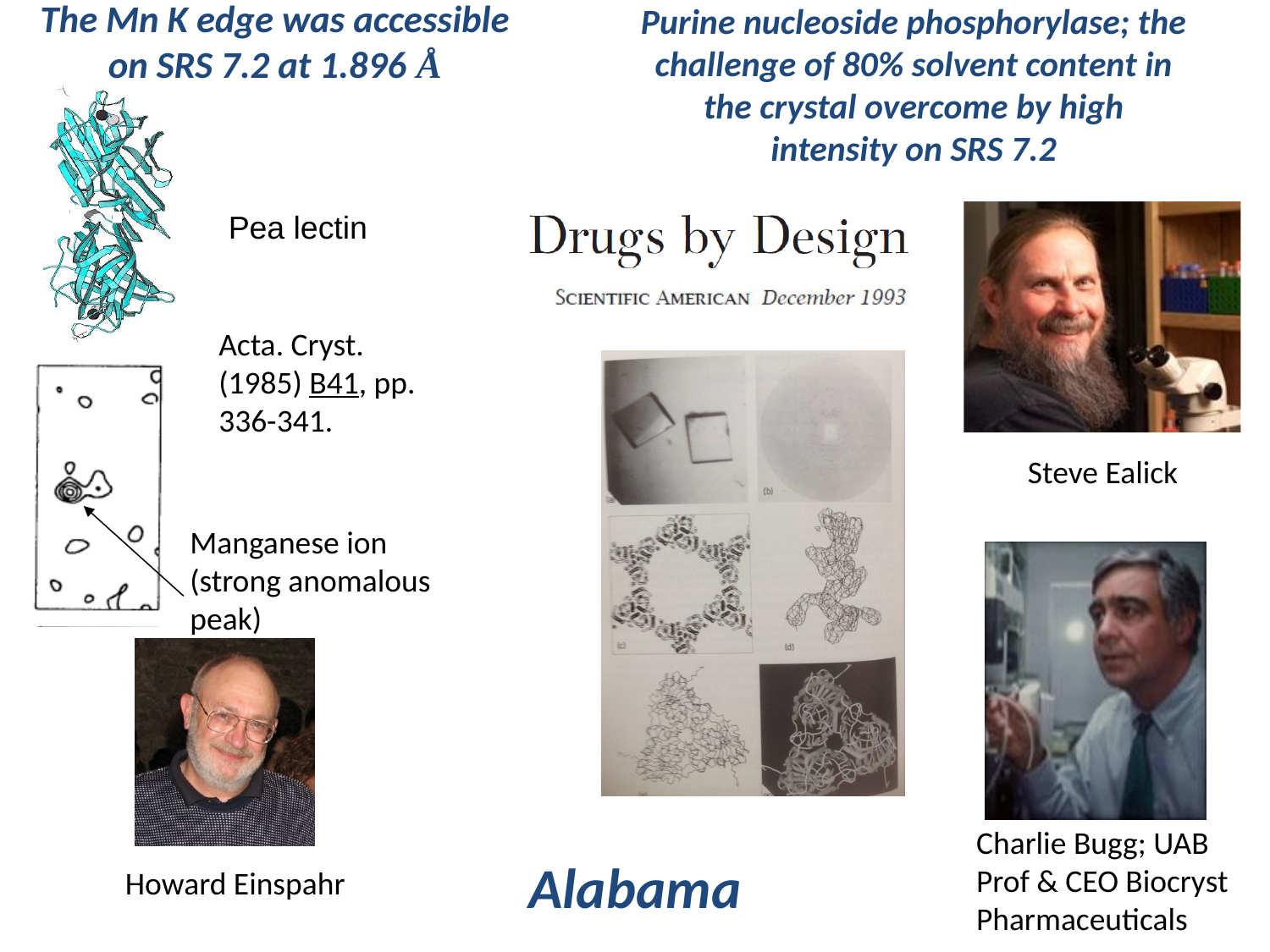

The Mn K edge was accessible on SRS 7.2 at 1.896 Å
Purine nucleoside phosphorylase; the challenge of 80% solvent content in the crystal overcome by high intensity on SRS 7.2
Pea lectin
# Acta. Cryst. (1985) B41, pp. 336-341.
Steve Ealick
Manganese ion
(strong anomalous
peak)
Charlie Bugg; UAB Prof & CEO Biocryst Pharmaceuticals
Alabama
Howard Einspahr

## Slide 9
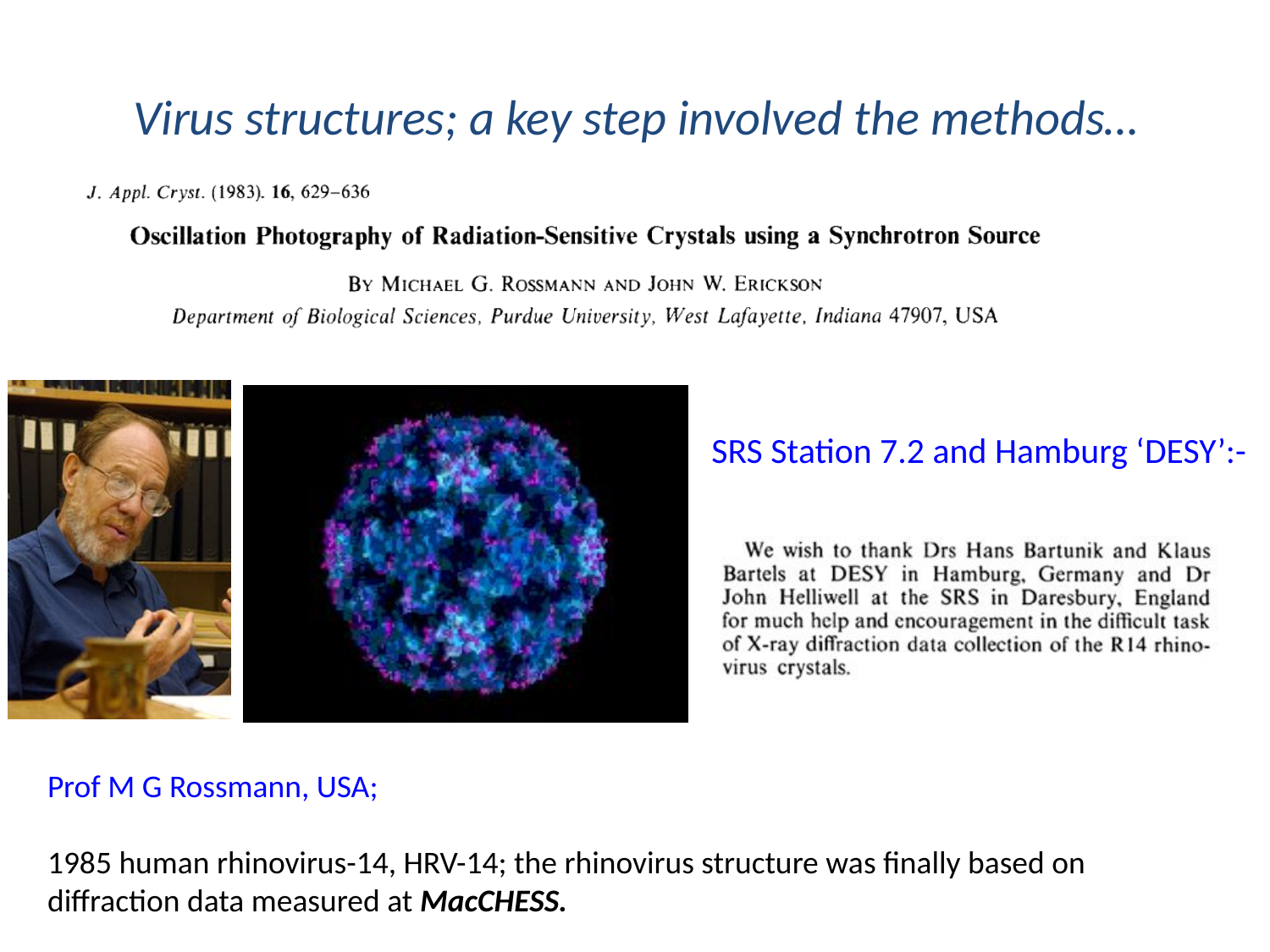

# Virus structures; a key step involved the methods…
SRS Station 7.2 and Hamburg ‘DESY’:-
Prof M G Rossmann, USA;
1985 human rhinovirus-14, HRV-14; the rhinovirus structure was finally based on diffraction data measured at MacCHESS.

## Slide 10
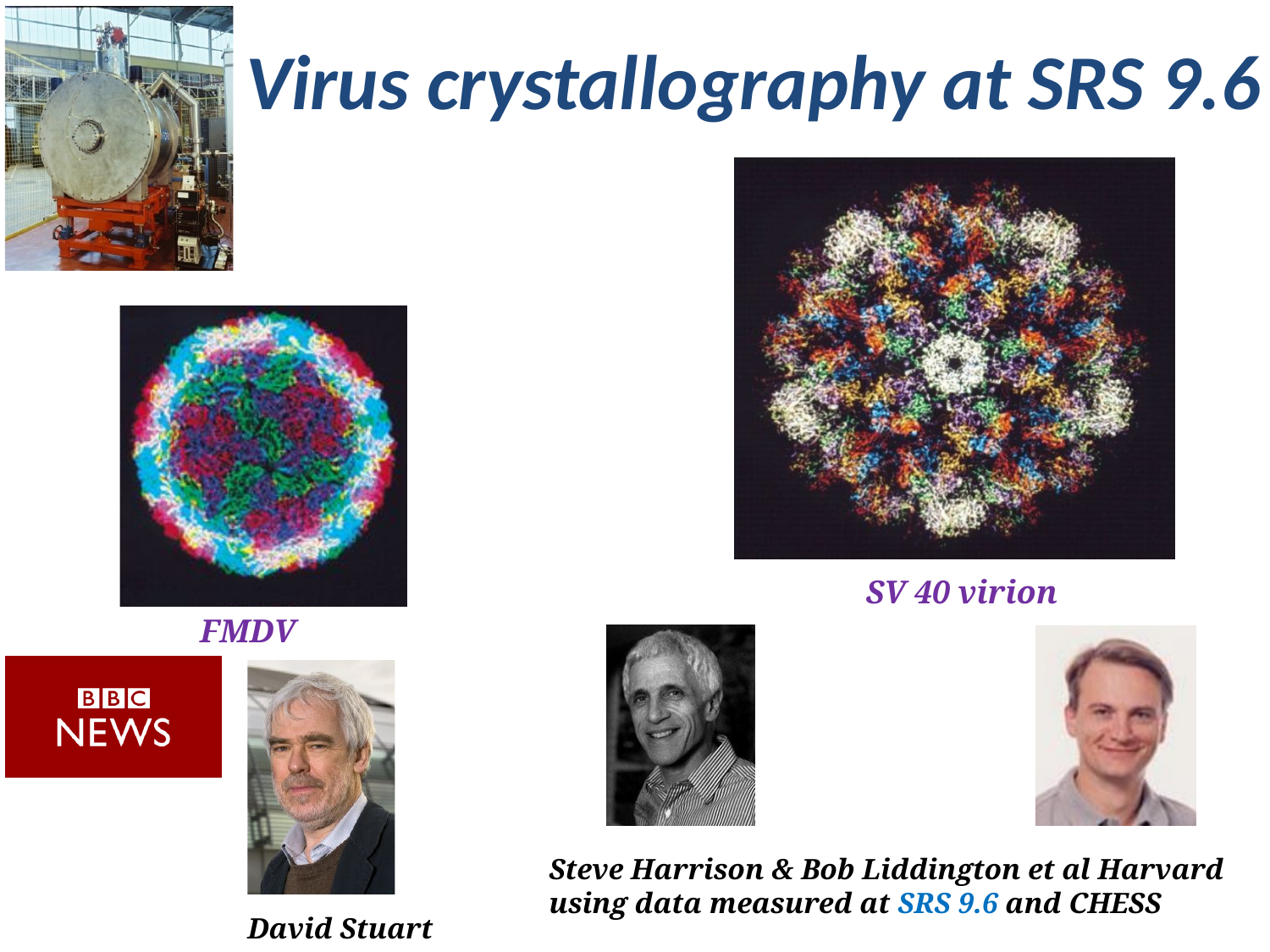

# Virus crystallography at SRS 9.6
SV 40 virion
FMDV
Steve Harrison & Bob Liddington et al Harvard
using data measured at SRS 9.6 and CHESS
David Stuart

## Slide 11
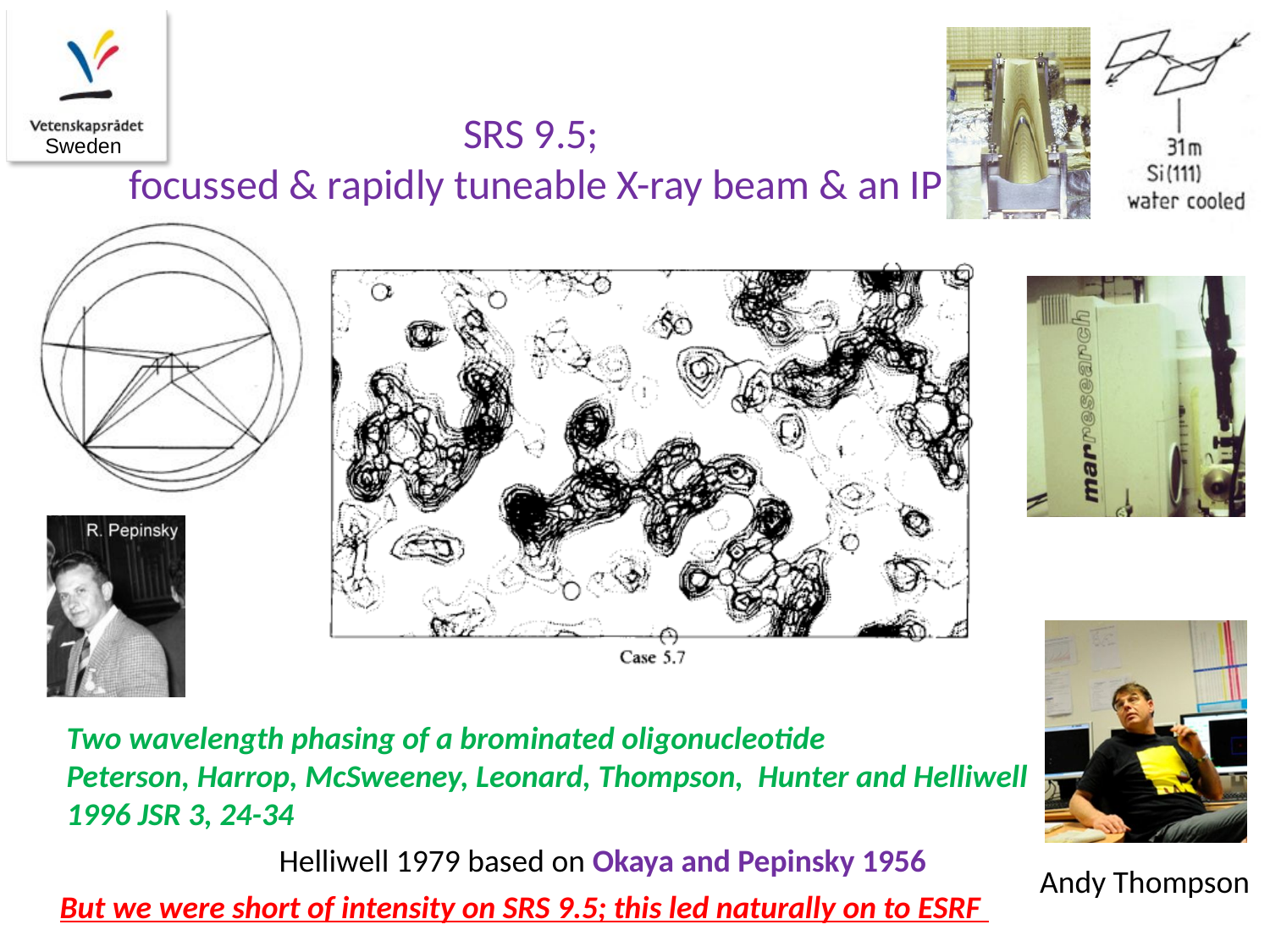

# SRS 9.5; focussed & rapidly tuneable X-ray beam & an IP
Sweden
Two wavelength phasing of a brominated oligonucleotide
Peterson, Harrop, McSweeney, Leonard, Thompson, Hunter and Helliwell
1996 JSR 3, 24-34
Helliwell 1979 based on Okaya and Pepinsky 1956
Andy Thompson
But we were short of intensity on SRS 9.5; this led naturally on to ESRF

## Slide 12
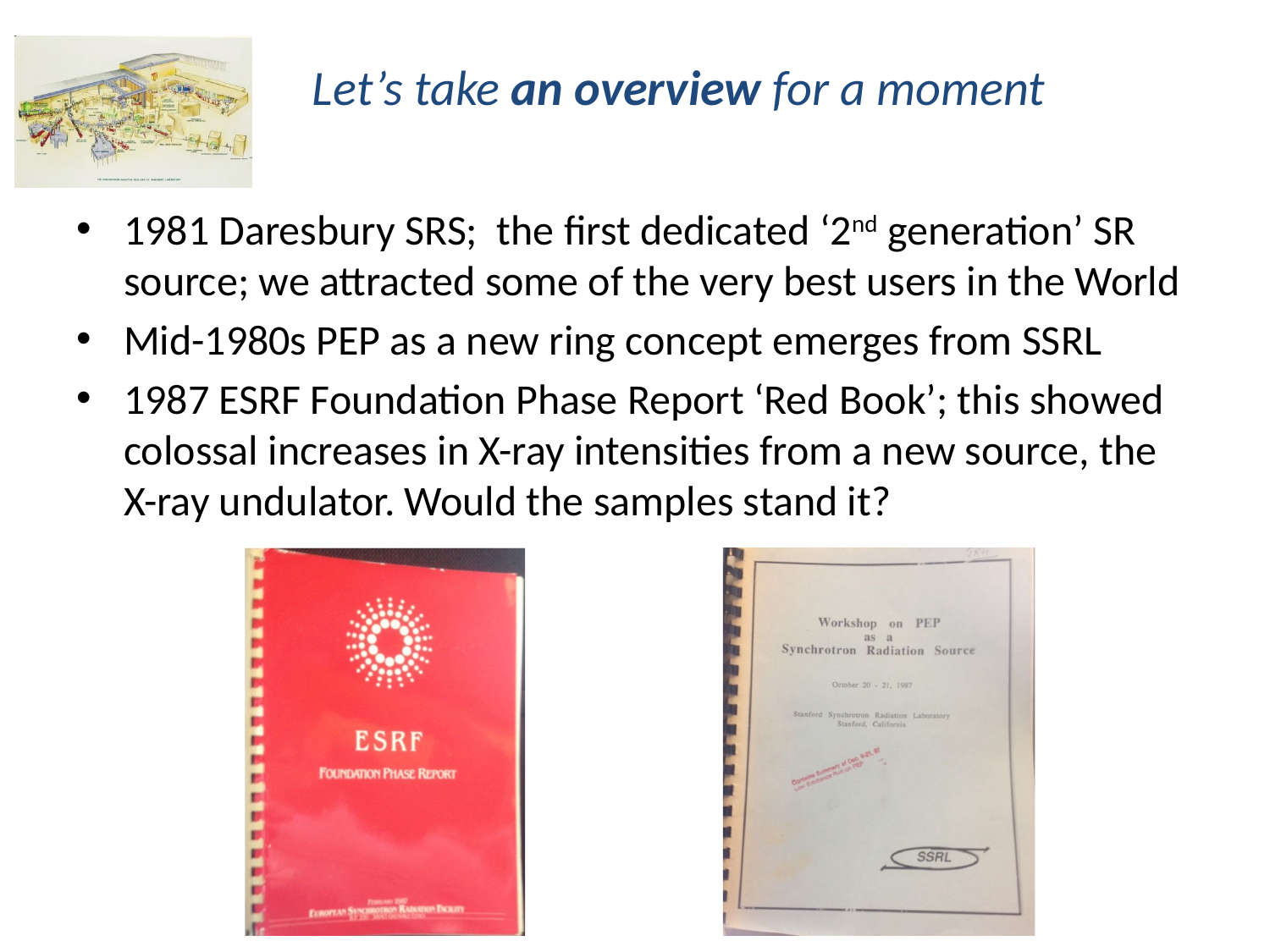

# Let’s take an overview for a moment
1981 Daresbury SRS; the first dedicated ‘2nd generation’ SR source; we attracted some of the very best users in the World
Mid-1980s PEP as a new ring concept emerges from SSRL
1987 ESRF Foundation Phase Report ‘Red Book’; this showed colossal increases in X-ray intensities from a new source, the X-ray undulator. Would the samples stand it?

## Slide 13
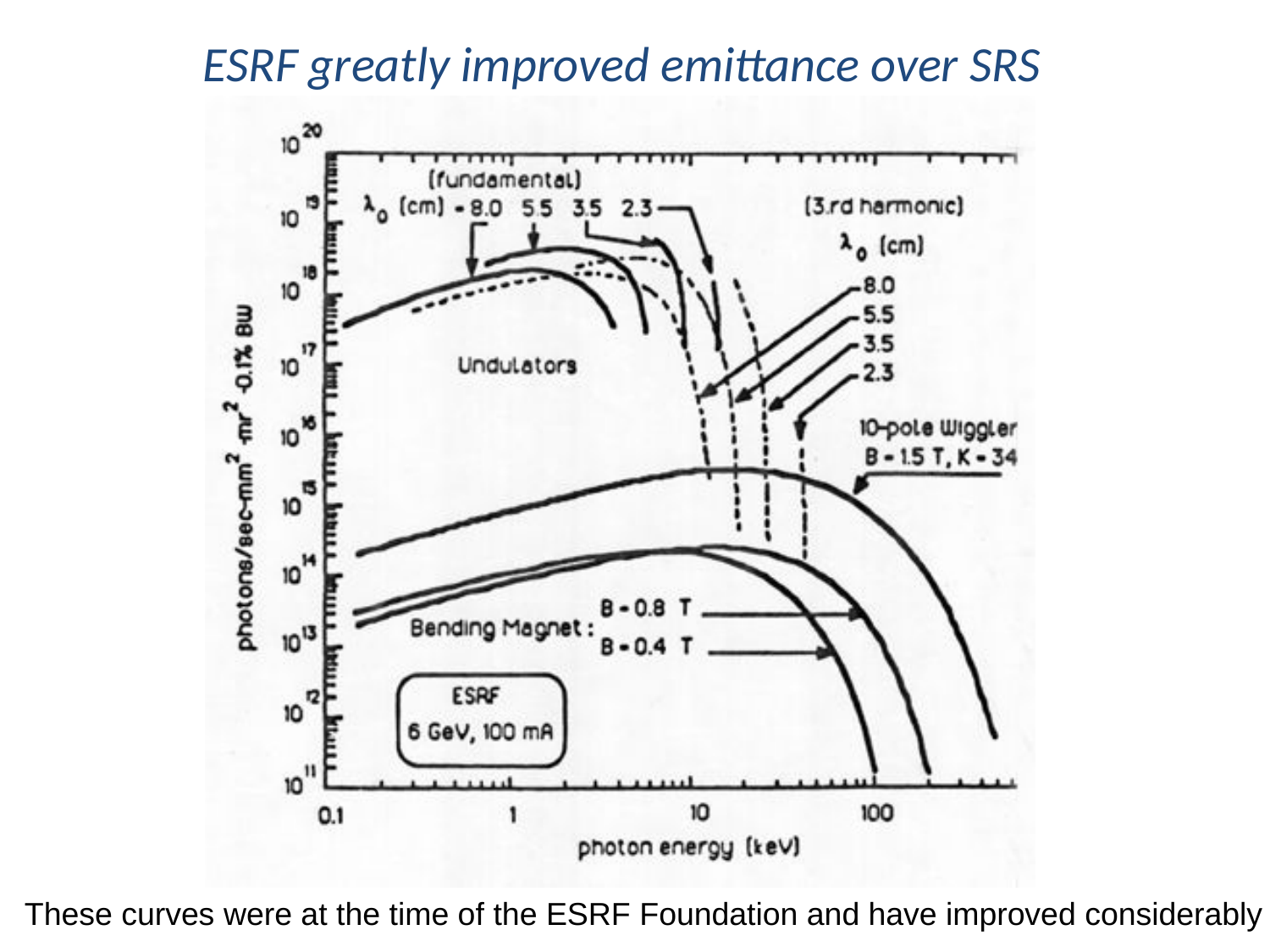

ESRF greatly improved emittance over SRS
These curves were at the time of the ESRF Foundation and have improved considerably

## Slide 14
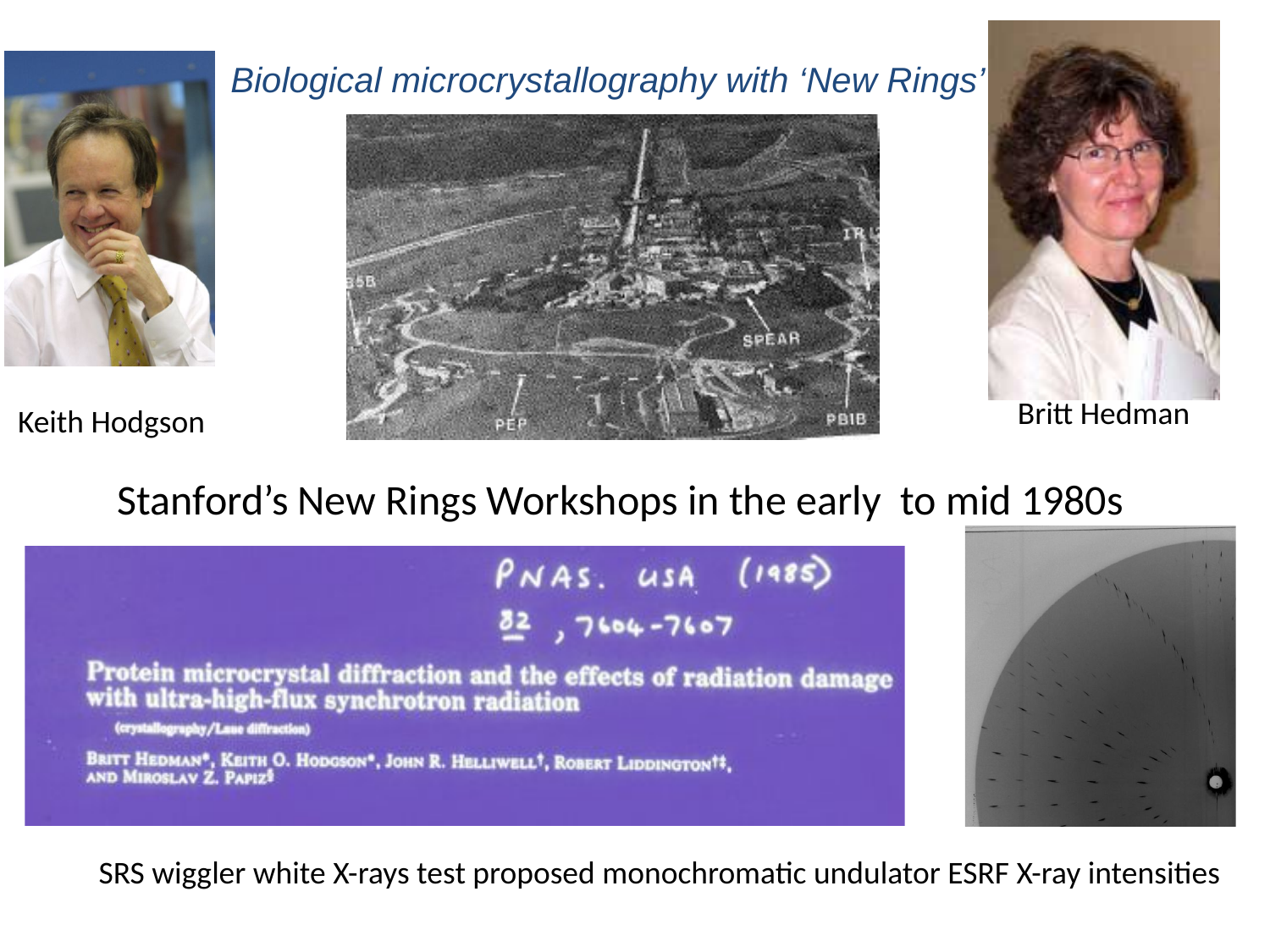

Biological microcrystallography with ‘New Rings’
Britt Hedman
Keith Hodgson
Stanford’s New Rings Workshops in the early to mid 1980s
SRS wiggler white X-rays test proposed monochromatic undulator ESRF X-ray intensities

## Slide 15
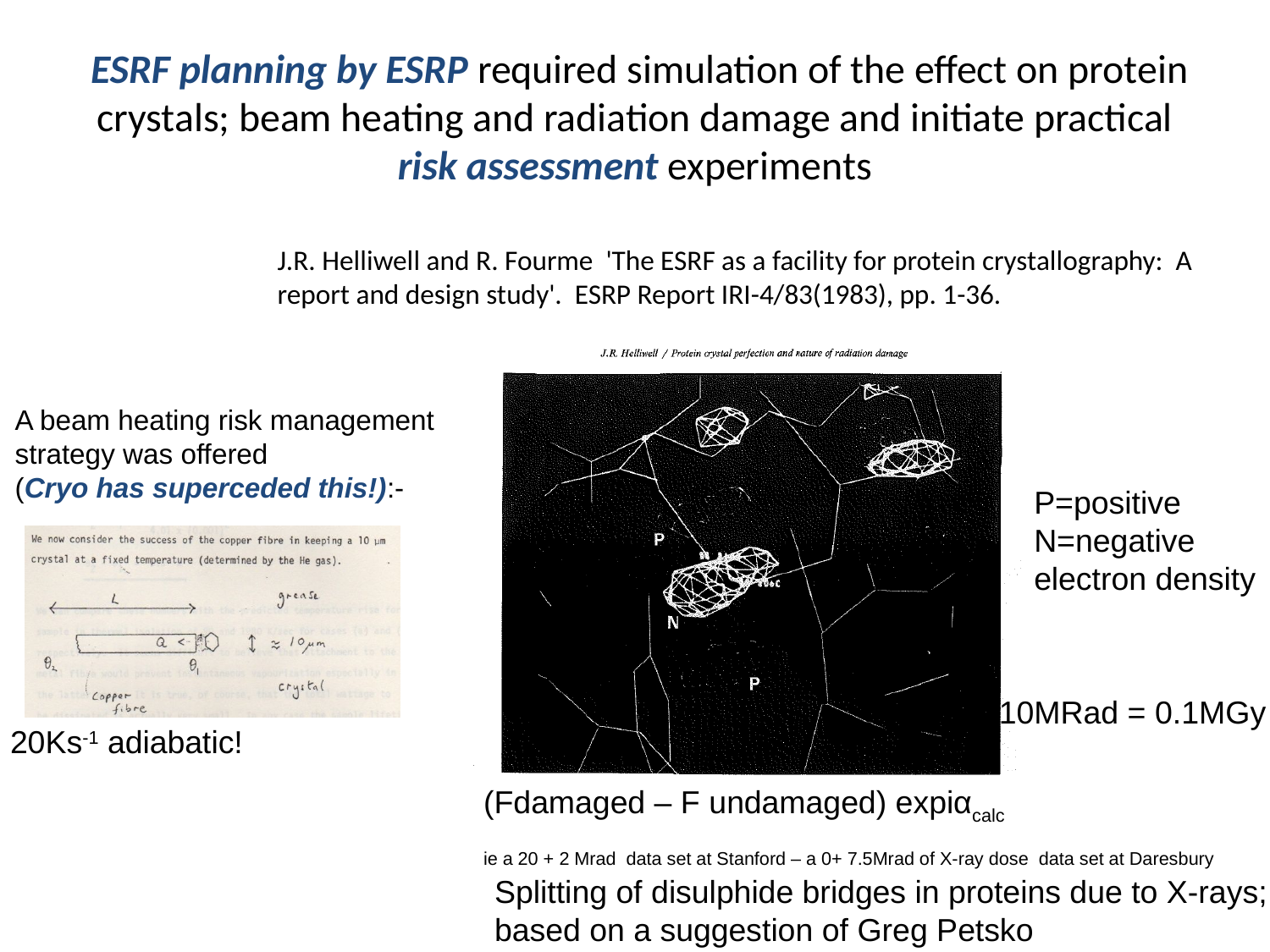

# ESRF planning by ESRP required simulation of the effect on protein crystals; beam heating and radiation damage and initiate practical risk assessment experiments
J.R. Helliwell and R. Fourme 'The ESRF as a facility for protein crystallography: A report and design study'. ESRP Report IRI-4/83(1983), pp. 1-36.
A beam heating risk management
strategy was offered
(Cryo has superceded this!):-
P=positive
N=negative
electron density
10MRad = 0.1MGy
20Ks-1 adiabatic!
(Fdamaged – F undamaged) expiαcalc
ie a 20 + 2 Mrad data set at Stanford – a 0+ 7.5Mrad of X-ray dose data set at Daresbury
Splitting of disulphide bridges in proteins due to X-rays;
based on a suggestion of Greg Petsko

## Slide 16
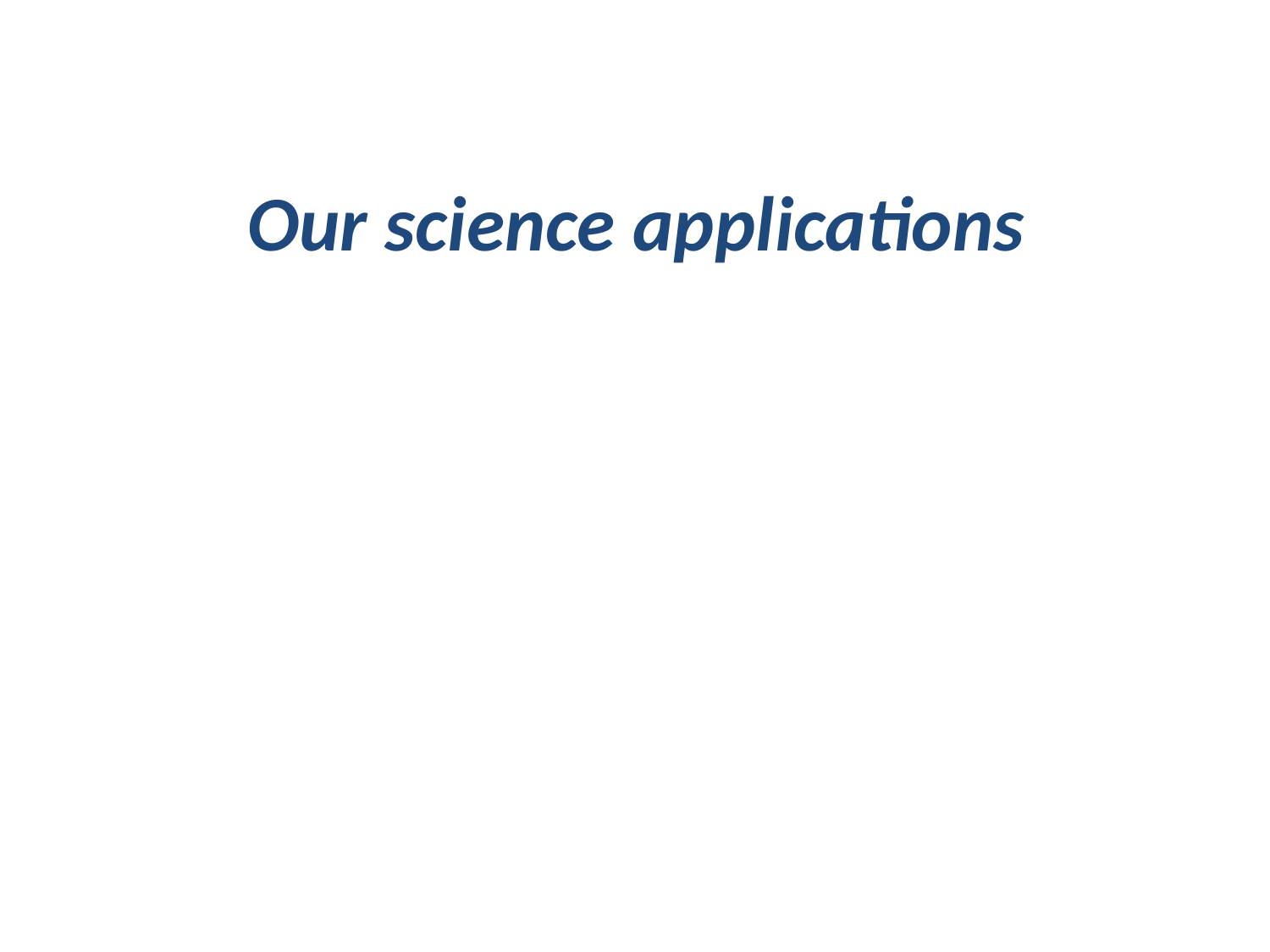

# Our science applications

## Slide 17
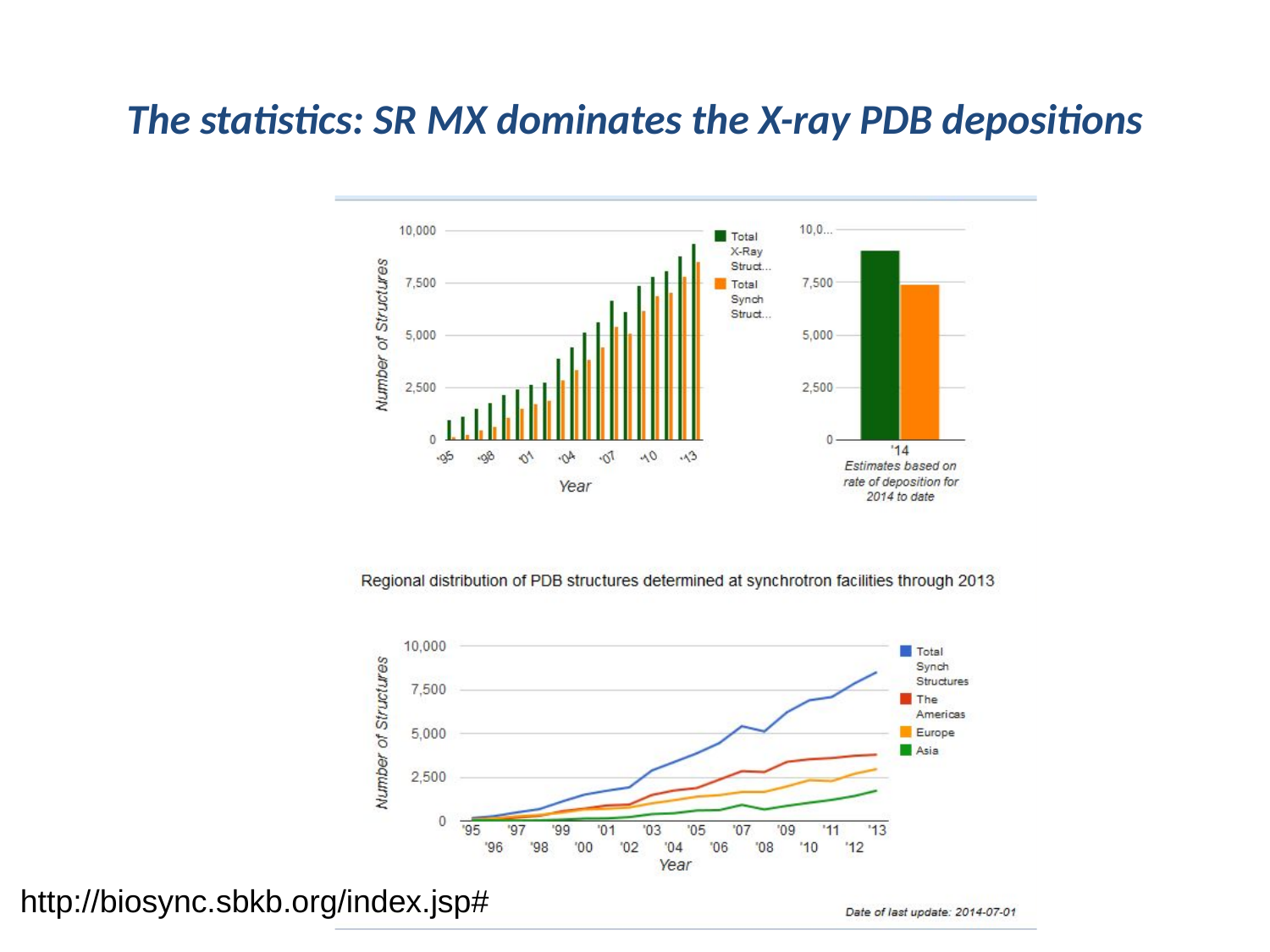

# The statistics: SR MX dominates the X-ray PDB depositions
http://biosync.sbkb.org/index.jsp#

## Slide 18
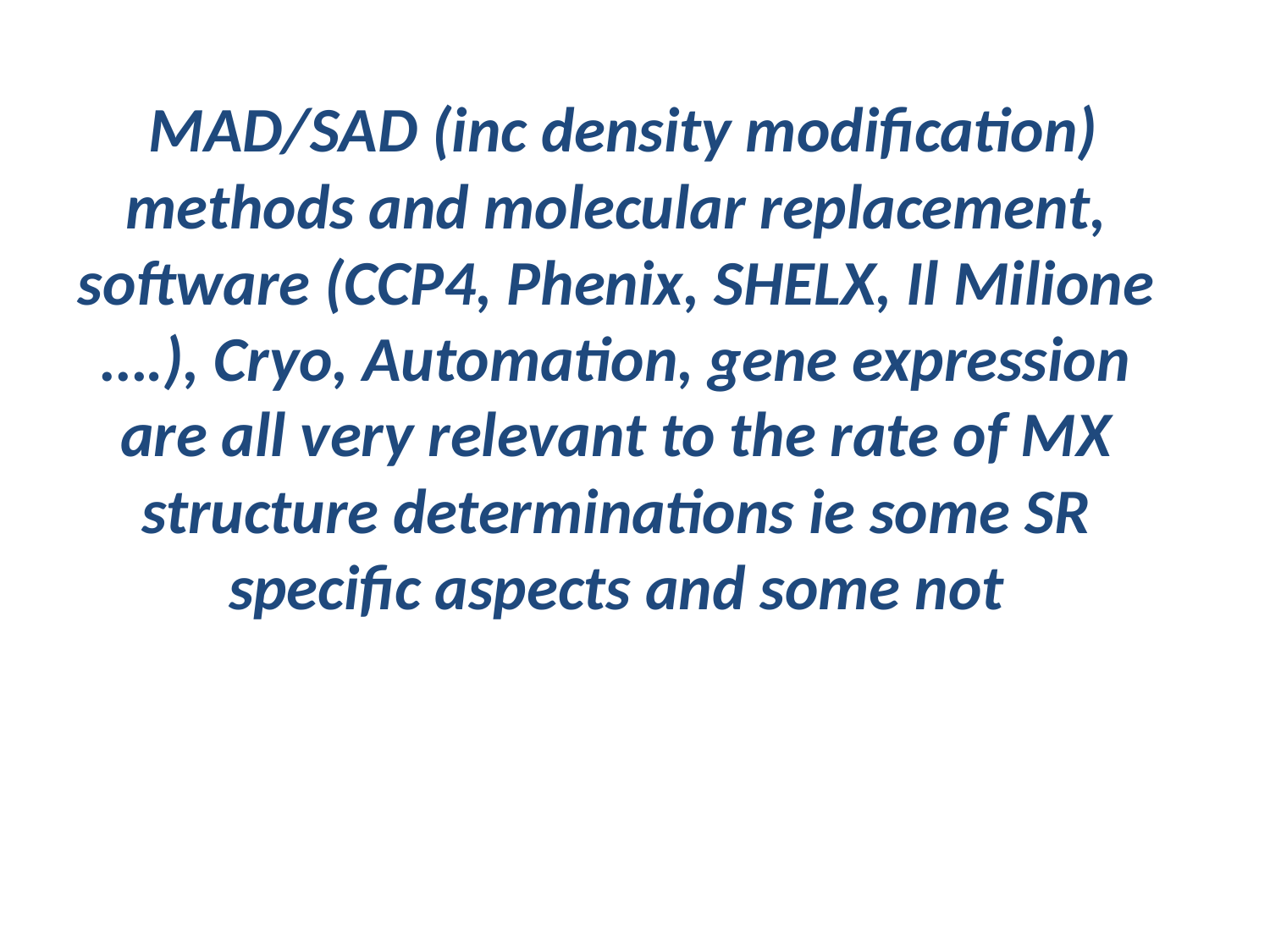

# MAD/SAD (inc density modification) methods and molecular replacement, software (CCP4, Phenix, SHELX, Il Milione ….), Cryo, Automation, gene expression are all very relevant to the rate of MX structure determinations ie some SR specific aspects and some not

## Slide 19
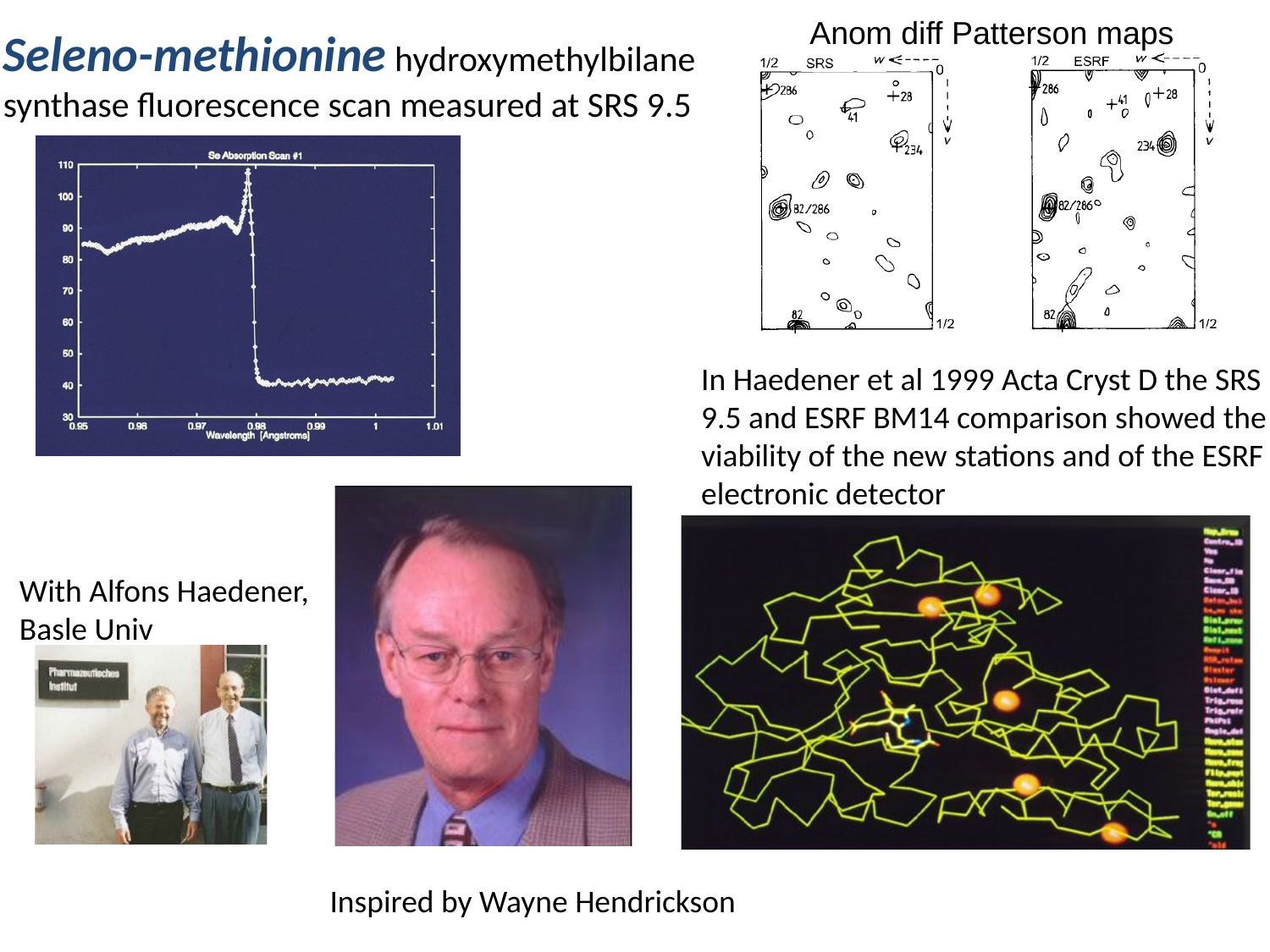

Anom diff Patterson maps
Seleno-methionine hydroxymethylbilane
synthase fluorescence scan measured at SRS 9.5
In Haedener et al 1999 Acta Cryst D the SRS 9.5 and ESRF BM14 comparison showed the viability of the new stations and of the ESRF electronic detector
With Alfons Haedener,
Basle Univ
Inspired by Wayne Hendrickson

## Slide 20
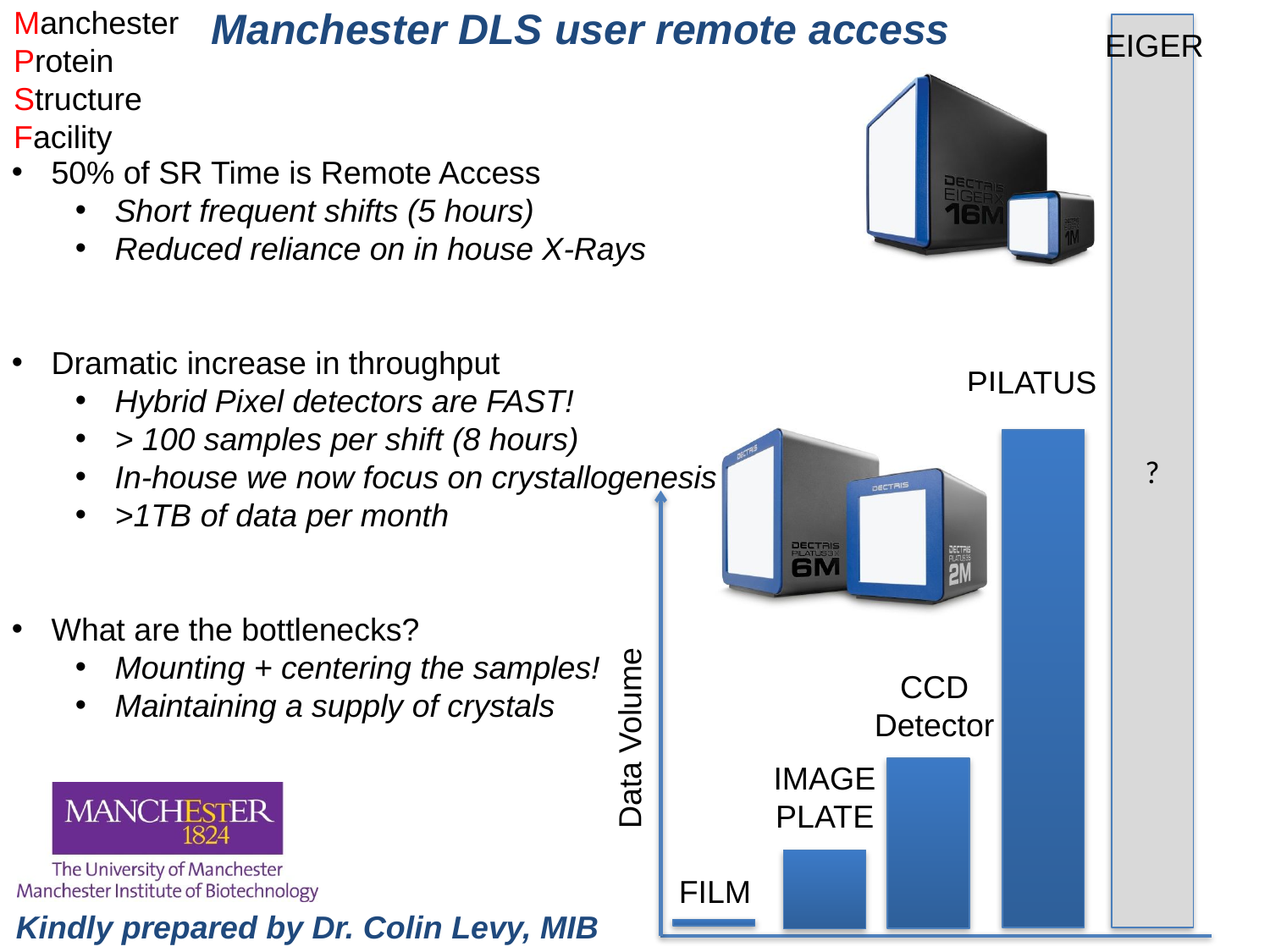

Manchester
Protein
Structure
Facility
Manchester DLS user remote access
?
EIGER
50% of SR Time is Remote Access
Short frequent shifts (5 hours)
Reduced reliance on in house X-Rays
Dramatic increase in throughput
Hybrid Pixel detectors are FAST!
> 100 samples per shift (8 hours)
In-house we now focus on crystallogenesis
>1TB of data per month
What are the bottlenecks?
Mounting + centering the samples!
Maintaining a supply of crystals
PILATUS
CCD
Detector
Data Volume
IMAGE
PLATE
FILM
Kindly prepared by Dr. Colin Levy, MIB

## Slide 21
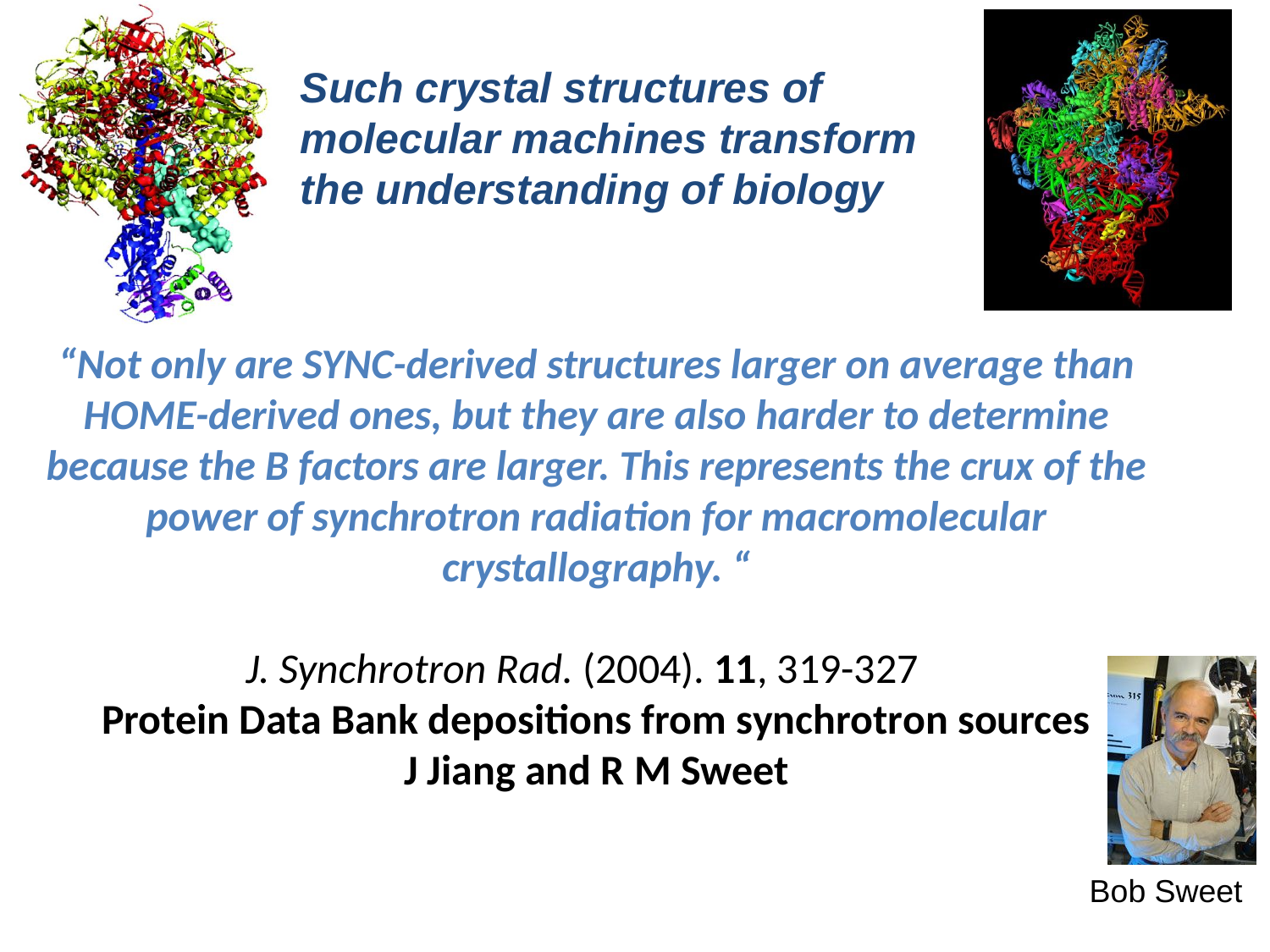

Such crystal structures of
molecular machines transform
the understanding of biology
# “Not only are SYNC-derived structures larger on average than HOME-derived ones, but they are also harder to determine because the B factors are larger. This represents the crux of the power of synchrotron radiation for macromolecular crystallography. “ J. Synchrotron Rad. (2004). 11, 319-327    Protein Data Bank depositions from synchrotron sources J Jiang and R M Sweet
Bob Sweet

## Slide 22
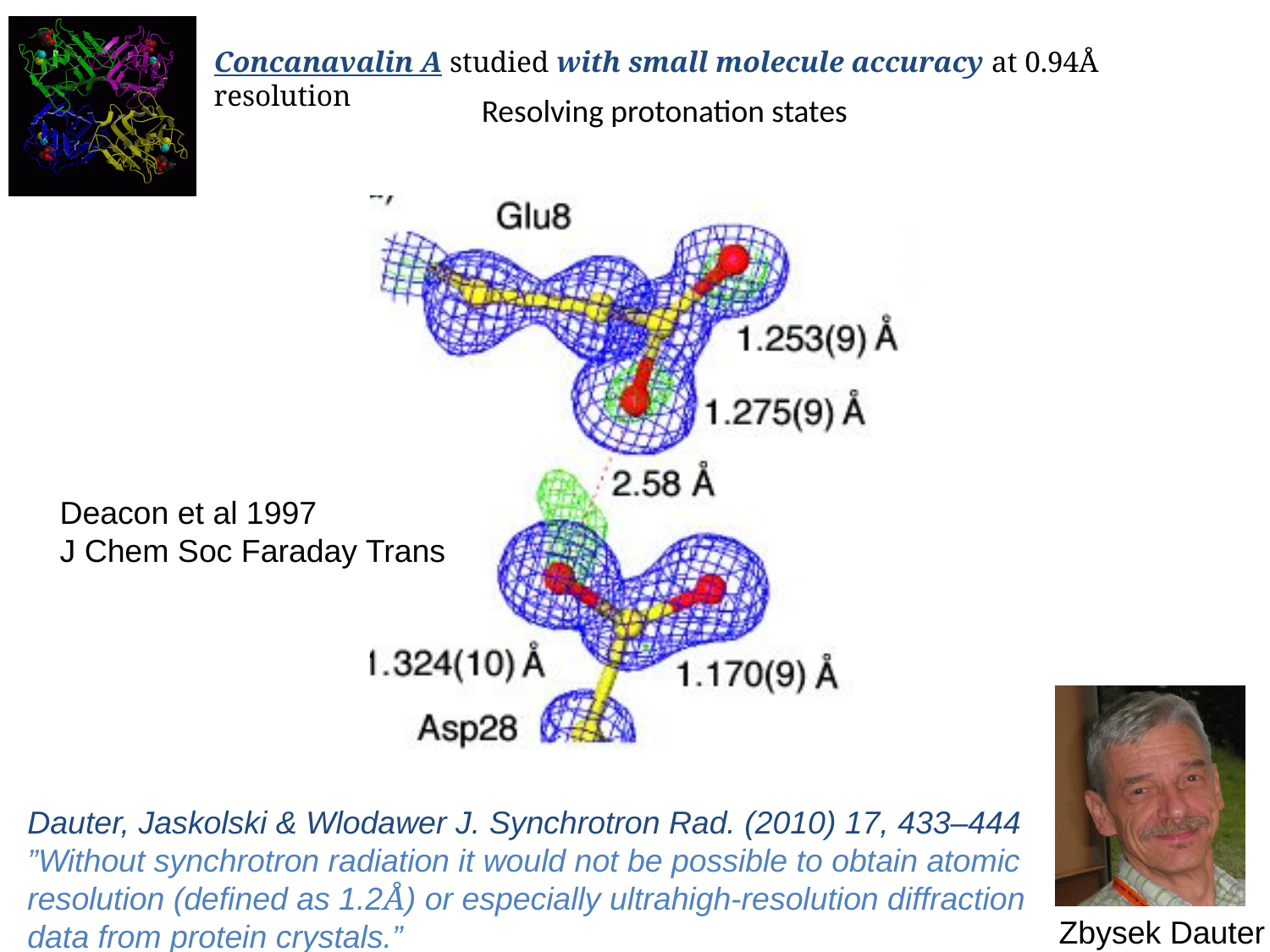

Concanavalin A studied with small molecule accuracy at 0.94Å resolution
Resolving protonation states
Deacon et al 1997
J Chem Soc Faraday Trans
Dauter, Jaskolski & Wlodawer J. Synchrotron Rad. (2010) 17, 433–444
”Without synchrotron radiation it would not be possible to obtain atomic resolution (defined as 1.2Å) or especially ultrahigh-resolution diffraction
data from protein crystals.”
Zbysek Dauter

## Slide 23
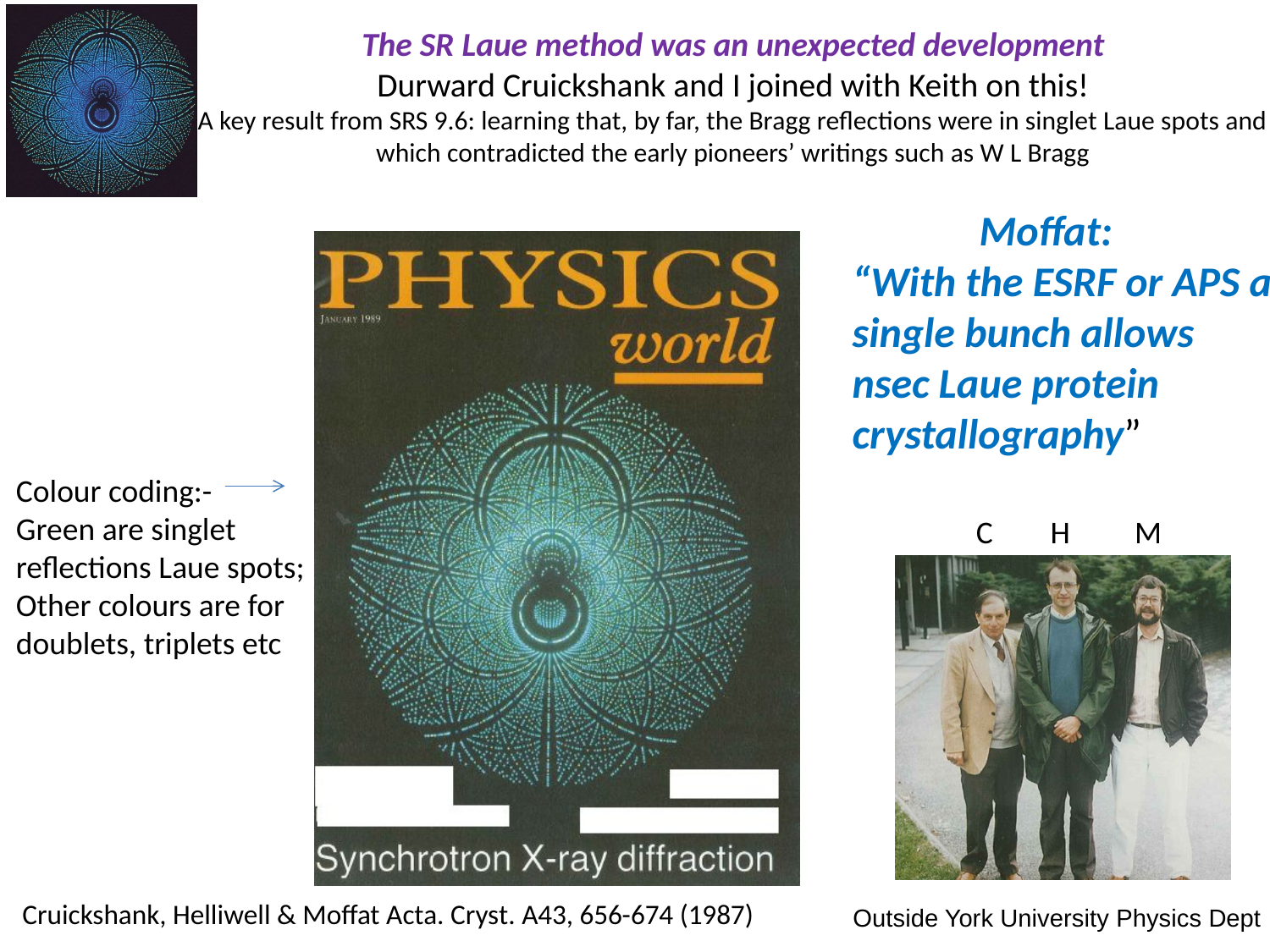

# The SR Laue method was an unexpected development Durward Cruickshank and I joined with Keith on this! A key result from SRS 9.6: learning that, by far, the Bragg reflections were in singlet Laue spots and which contradicted the early pioneers’ writings such as W L Bragg
	Moffat:
“With the ESRF or APS a single bunch allows nsec Laue protein crystallography”
Colour coding:-
Green are singlet
reflections Laue spots;
Other colours are for
doublets, triplets etc
C H M
Cruickshank, Helliwell & Moffat Acta. Cryst. A43, 656-674 (1987)
Outside York University Physics Dept

## Slide 24
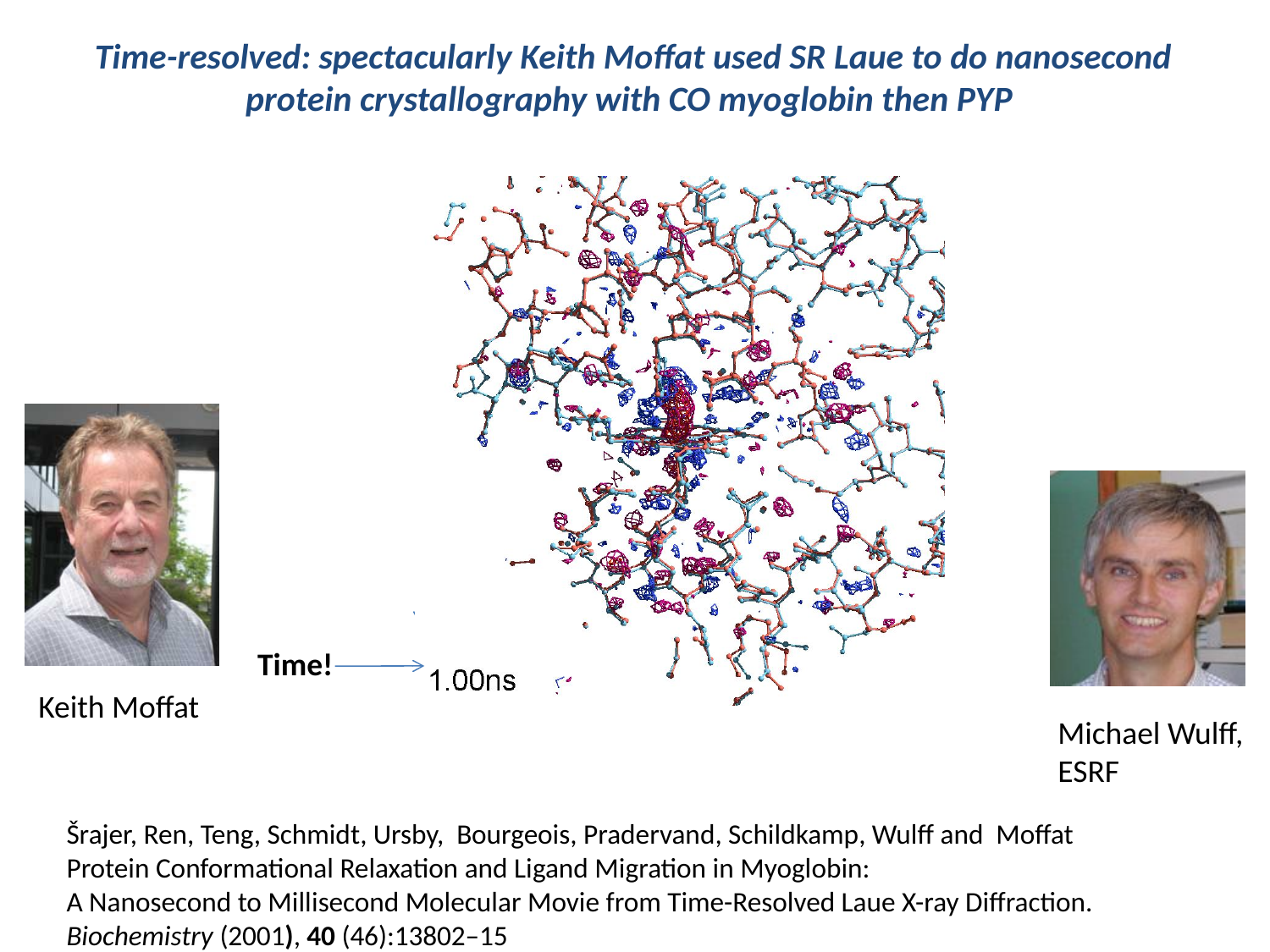

# Time-resolved: spectacularly Keith Moffat used SR Laue to do nanosecond protein crystallography with CO myoglobin then PYP
Time!
Keith Moffat
Michael Wulff,
ESRF
Šrajer, Ren, Teng, Schmidt, Ursby, Bourgeois, Pradervand, Schildkamp, Wulff and Moffat
Protein Conformational Relaxation and Ligand Migration in Myoglobin:
A Nanosecond to Millisecond Molecular Movie from Time-Resolved Laue X-ray Diffraction.
Biochemistry (2001), 40 (46):13802–15

## Slide 25
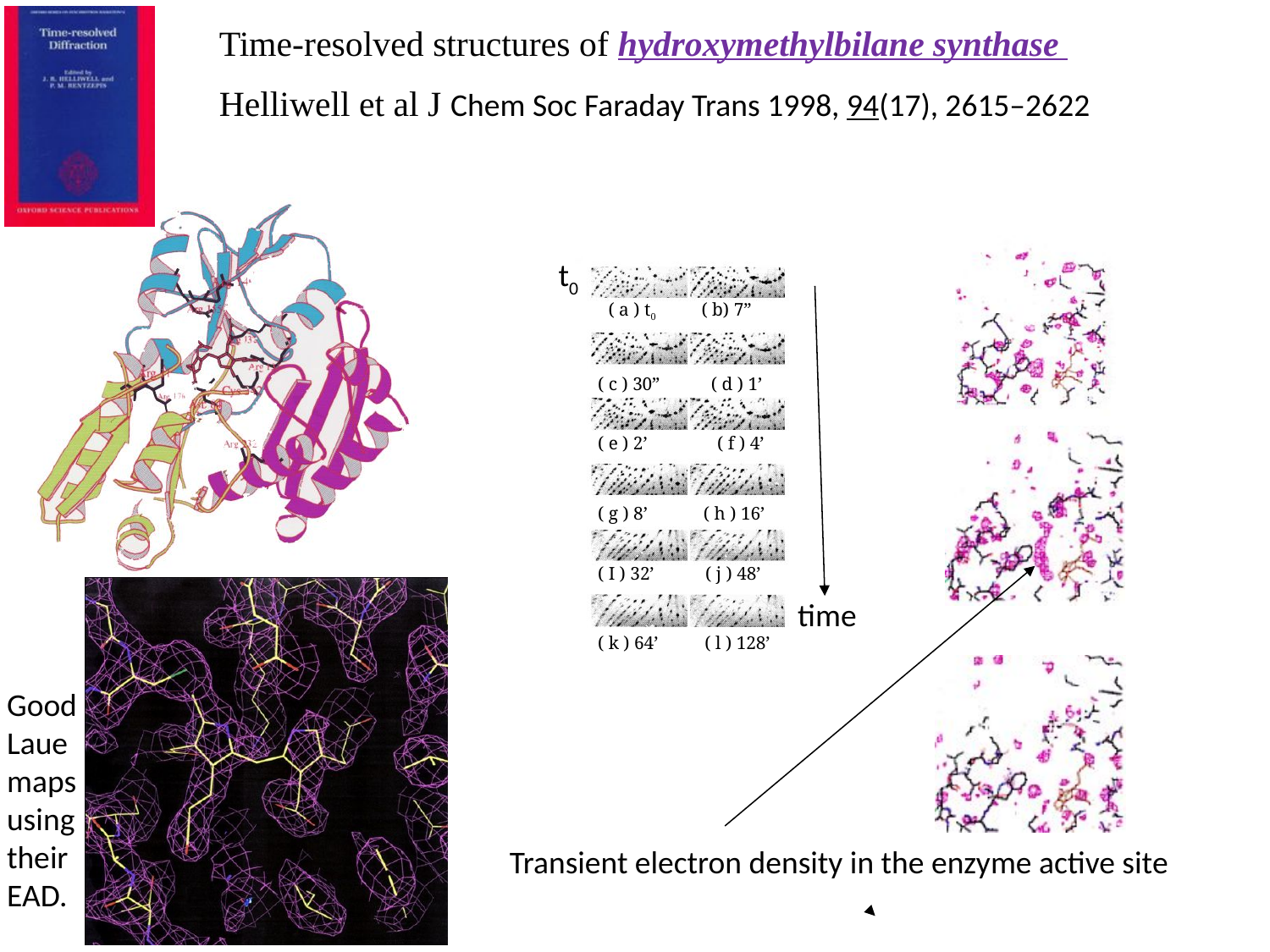

Time-resolved structures of hydroxymethylbilane synthase
	Helliwell et al J Chem Soc Faraday Trans 1998, 94(17), 2615–2622
t0
( a ) t0 ( b) 7”
( c ) 30” ( d ) 1’
( e ) 2’ ( f ) 4’
( g ) 8’ ( h ) 16’
( I ) 32’ ( j ) 48’
time
( k ) 64’ ( l ) 128’
 Good
 Laue
 maps
 using
 their
 EAD.
Transient electron density in the enzyme active site

## Slide 26
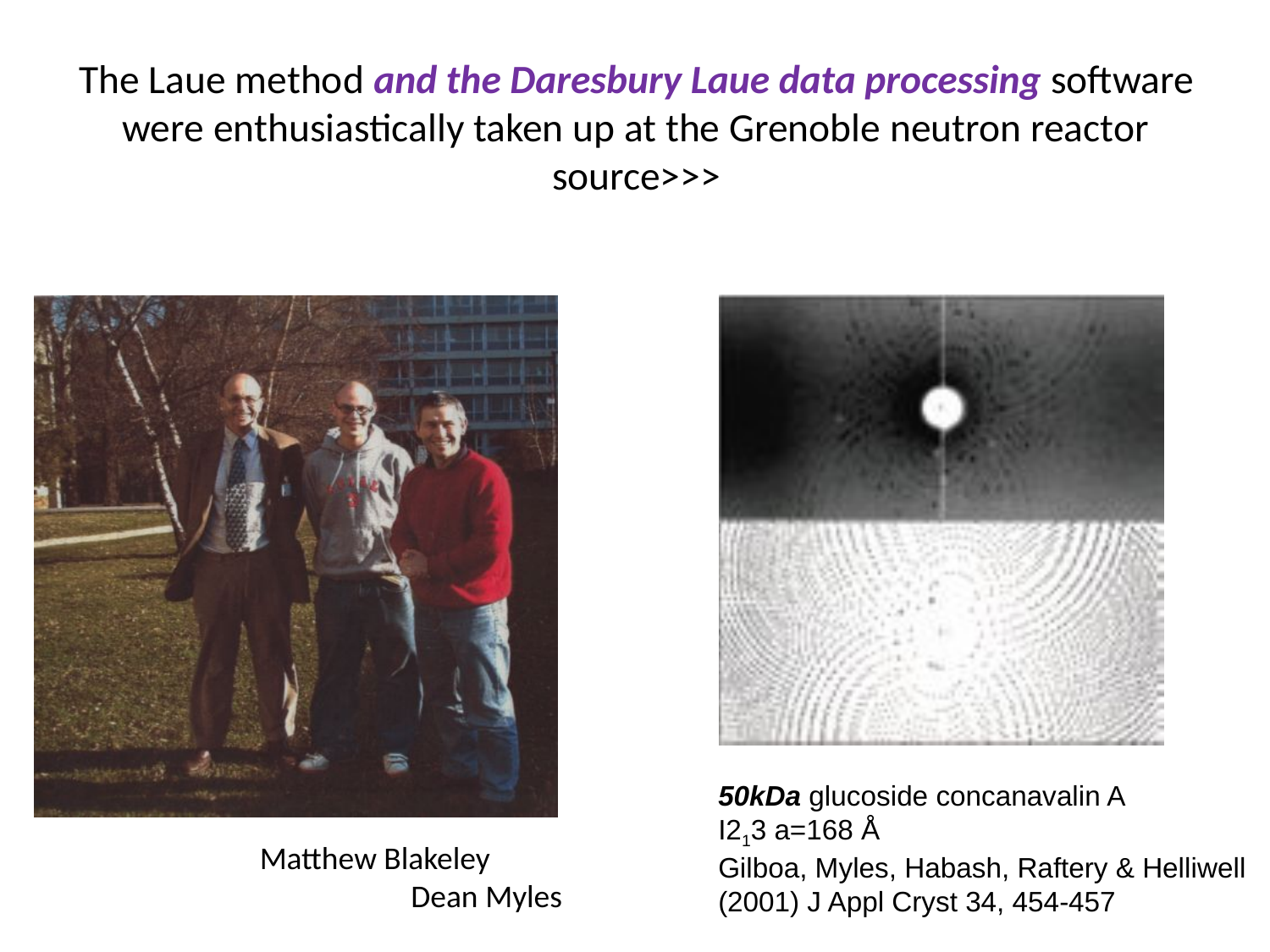

# The Laue method and the Daresbury Laue data processing software were enthusiastically taken up at the Grenoble neutron reactor source>>>
50kDa glucoside concanavalin A
I213 a=168 Å
Gilboa, Myles, Habash, Raftery & Helliwell
(2001) J Appl Cryst 34, 454-457
Matthew Blakeley
 Dean Myles

## Slide 27
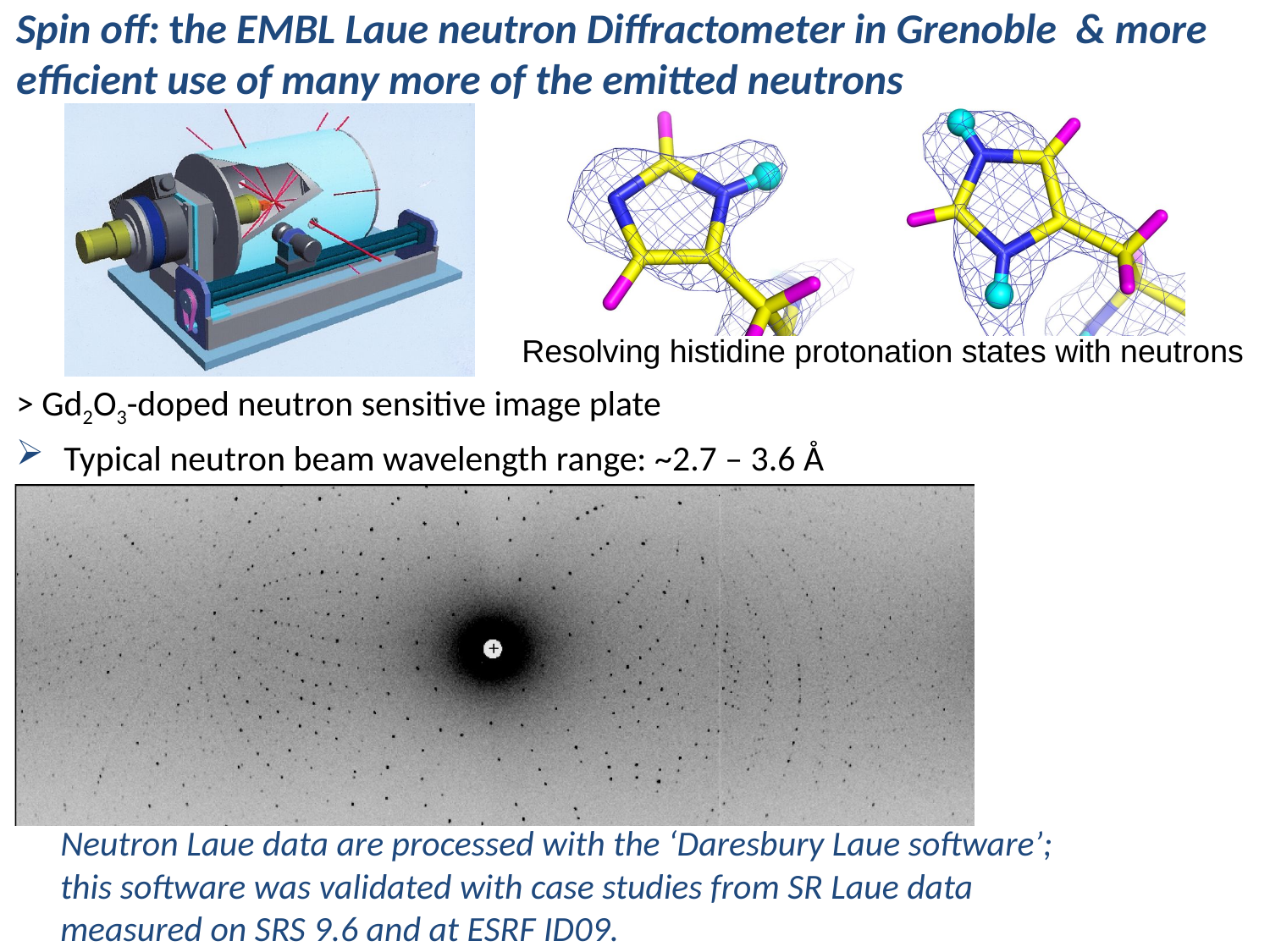

Spin off: the EMBL Laue neutron Diffractometer in Grenoble & more efficient use of many more of the emitted neutrons
Resolving histidine protonation states with neutrons
> Gd2O3-doped neutron sensitive image plate
Typical neutron beam wavelength range: ~2.7 – 3.6 Å
Neutron Laue data are processed with the ‘Daresbury Laue software’;
this software was validated with case studies from SR Laue data
measured on SRS 9.6 and at ESRF ID09.

## Slide 28
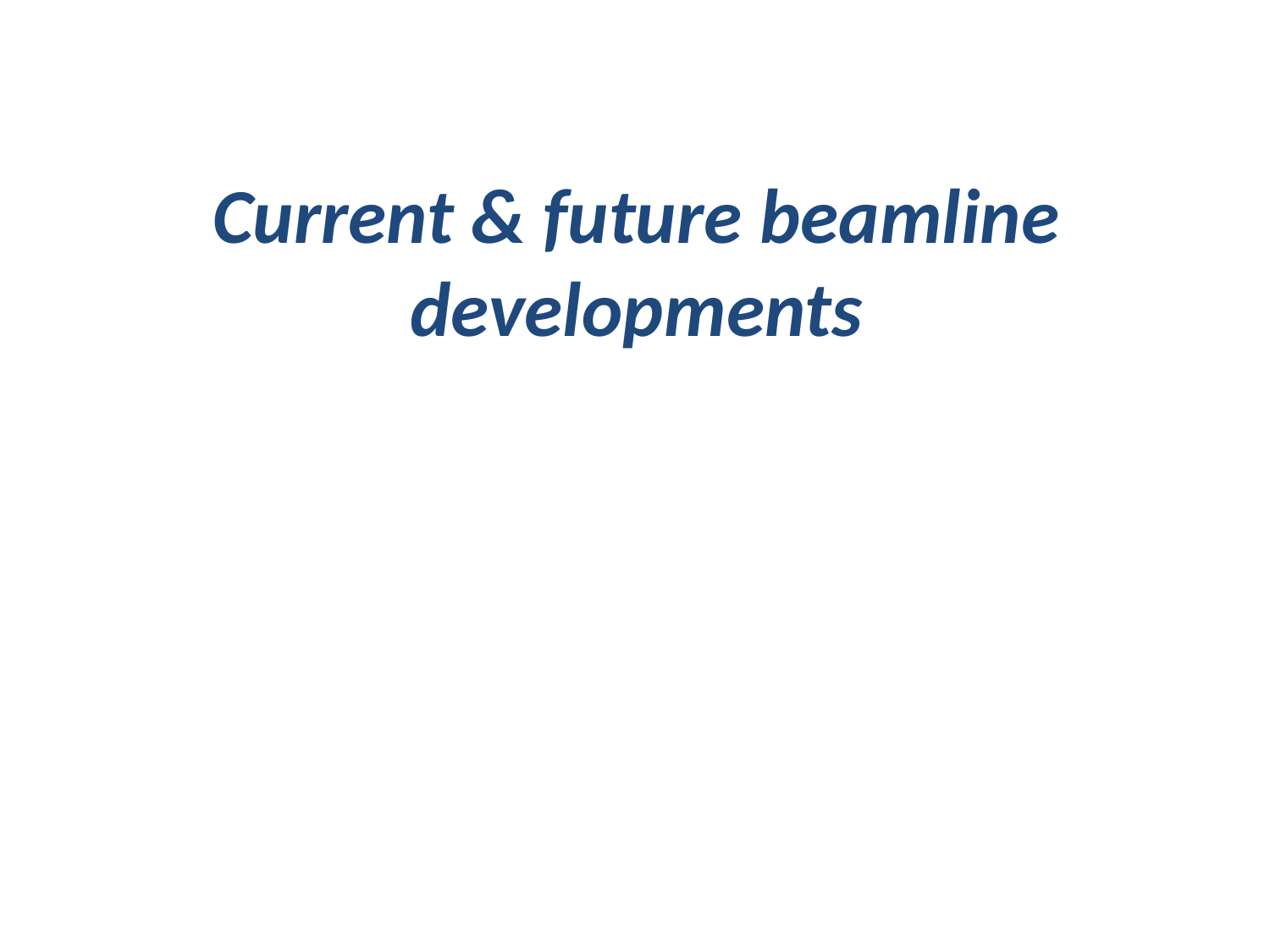

# Current & future beamline developments

## Slide 29
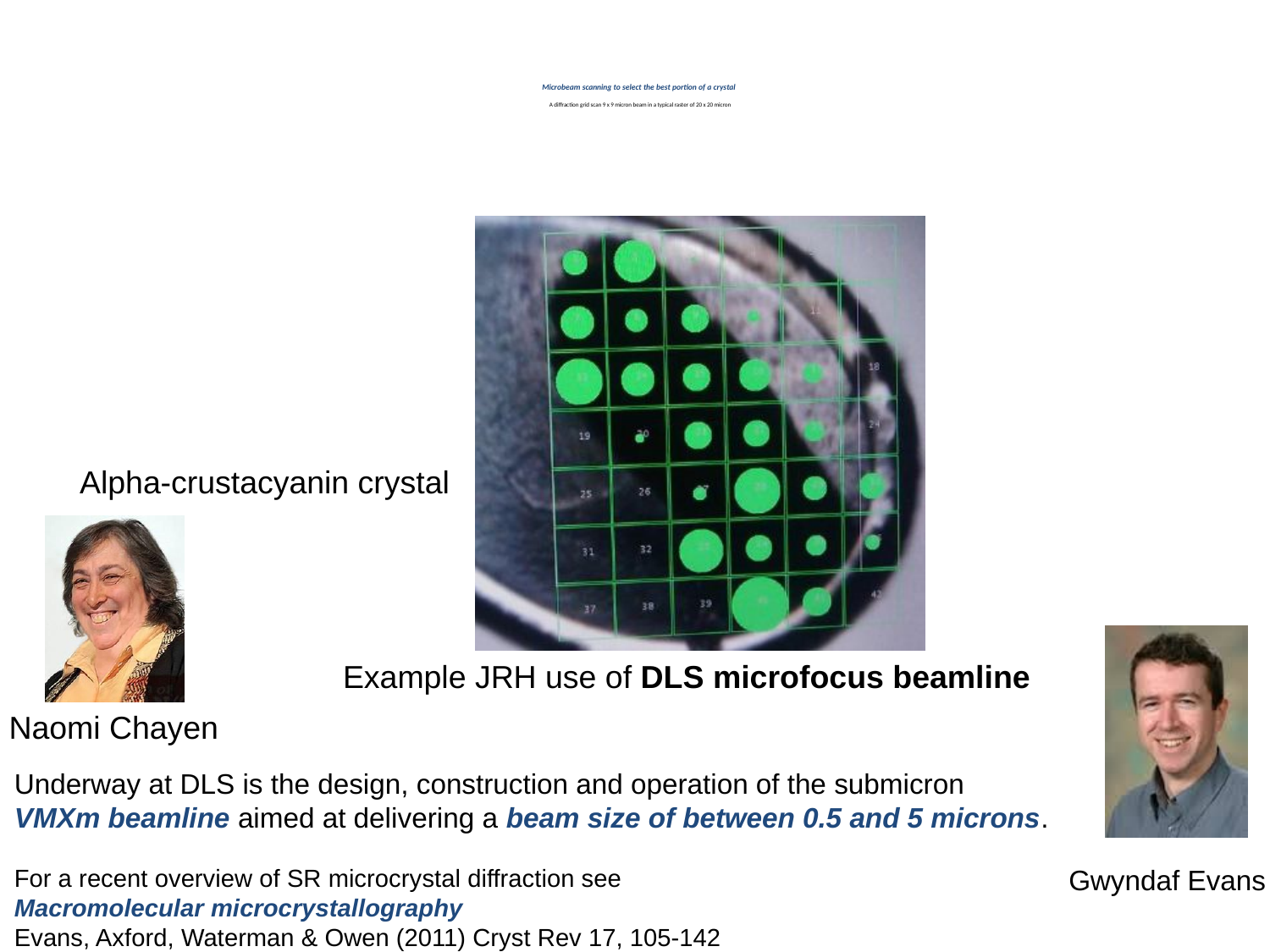

# Microbeam scanning to select the best portion of a crystal A diffraction grid scan 9 x 9 micron beam in a typical raster of 20 x 20 micron
Alpha-crustacyanin crystal
Example JRH use of DLS microfocus beamline
Naomi Chayen
Underway at DLS is the design, construction and operation of the submicron
VMXm beamline aimed at delivering a beam size of between 0.5 and 5 microns.
For a recent overview of SR microcrystal diffraction see
Macromolecular microcrystallography
Evans, Axford, Waterman & Owen (2011) Cryst Rev 17, 105-142
Gwyndaf Evans

## Slide 30
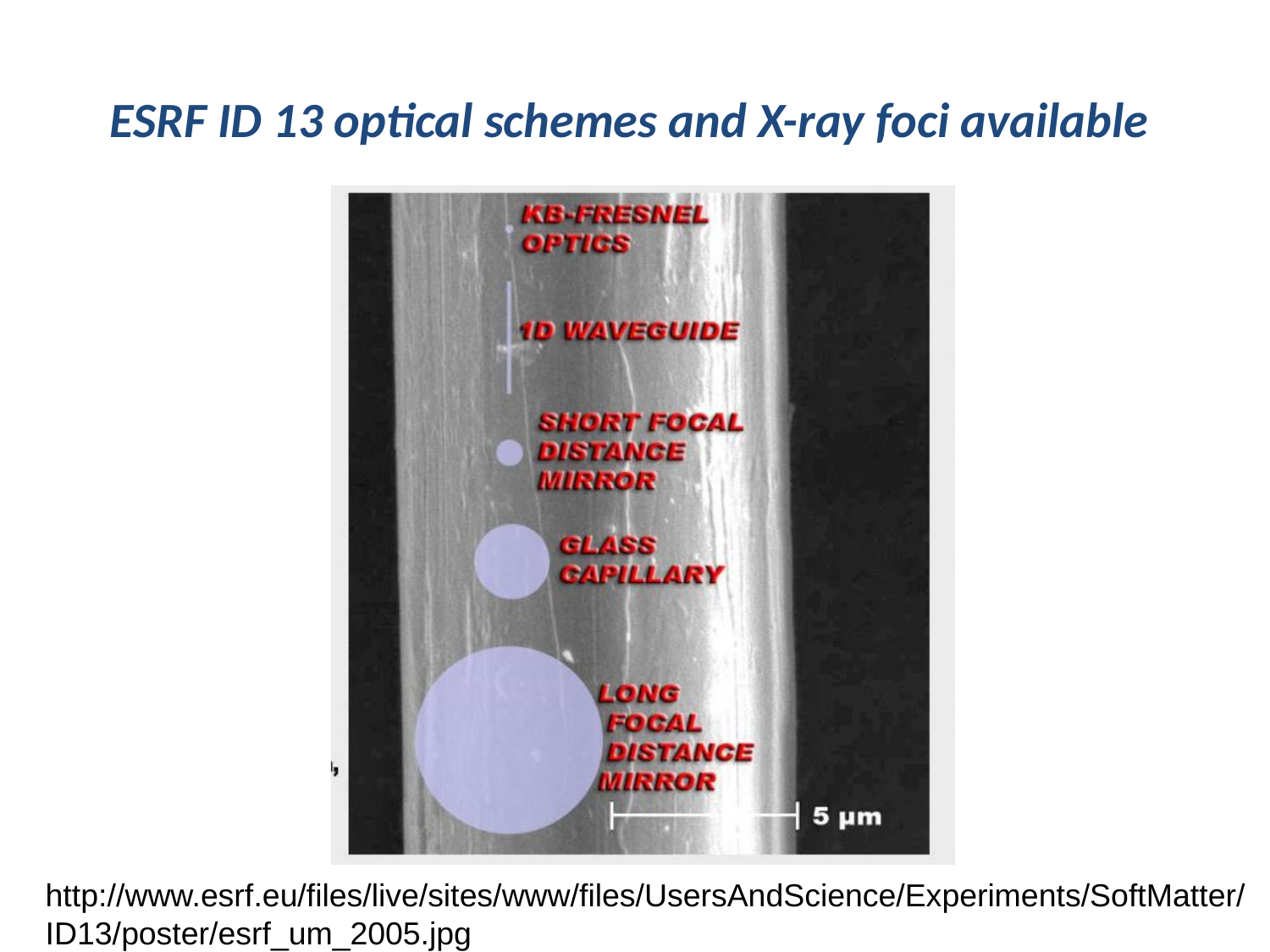

# ESRF ID 13 optical schemes and X-ray foci available
http://www.esrf.eu/files/live/sites/www/files/UsersAndScience/Experiments/SoftMatter/
ID13/poster/esrf_um_2005.jpg

## Slide 31
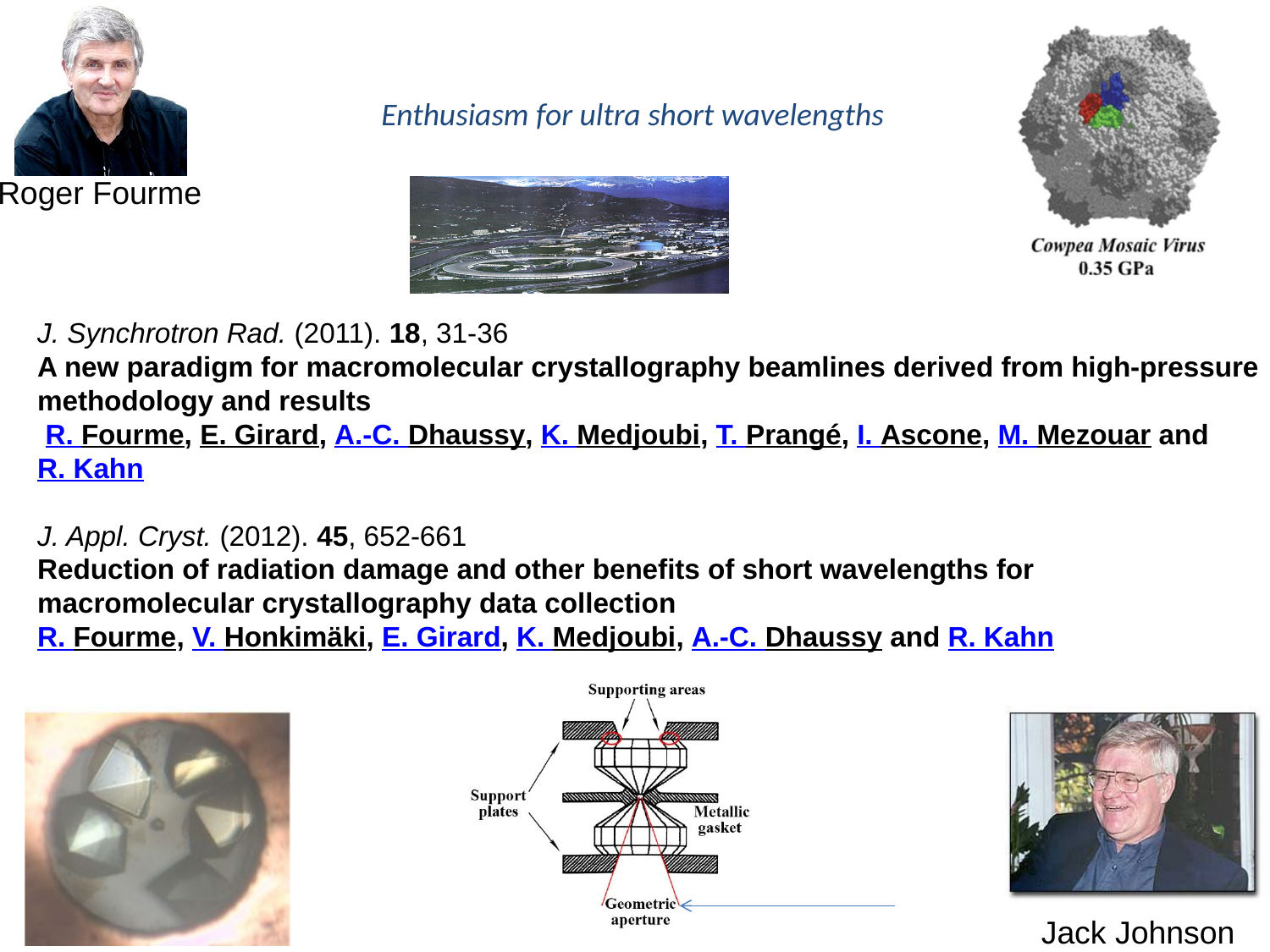

# Enthusiasm for ultra short wavelengths
Roger Fourme
J. Synchrotron Rad. (2011). 18, 31-36
A new paradigm for macromolecular crystallography beamlines derived from high-pressure methodology and results
 R. Fourme, E. Girard, A.-C. Dhaussy, K. Medjoubi, T. Prangé, I. Ascone, M. Mezouar and R. Kahn
J. Appl. Cryst. (2012). 45, 652-661
Reduction of radiation damage and other benefits of short wavelengths for
macromolecular crystallography data collection
R. Fourme, V. Honkimäki, E. Girard, K. Medjoubi, A.-C. Dhaussy and R. Kahn
Jack Johnson

## Slide 32
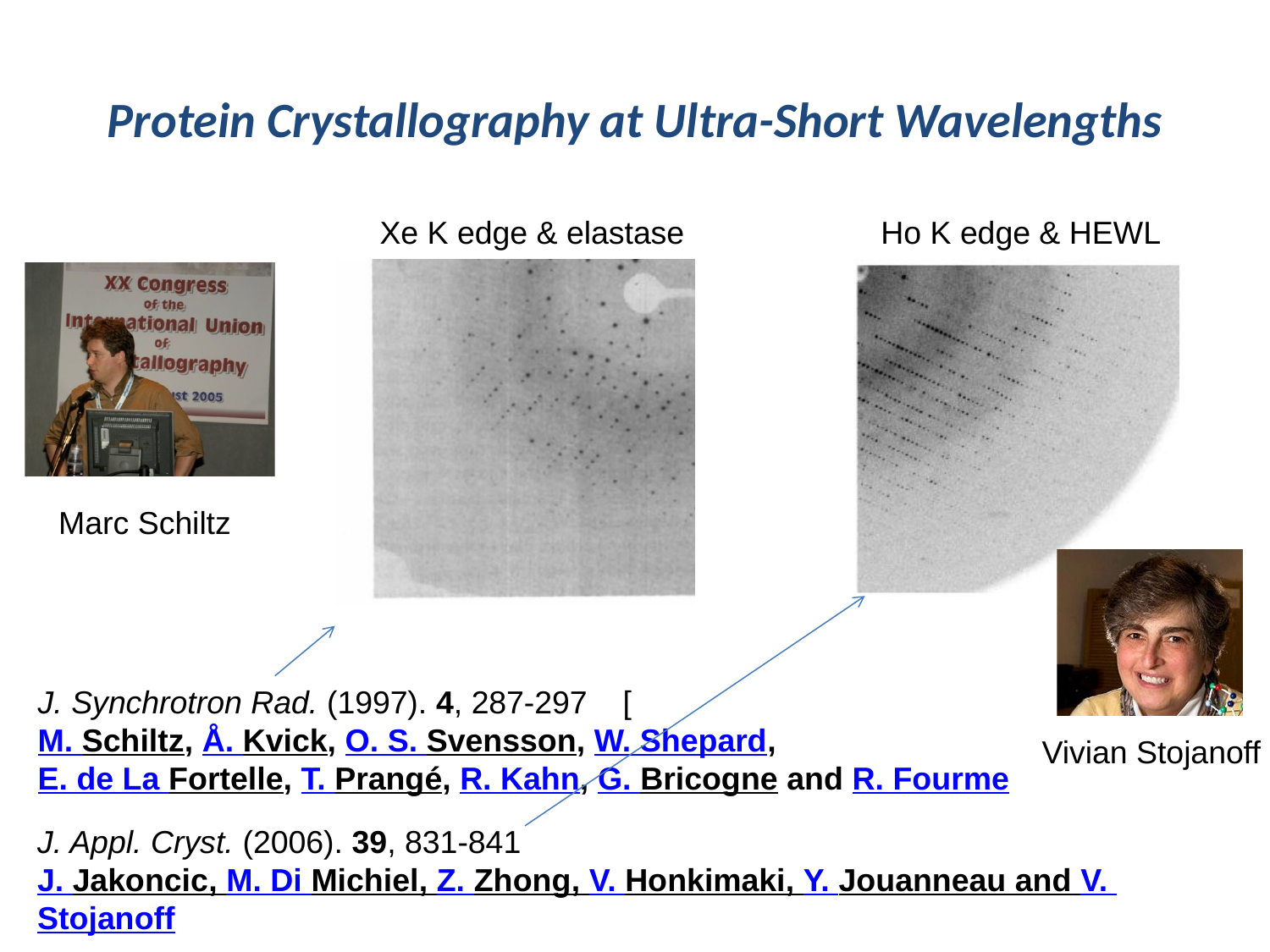

# Protein Crystallography at Ultra-Short Wavelengths
Xe K edge & elastase
Ho K edge & HEWL
Marc Schiltz
J. Synchrotron Rad. (1997). 4, 287-297    [
M. Schiltz, Å. Kvick, O. S. Svensson, W. Shepard,
E. de La Fortelle, T. Prangé, R. Kahn, G. Bricogne and R. Fourme
Vivian Stojanoff
J. Appl. Cryst. (2006). 39, 831-841
J. Jakoncic, M. Di Michiel, Z. Zhong, V. Honkimaki, Y. Jouanneau and V. Stojanoff

## Slide 33
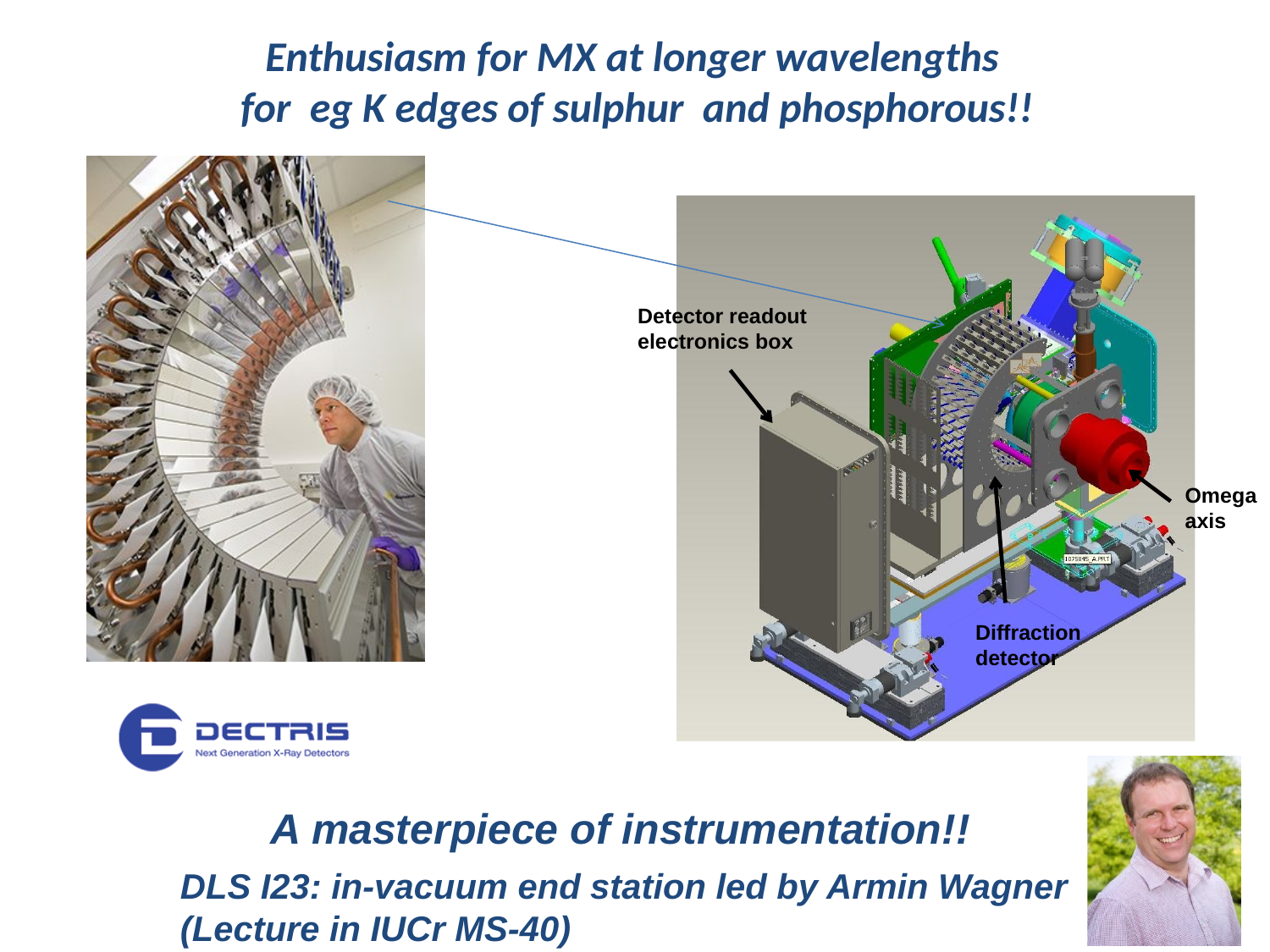

# Enthusiasm for MX at longer wavelengths for eg K edges of sulphur and phosphorous!!
Detector readout
electronics box
Omega
axis
Diffraction
detector
A masterpiece of instrumentation!!
DLS I23: in-vacuum end station led by Armin Wagner
(Lecture in IUCr MS-40)

## Slide 34
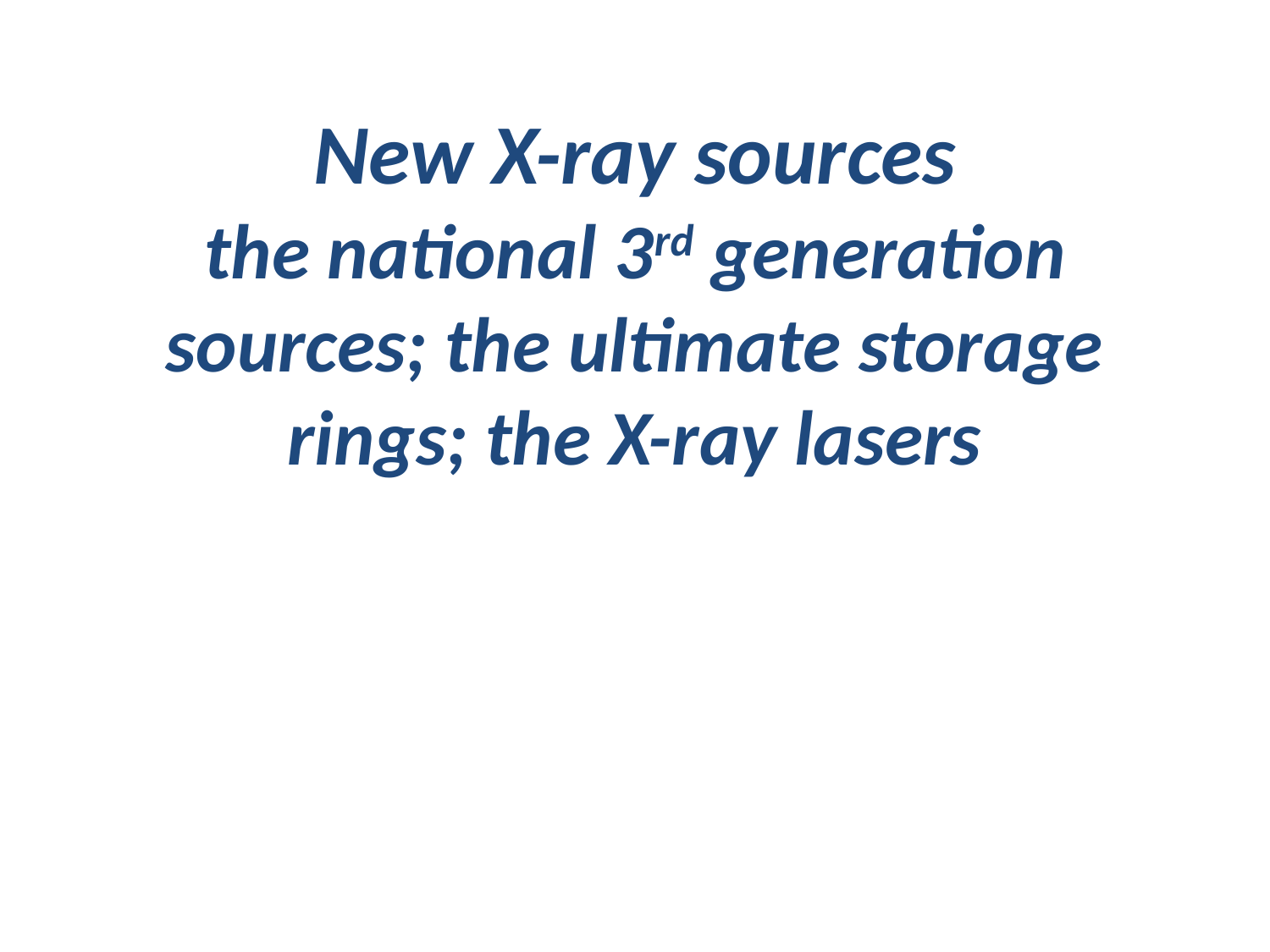

# New X-ray sources the national 3rd generation sources; the ultimate storage rings; the X-ray lasers

## Slide 35
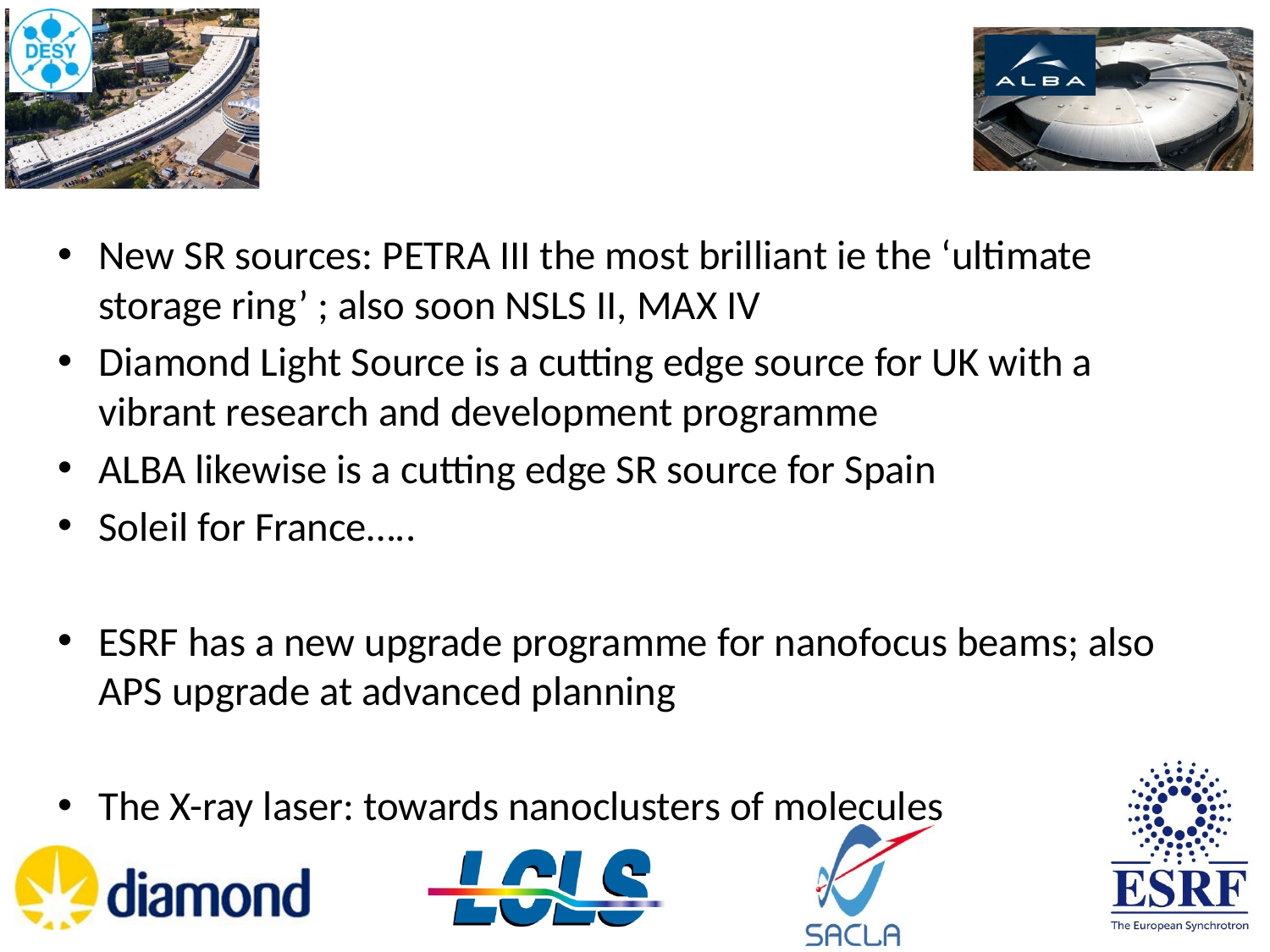

New SR sources: PETRA III the most brilliant ie the ‘ultimate storage ring’ ; also soon NSLS II, MAX IV
Diamond Light Source is a cutting edge source for UK with a vibrant research and development programme
ALBA likewise is a cutting edge SR source for Spain
Soleil for France…..
ESRF has a new upgrade programme for nanofocus beams; also APS upgrade at advanced planning
The X-ray laser: towards nanoclusters of molecules

## Slide 36
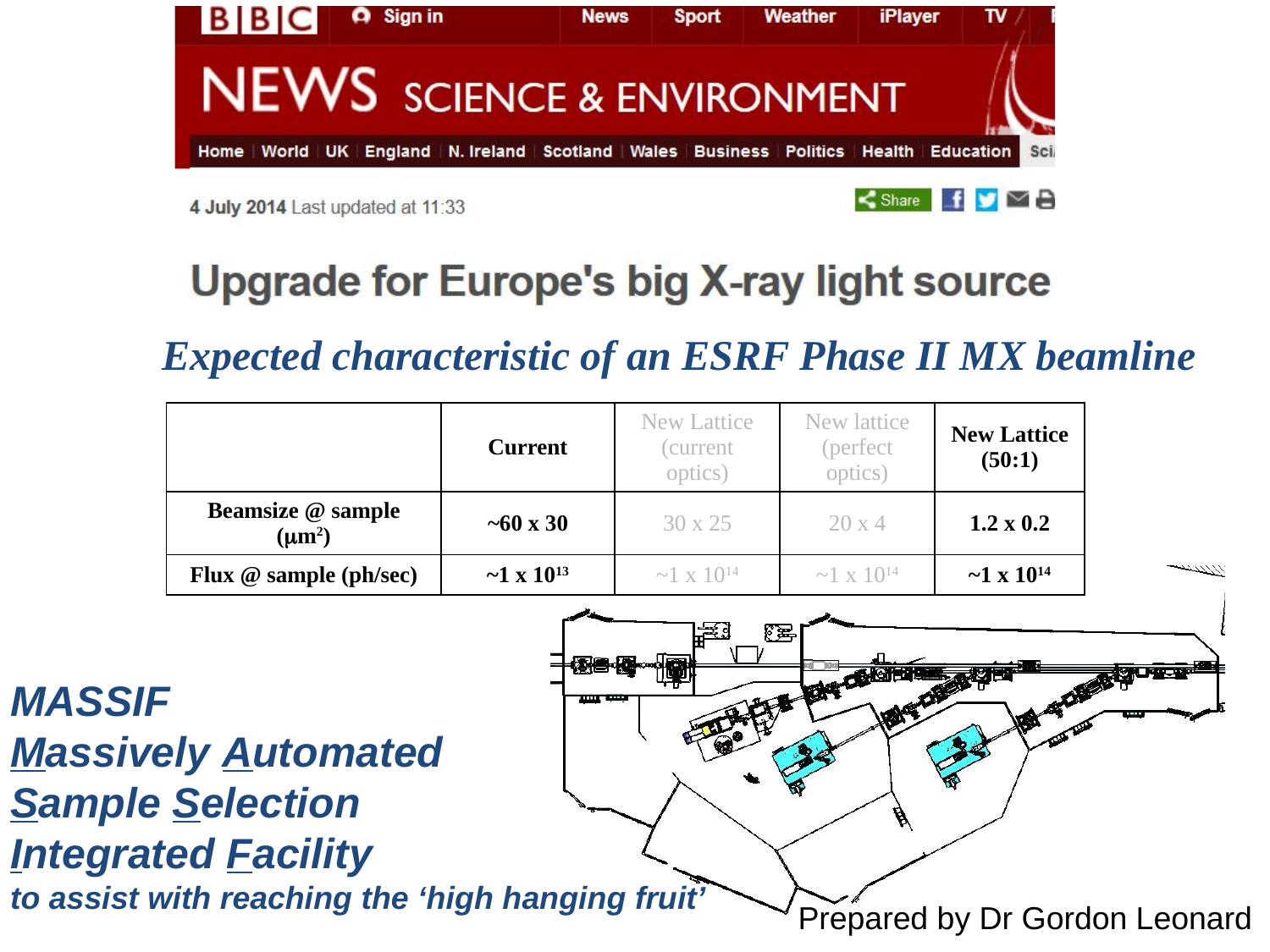

Expected characteristic of an ESRF Phase II MX beamline
| | Current | New Lattice (current optics) | New lattice (perfect optics) | New Lattice (50:1) |
| --- | --- | --- | --- | --- |
| Beamsize @ sample (mm2) | ~60 x 30 | 30 x 25 | 20 x 4 | 1.2 x 0.2 |
| Flux @ sample (ph/sec) | ~1 x 10^13^ | ~1 x 10^14^ | ~1 x 10^14^ | ~1 x 10^14^ |
MASSIF
Massively Automated
Sample Selection
Integrated Facility
to assist with reaching the ‘high hanging fruit’
Prepared by Dr Gordon Leonard

## Slide 37
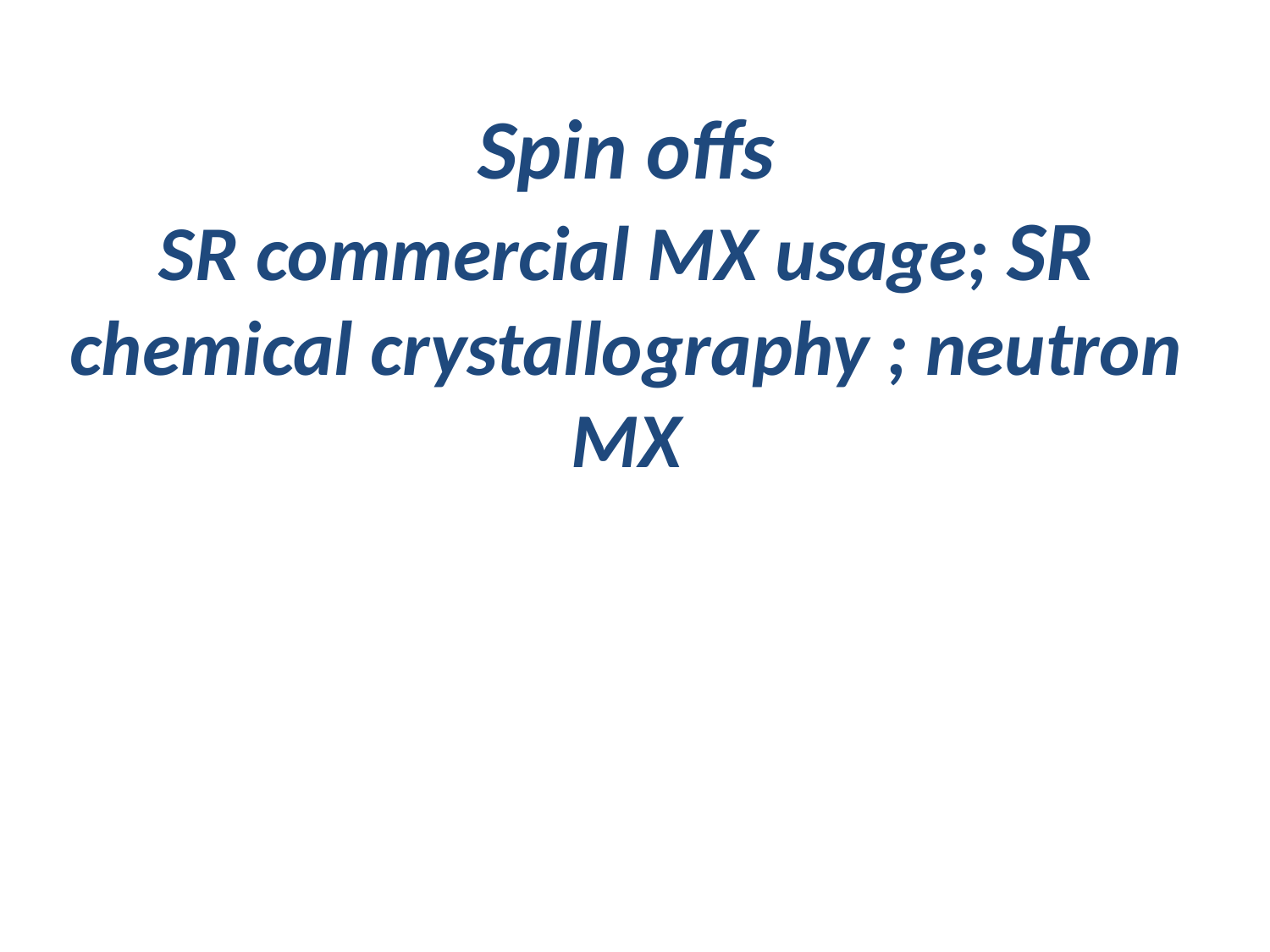

# Spin offs SR commercial MX usage; SR chemical crystallography ; neutron MX

## Slide 38
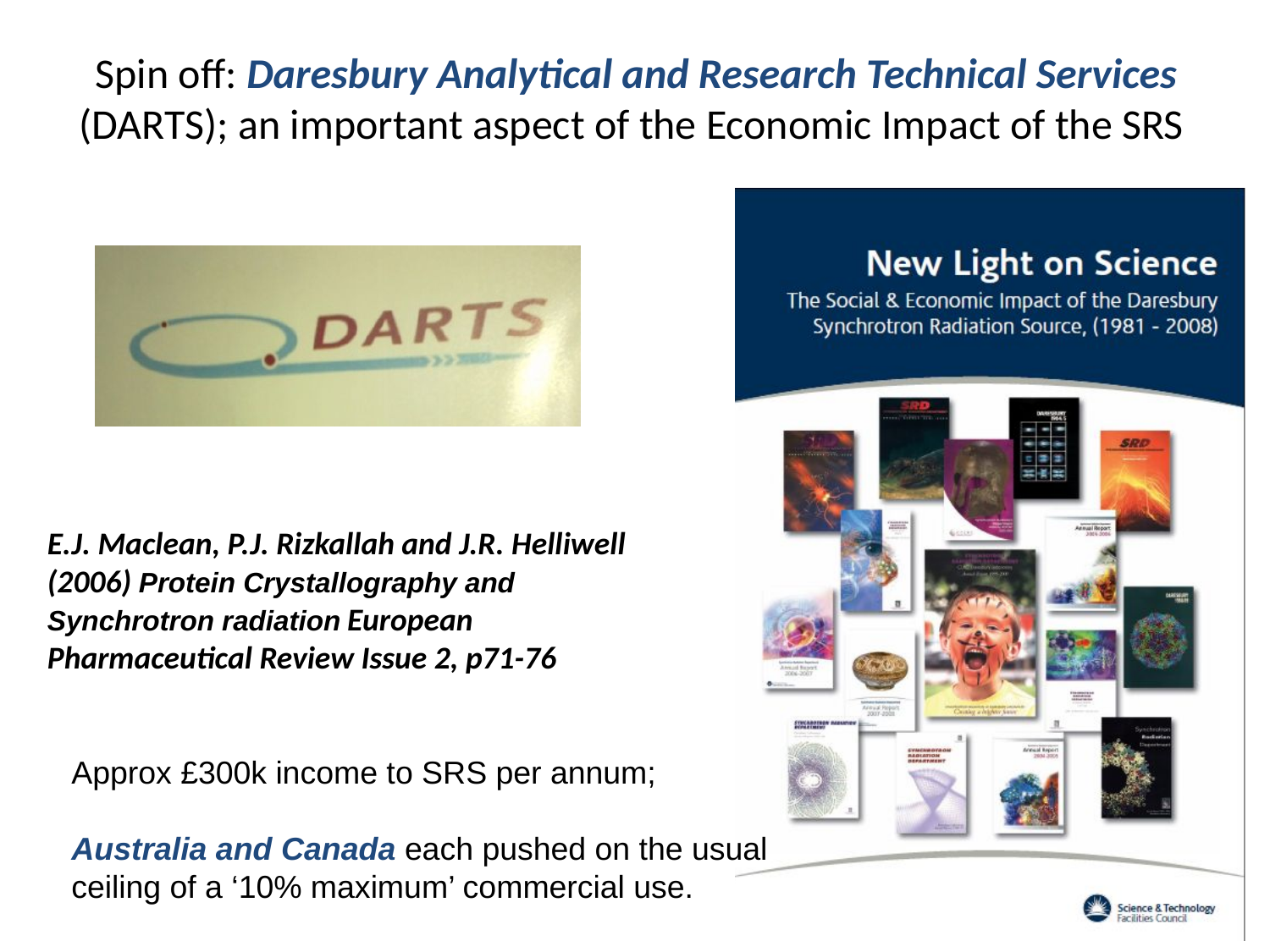

# Spin off: Daresbury Analytical and Research Technical Services (DARTS); an important aspect of the Economic Impact of the SRS
E.J. Maclean, P.J. Rizkallah and J.R. Helliwell (2006) Protein Crystallography and Synchrotron radiation European Pharmaceutical Review Issue 2, p71-76
Approx £300k income to SRS per annum;
Australia and Canada each pushed on the usual
ceiling of a ‘10% maximum’ commercial use.

## Slide 39
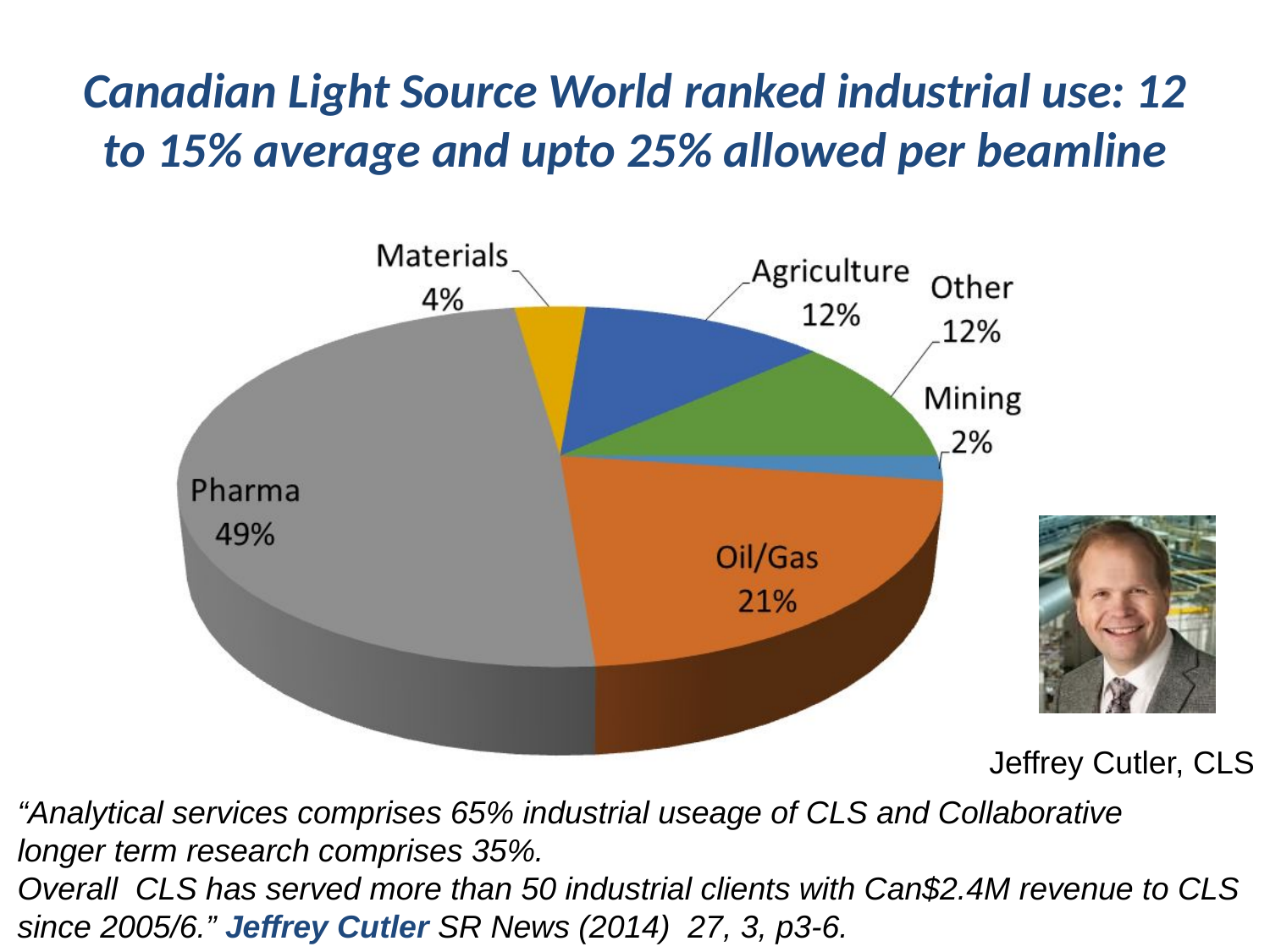

# Canadian Light Source World ranked industrial use: 12 to 15% average and upto 25% allowed per beamline
Jeffrey Cutler, CLS
“Analytical services comprises 65% industrial useage of CLS and Collaborative
longer term research comprises 35%.
Overall CLS has served more than 50 industrial clients with Can$2.4M revenue to CLS
since 2005/6.” Jeffrey Cutler SR News (2014) 27, 3, p3-6.

## Slide 40
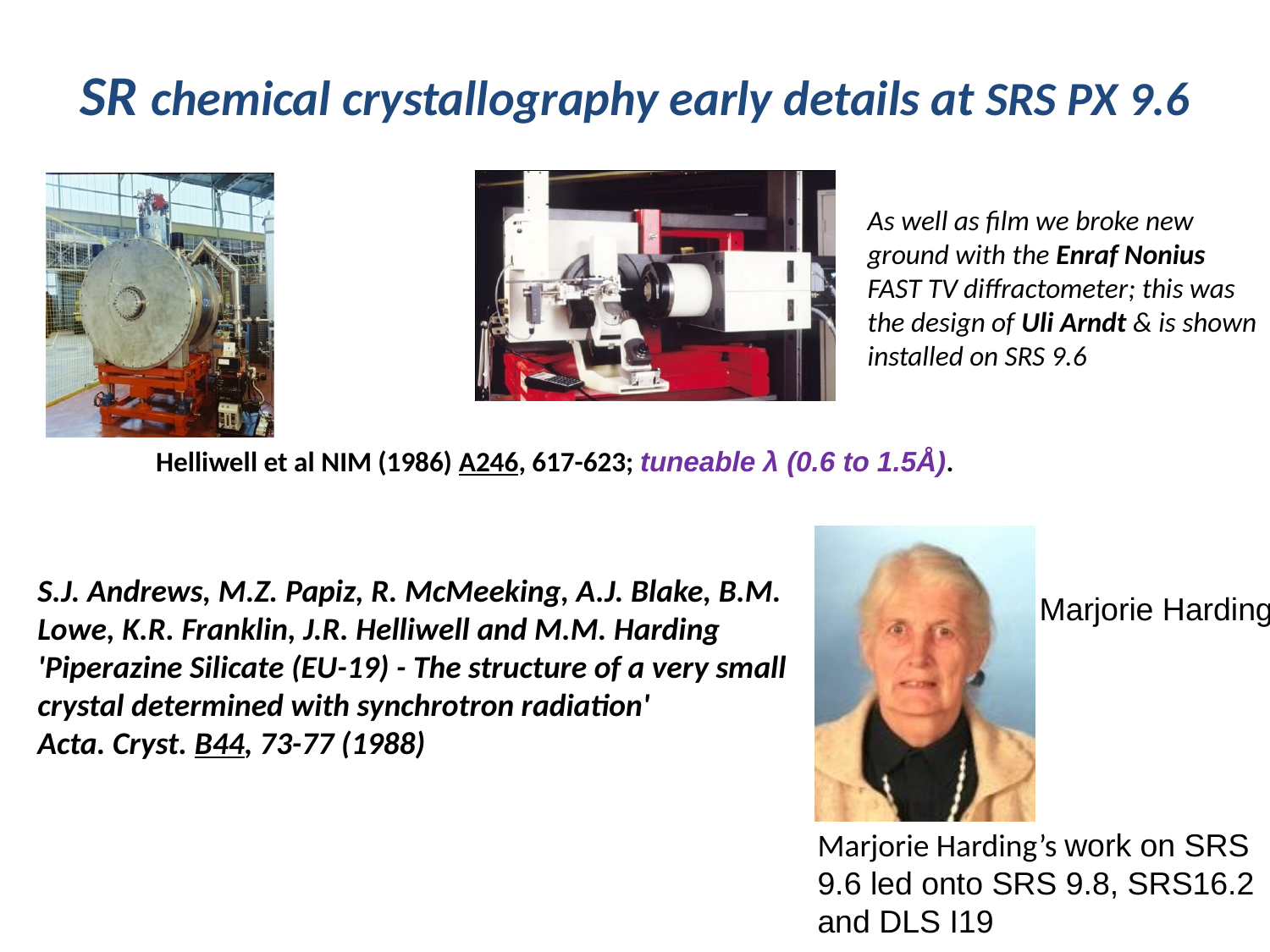

# SR chemical crystallography early details at SRS PX 9.6
As well as film we broke new ground with the Enraf Nonius FAST TV diffractometer; this was the design of Uli Arndt & is shown installed on SRS 9.6
Helliwell et al NIM (1986) A246, 617-623; tuneable λ (0.6 to 1.5Å).
S.J. Andrews, M.Z. Papiz, R. McMeeking, A.J. Blake, B.M. Lowe, K.R. Franklin, J.R. Helliwell and M.M. Harding 'Piperazine Silicate (EU-19) - The structure of a very small crystal determined with synchrotron radiation'
Acta. Cryst. B44, 73-77 (1988)
Marjorie Harding
Marjorie Harding’s work on SRS 9.6 led onto SRS 9.8, SRS16.2 and DLS I19

## Slide 41
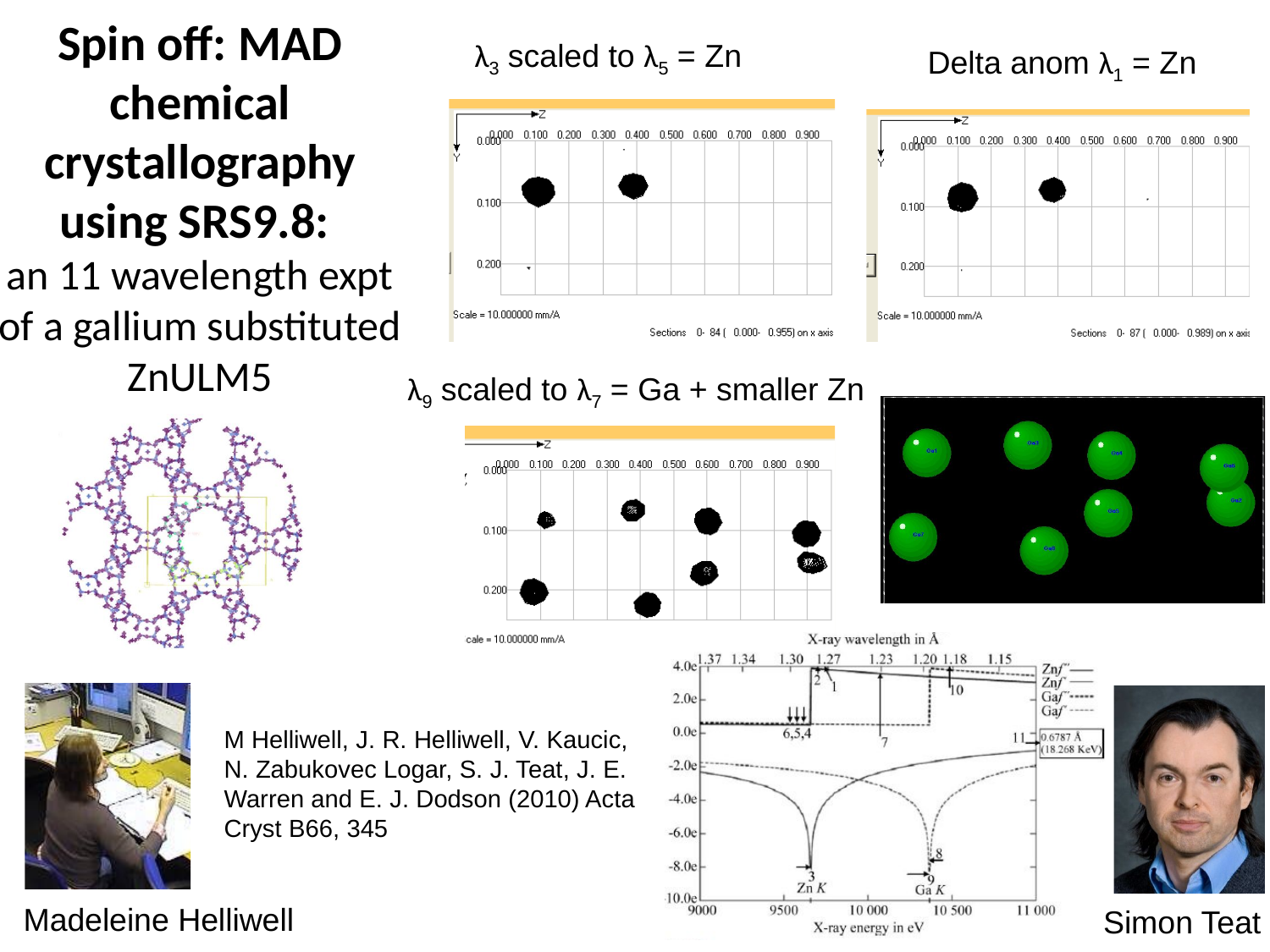

λ3 scaled to λ5 = Zn
Delta anom λ1 = Zn
Spin off: MAD chemical crystallography using SRS9.8:
an 11 wavelength expt of a gallium substituted ZnULM5
λ9 scaled to λ7 = Ga + smaller Zn
M Helliwell, J. R. Helliwell, V. Kaucic, N. Zabukovec Logar, S. J. Teat, J. E. Warren and E. J. Dodson (2010) Acta Cryst B66, 345
Madeleine Helliwell
Simon Teat

## Slide 42
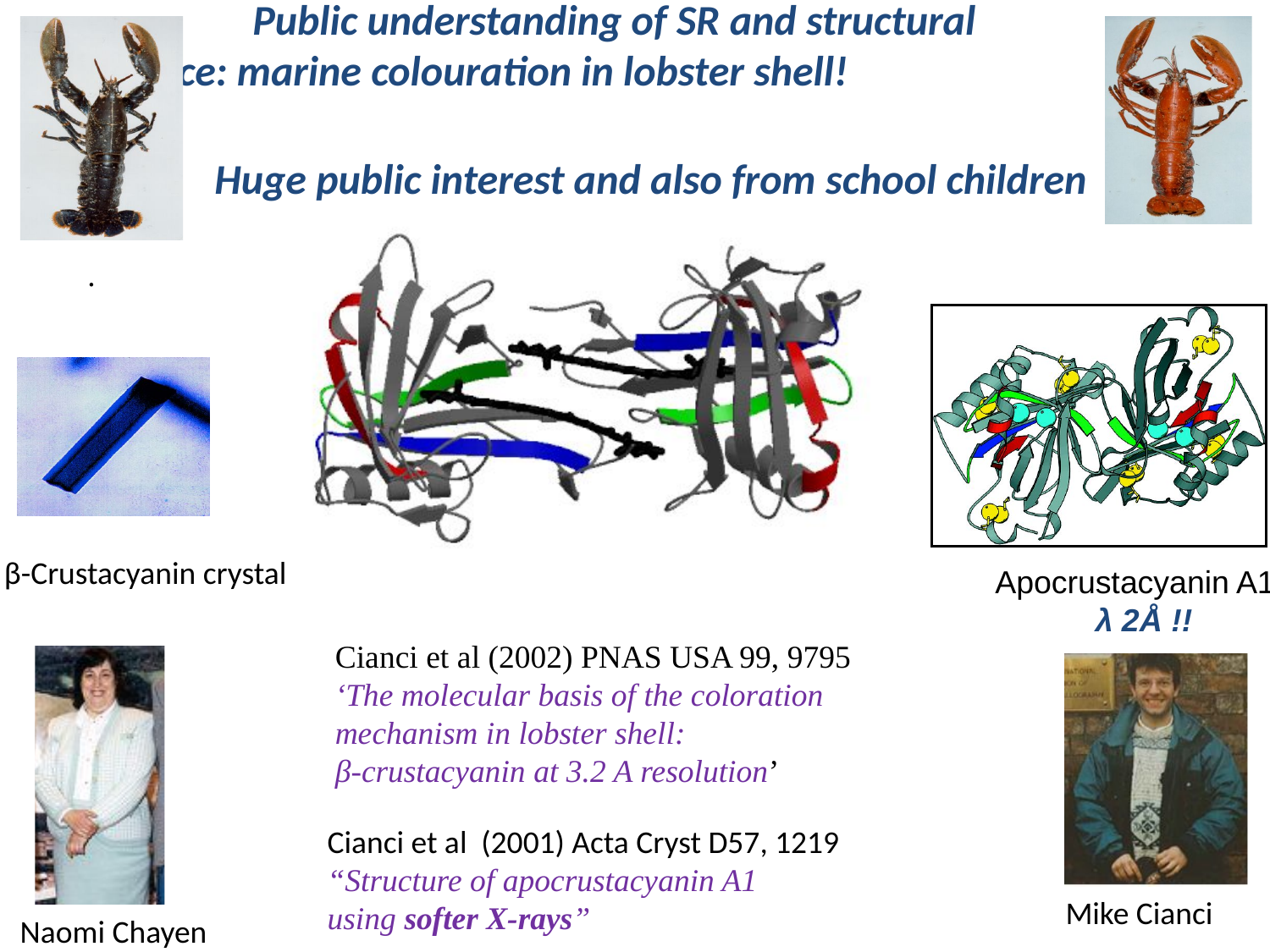

Public understanding of SR and structural 	Science: marine colouration in lobster shell!
	Huge public interest and also from school children
.
β-Crustacyanin crystal
 Apocrustacyanin A1
 	λ 2Å !!
Cianci et al (2002) PNAS USA 99, 9795 ‘The molecular basis of the coloration mechanism in lobster shell:
β-crustacyanin at 3.2 A resolution’
Cianci et al (2001) Acta Cryst D57, 1219
“Structure of apocrustacyanin A1
using softer X-rays”
 Mike Cianci
Naomi Chayen

## Slide 43
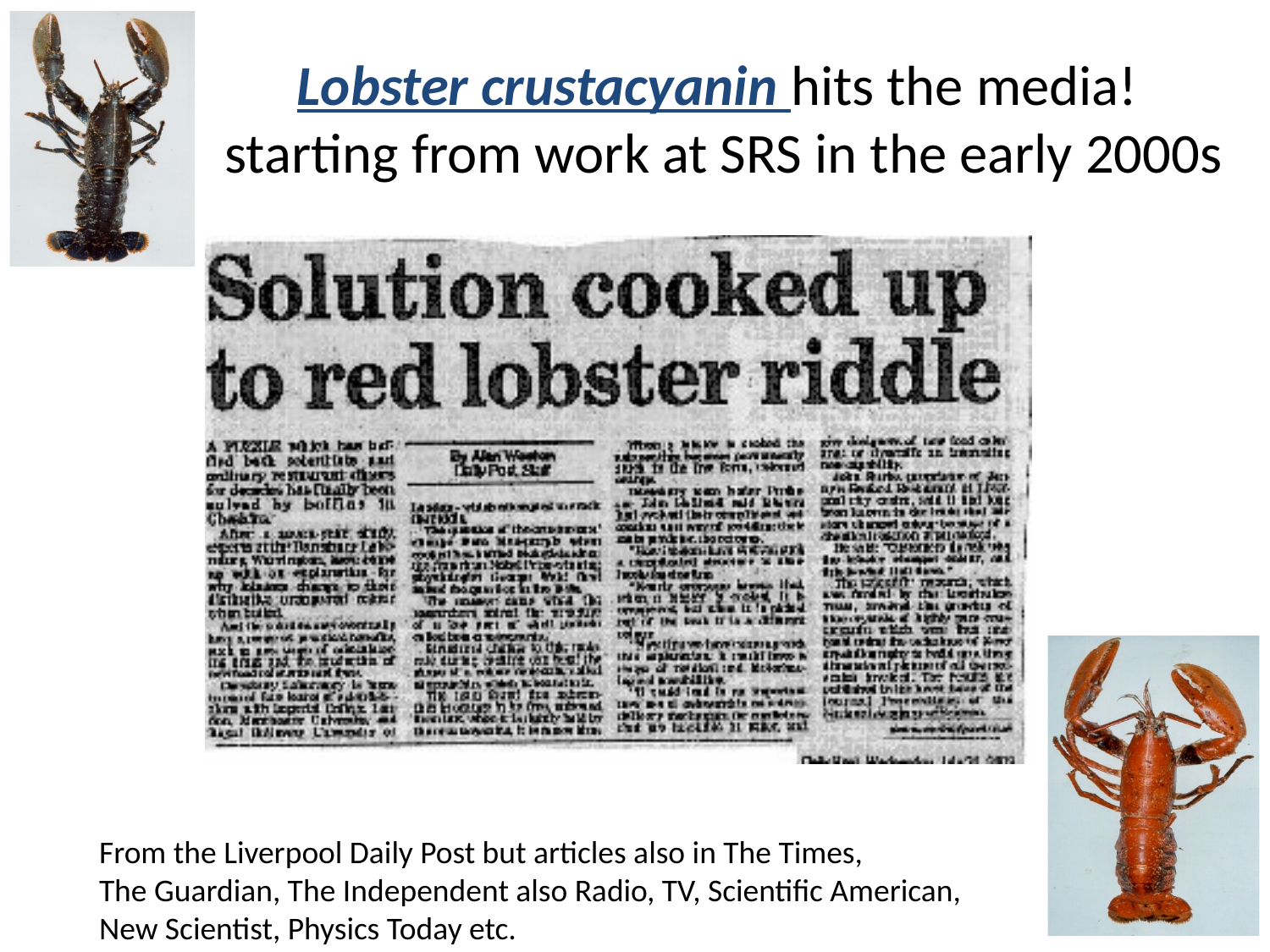

# Lobster crustacyanin hits the media! starting from work at SRS in the early 2000s
From the Liverpool Daily Post but articles also in The Times,
The Guardian, The Independent also Radio, TV, Scientific American,
New Scientist, Physics Today etc.

## Slide 44
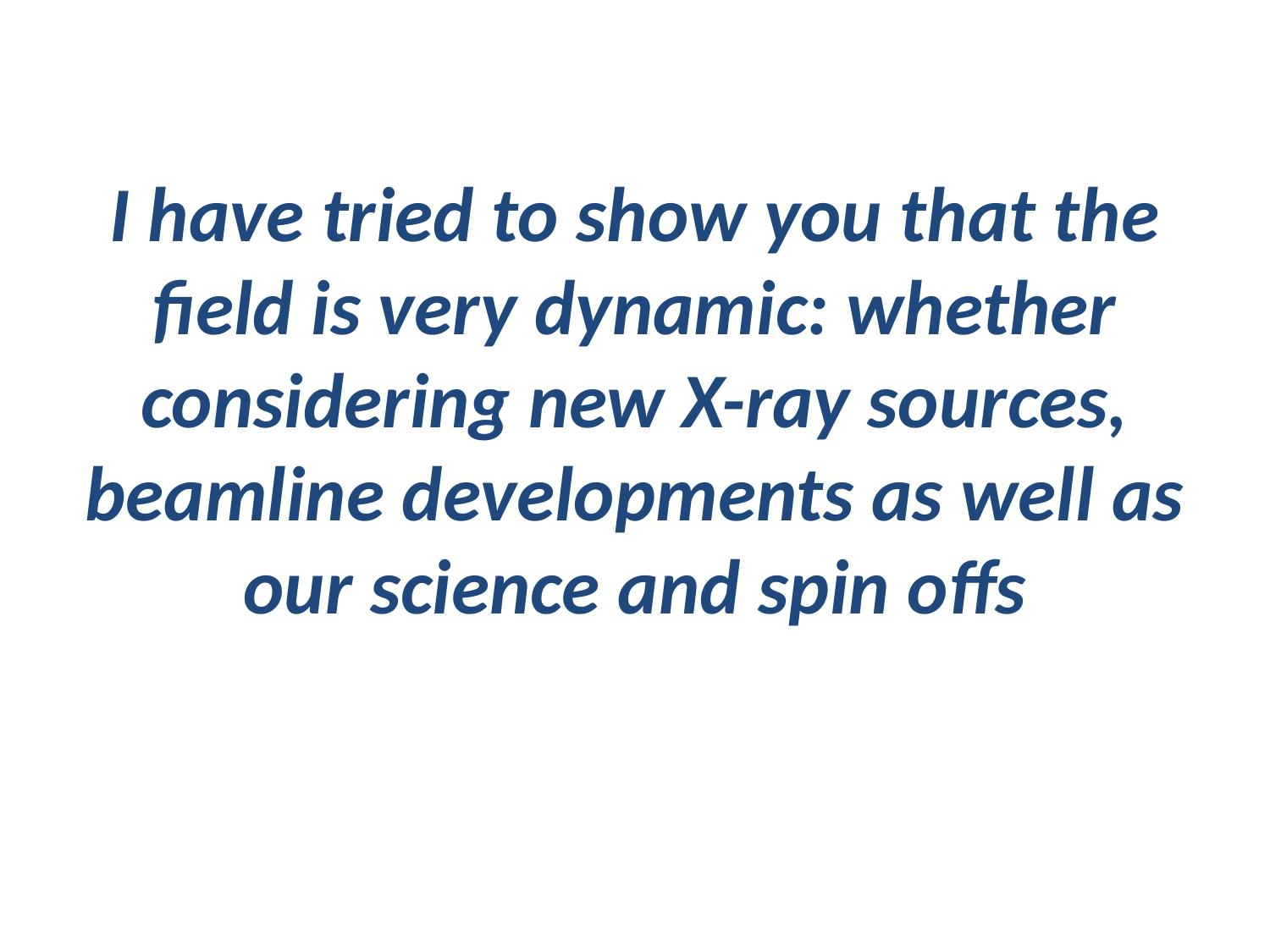

# I have tried to show you that the field is very dynamic: whether considering new X-ray sources, beamline developments as well as our science and spin offs

## Slide 45
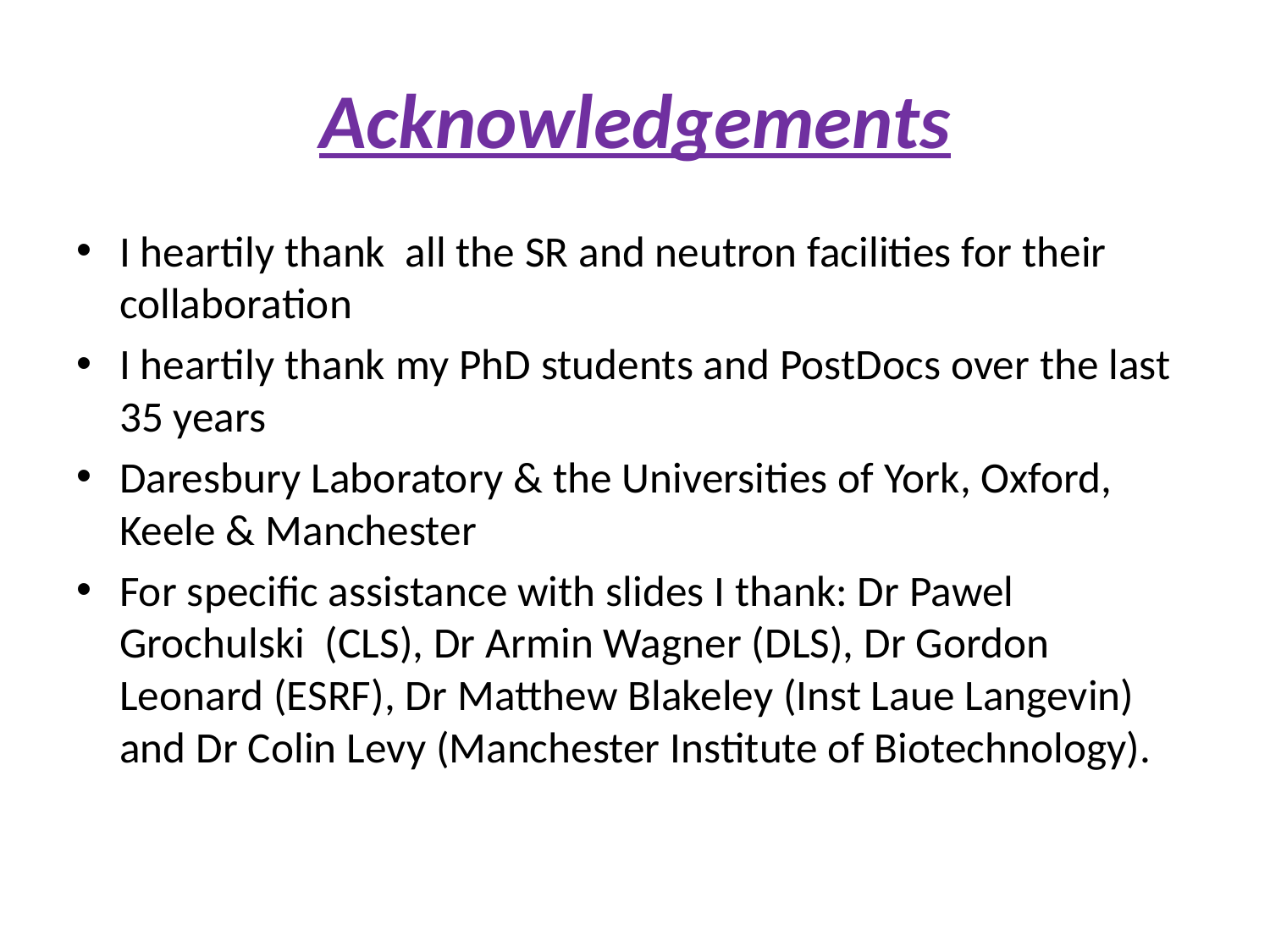

# Acknowledgements
I heartily thank all the SR and neutron facilities for their collaboration
I heartily thank my PhD students and PostDocs over the last 35 years
Daresbury Laboratory & the Universities of York, Oxford, Keele & Manchester
For specific assistance with slides I thank: Dr Pawel Grochulski (CLS), Dr Armin Wagner (DLS), Dr Gordon Leonard (ESRF), Dr Matthew Blakeley (Inst Laue Langevin) and Dr Colin Levy (Manchester Institute of Biotechnology).

## Slide 46
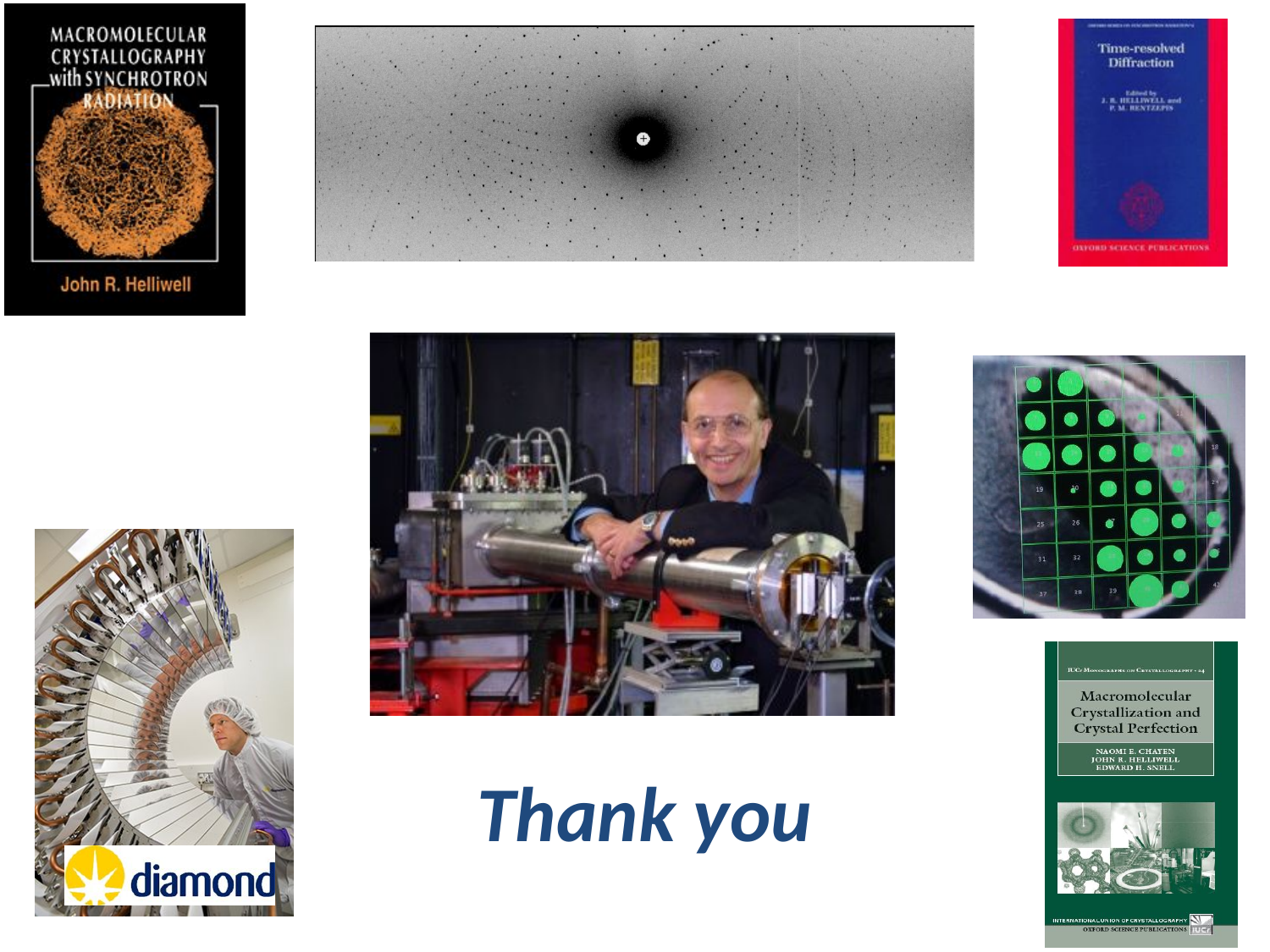

# Thank you
